# A Highly Efficient Bismuth Nitrate/Keto-ABNO Catalyst System for Aerobic Oxidation of Alcohols to Carbonyl Compounds under Mild Conditions

**DOI:** 10.3390/molecules27123727

**Published:** 2022-06-09

**Authors:** Yongke Hu, Lei Chen, Gulou Shen, Jin Li, Shaozhong Li, Huaju Li, Yanxing Li

**Affiliations:** 1 National & Local Joint Engineering Research Center for Mineral Salt Deep Utilization, Huaiyin Institute of Technology, Huaian 223003, China; lsheng@hyit.edu.cn (G.S.); lijin96998@hyit.edu.cn (J.L.); lsz114@hyit.edu.cn (S.L.); huajuli@hyit.edu.cn (H.L.); liyanxing@hyit.edu.cn (Y.L.); 2 Zhongbei College, Nanjing Normal University, Zhenjiang 212300, China; zb_chenlei@163.com

**Keywords:** carbonyl compounds, aerobic oxidation, bismuth nitrate, keto-ABNO

## Abstract

An efficient and practical catalytic system for the oxidation of alcohols to aldehydes/ketones using catalytic amounts of Bi(NO_3_)_3_ and Keto-ABNO (9-azabicyclo [3.3.1]nonan-3-one *N*-oxyl) with air as the environmentally benign oxidant was developed. Various primary and secondary alcohols were smoothly oxidized to the corresponding products under mild conditions, and satisfactory yields were achieved. Moreover, this methodology avoids the use of a ligand and base. The gram-scale reaction was demonstrated for the oxidation of 1-phenyl ethanol, and the product of acetophenone was obtained at an isolated yield of about 94%.

## 1. Introduction

Aldehydes and ketones, as common and important organic intermediates, are widely found in pharmaceuticals, fragrances, vitamins, materials, and other fine chemicals. They are usually synthesized via the oxidation of alcohols, and a number of classical methods have been developed for this transformation [1,2,3]. However, most of them require at least stoichiometric amounts of oxidants, such as MnO_2_ [4], CrO_3_ [5], activated DMSO [6], and I_2_O_5_ [7], which generate an equal amount of waste, resulting in serious pollution to the environment. From both environmental and economic perspectives, molecular oxygen, which acts as a green oxidant, has attracted intensive attention, mainly owing to its abundance, low cost, and the possibility it offers of avoiding stoichiometric poisonous byproducts [8,9]. In light of this, a large number of transition-metal-catalyzed systems for the aerobic oxidation of alcohols with O_2_ or air as the terminal oxidant have been reported [10,11,12,13]. For most of these catalytic systems, noble metals (e.g., Pd [14], Ru [15], and Au [16]) or commercially unavailable ligands are required [17,18], which limit their applications. By contrast, combinations of stable nitroxyl radicals such as 2,2,6,6-tetramethylpiperidine-1-oxyl (TEMPO), 4-acetamido-TEMPO, 2-Azaadamantane *N*-oxyl (AZADO), 9-azabicyclo-[3.3.1]nonan-*N*-oxyl (ABNO), and 9-azabicyclo [3.3.1]nonan-3-one *N*-oxyl (Keto-ABNO) with co-catalysts have been developed for the aerobic oxidation of alcohols using O_2_ as the benign oxidant. Intensive research efforts have helped to maintain the focus on this field [19,20,21,22].

**Scheme 1:**
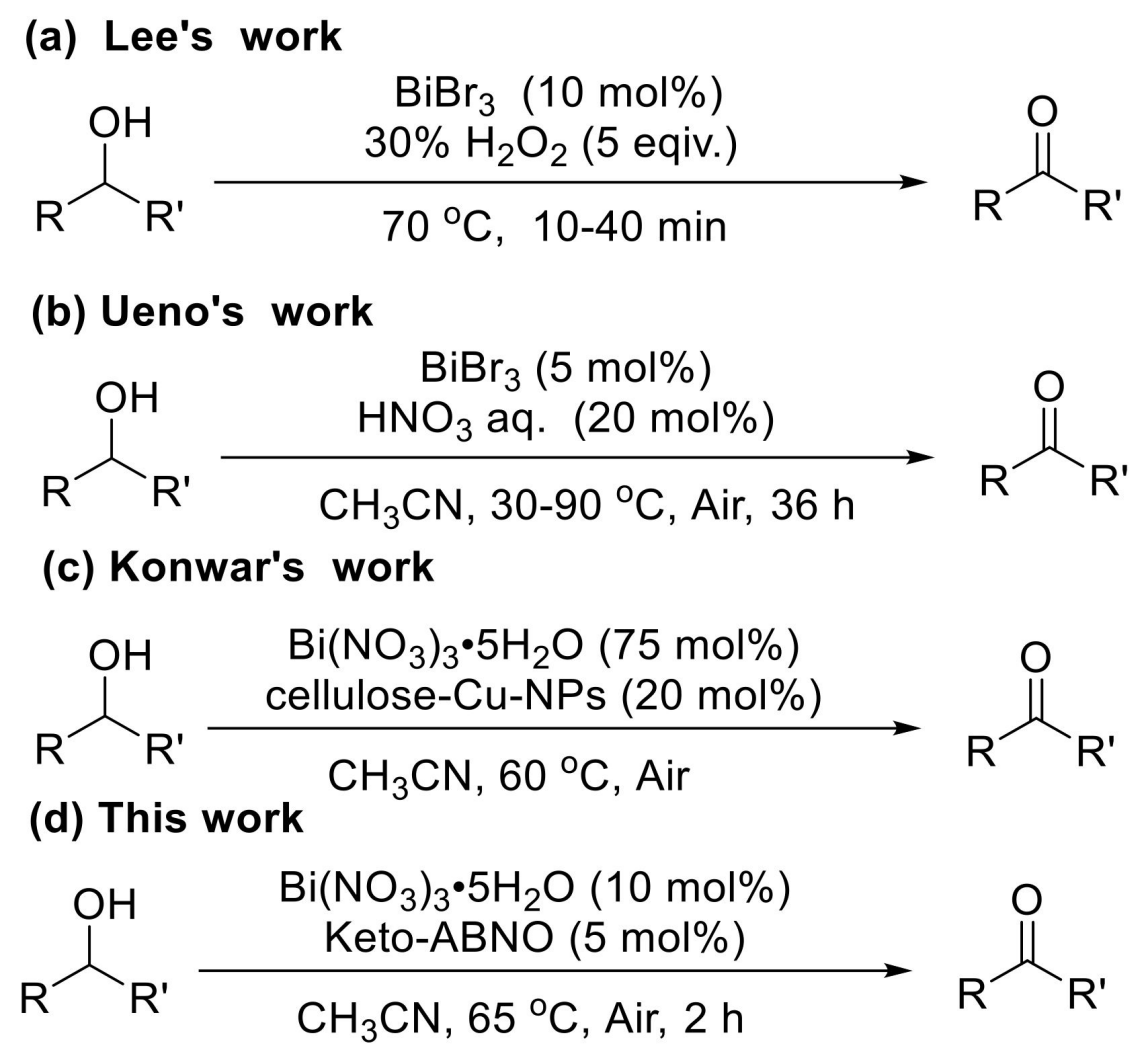
Bi-catalyzed aerobic oxidations of alcohols to aldehydes/ketones.

Bismuth, as a non-toxic, cheap, and readily available metal, has been applied as a suitable ecofriendly reagent in cosmetic and medicinal chemistry. Moreover, due to the non-toxicity and inexpensive characteristics of bismuth and its compounds, they are also used as efficient catalysts for many organic reactions [23,24,25,26,27,28]. Recently, our group employed bismuth(III) salts as a highly efficient co-catalyst for the direct oxidative esterification of alcohols [29]. Chakraborty et al. reported a simple Bi_2_O_3_-catalyzed alcohol oxidation method. However, a large amount of *tert*-butyl hydroperoxide was used as the oxidant, and the primary alcohols were over-oxidized to carboxylic acids [30]. Lee et al. reported a simple solvent-free methodology for alcohol oxidation catalyzed by bismuth bromide with aqueous hydrogen peroxide as the oxidant (Scheme 1a) [31]. Recently, Masaharu Ueno et al. described an environmentally friendly strategy for selective aerobic alcohol oxidation using relatively benign BiBr_3_ and HNO_3_ as catalysts (Scheme 1b) [32]. Mukhopadhyay et al. reported a procedure for the oxidation of aromatic aldehydes to carboxylic acids by stoichiometric bismuth(III) nitrate pentahydrate, along with aerial oxygen under solvent-free conditions [33]. In their report, no reaction was observed with molecular oxygen alone, indicating that the active species was produced in situ from Bi(NO_3_)_3_. Furthermore, Diganta Baruah et al., developed a versatile catalytic system combining 75 mol% Bi(NO_3_)_3_·5H_2_O with 20 mol% cellulose-mediated Cu-NPs for the oxidation of aromatic alcohols to carbonyl compounds (Scheme 1c) [34]. Although the oxidizing ability of bismuth catalysts is expected, the selective aerobic oxidation of alcohols with catalytic amounts of bismuth salts employing molecular oxygen as the terminal oxidant has not yet been developed. Therefore, the development of an efficient, selective, and eco-friendly protocol for the aerobic oxidation of alcohols using non-toxic, readily available bismuth catalysts is still desirable in both academia and industry.

Herein, on the basis of our continuous research interest in aerobic oxidation reactions [35,36,37,38,39], we describe a simple, efficient, and versatile strategy for the oxidation of alcohols to aldehydes/ketones by using 5 mol% Keto-ABNO as the catalyst and 10 mol% Bi(NO_3_)_3_·5H_2_O as the co-catalyst, with air as the environmentally benign oxidant under mild conditions. This catalyst system exhibited excellent catalytic ability, and various primary and secondary alcohols were smoothly converted to the desired products in moderate-to-good yields.

## 2. Results and Discussion

Initially, we optimized the reaction conditions by employing benzyl alcohol (**1a**) as a model substrate under air-balloon conditions at 65 °C. As shown in Table 1, no product was observed, while the reaction was performed in the absence of the bismuth catalyst and nitroxyl radical (Table 1, entries 1 and 2). This indicates the significant role of the bismuth catalyst and nitroxyl radical in this reaction. A series of bismuth catalysts, such as Bi_2_(SO_4_)_3_, Bi_2_O_3_, BiBr_3_, Bi(OTf)_3_, and Bi(NO_3_)_3_ were investigated in the presence of TEMPO (3 mol%). The Bi(NO_3_)_3_ demonstrated the highest activity, producing an isolated yield of 89% (Table 1, entries 3–7). In order to improve the efficiencies and selectivity of the catalyst system, the influence of different varieties of nitroxyl radical, such as TEMPO, 4-OH-TEMPO, 4-Acetamido-TEMPO (ACT), and Keto-ABNO were screened. To our delight, Keto-ABNO turned out to perform best in the model reaction (Table 1, entries 8–10). Next, we tested the effect of the catalyst dosage and reaction time on the transformation. It was found that a 2-h reaction time and dosages of 5 mol% of Keto-ABNO and 10 mol% of Bi(NO_3_)_3_ were the most effective (Table 1, entries 11–13). Subsequently, the influences of different solvents were examined. CH_3_CN afforded the highest yield compared with other solvents, such as H_2_O, DCE, DMSO, and ethanol (Table 1, entries 14–17). In addition, we carried out the reaction under an O_2_ atmosphere, the result of which was the same as that of the reaction under air (Table 1, entry 18). However, when the reaction was conducted under a nitrogen atmosphere, a yield of only 15% was obtained for **2a**, which confirmed that molecular oxygen was an essential oxidant for the reaction (Table 1, entry 19).

**Scheme 2:**
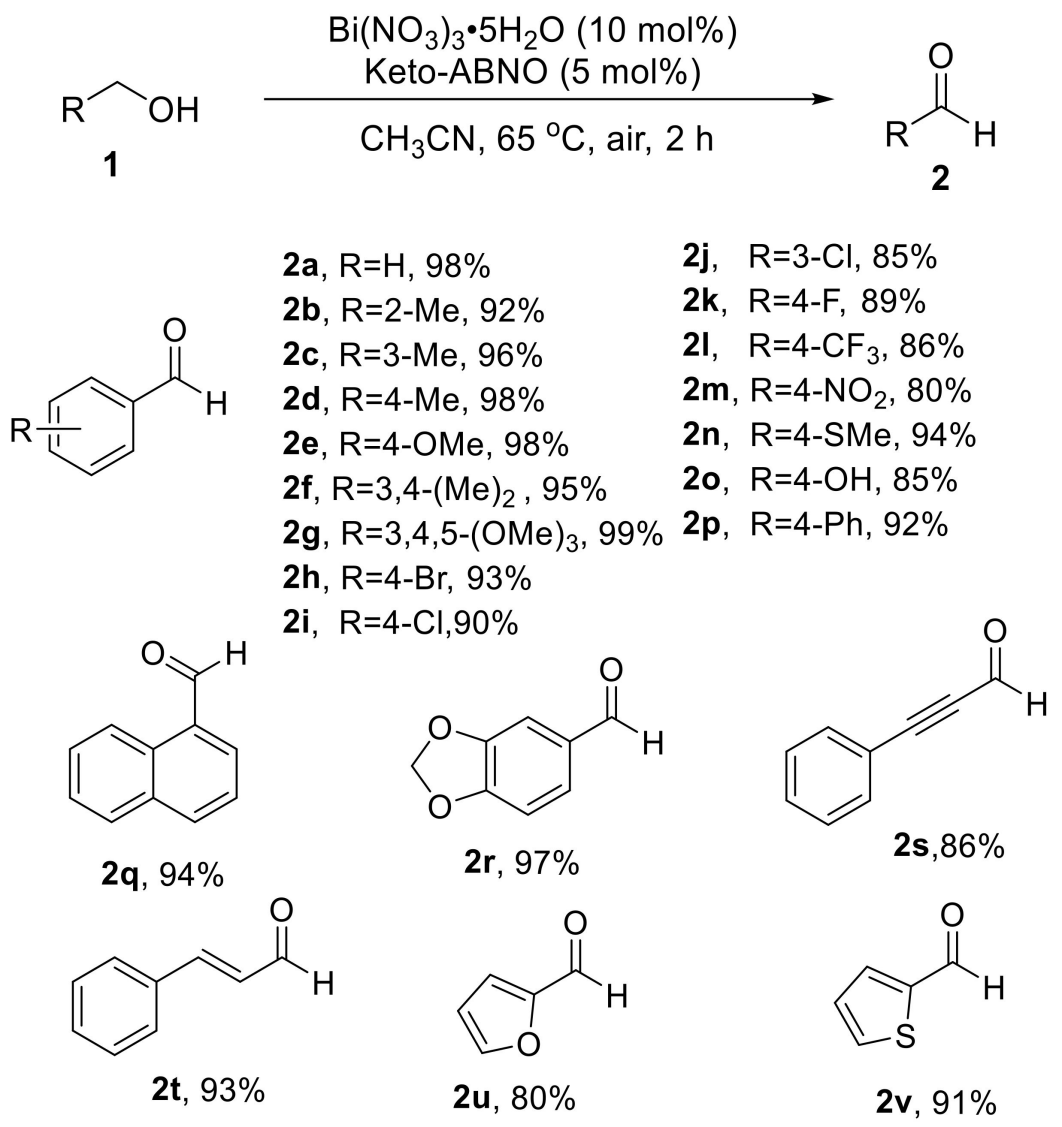
Aerobic oxidation of primary alcohols to aldehydes. Reaction condition: **1a** (1 mmol), Bi(NO_3_)_3_·5H_2_O (10 mol%), Keto-ABNO (5 mol%), CH_3_CN (2 mL) in a 10 mL Schlenk tube, at 65 °C for 2 h, under air-balloon conditions. Isolated yield.

With the optimized reaction conditions defined above, the substrate scope of this reaction was explored, and the representative results are summarized in Scheme 2. A wide range of functionalized primary benzylic alcohols was smoothly transformed to the desired products, with a good-to-excellent yield. It should be observed that electron-donating substituents, such as -CH_3_ and -OCH_3_ on the phenyl groups, could accelerate the reaction, and the substrates were almost entirely oxidized to corresponding aldehydes (**2b**–**2g**). Moreover, the disubstituted and trisubstituted benzylic alcohols still showed excellent conversions in this transformation (**2f** and **2g**). As expected, the benzyl alcohols bearing electron-withdrawing groups, such as -CF_3_, -NO_2_, -F, -Cl, and-Br afforded relatively low product yields (**2h**–**2m**). It is worth noting that the 4-hydroxybenzaldehyde and 4-methylsulfanylbenzaldehyde were obtained at 94% and 85% yields, respectively (**2n** and **2o**). When the 4-phenylbenzyl alcohol, 1-naphthalenemethanol, and piperonyl alcohol were subjected to the standard reaction conditions (**2p**–**2r**), they delivered the corresponding aldehydes with excellent yields (92–97%). The unsaturated aromatic primary alcohols, such as cinnamyl alcohol and 3-phenylprop-2-yn-1-ol, also provided the corresponding products with excellent yields. The heteroarylmethanols derived from furan and thiofuran were all oxidized smoothly to their corresponding aldehydes (**2u** and **2v**).

**Scheme 3:**
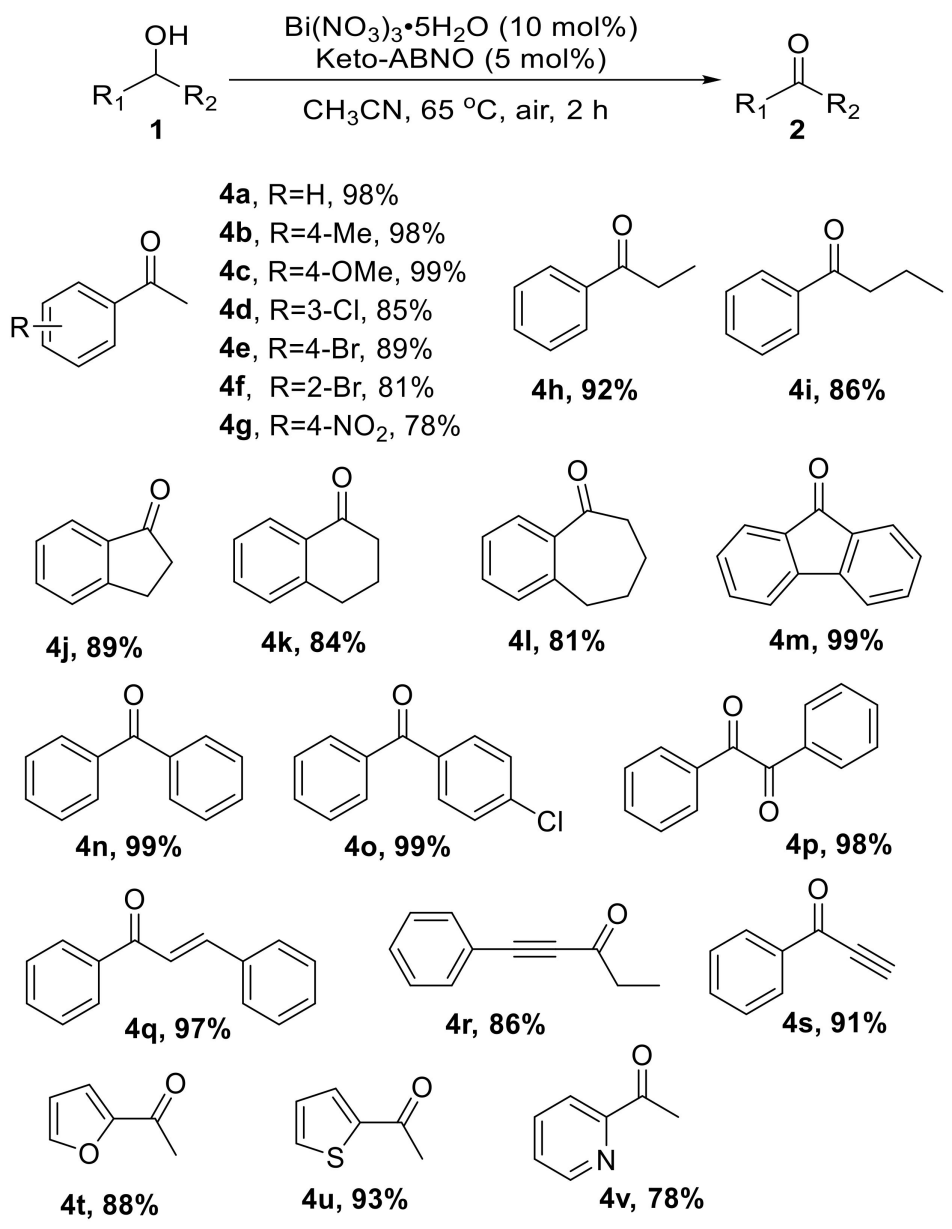
Aerobic oxidation reaction of secondary alcohols to ketones. Reaction conditions: **1a** (1 mmol), Bi(NO_3_)_3_ (10 mol%), Keto-ABNO (5 mol%), CH_3_CN (2 mL) in a 10 mL Schlenk tube, at 65 °C for 2 h, under air-balloon conditions. Bi(NO_3_)_3_ = Bi(NO_3_)_3_·5H_2_O unless otherwise noted. Isolated yield.

After successfully investigating the different primary alcohols, we proceeded to evaluate the general applicability of this catalytic system for various secondary alcohols (Scheme 3). Fortunately, the secondary benzylic alcohols bearing either electron-rich or electron-deficient functional groups provide the corresponding ketones with excellent yields (**4a**–**4g**). Interestingly, the oxidation of the sterically hindered secondary alcohols had little effect on the reaction (**4h**–**4l**). The 9-fluorenol, diphenylic alcohols, and benzoin were almost quantitatively converted into corresponding ketones (**4m**–**4p**). The unsaturated secondary alcohols bearing carbon–carbon double bonds or triple bonds were not affected by the present aerobic oxidation conditions (**4q**–**4s**). Finally, the heteroaromatic secondary alcohols, such as 1-(pyridin-2-yl)ethan-1-ol, 1-(thiophen-2-yl)ethan-1-ol, and 1-(furan-2-yl)ethan-1-ol were also transformed into the corresponding products.

**Scheme 4:**
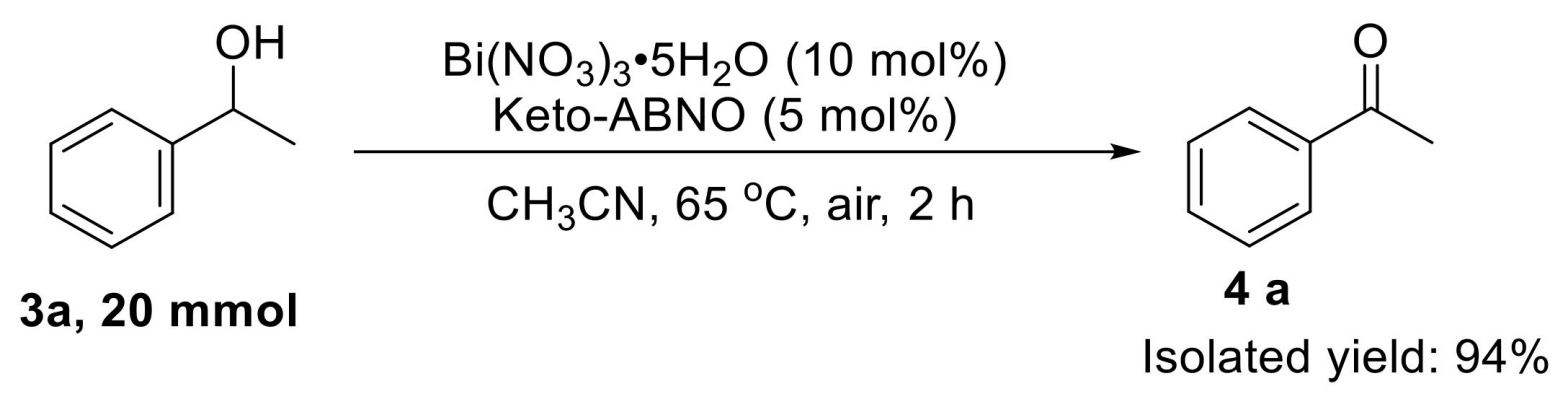
Transformation on the large-scale.

To further demonstrate the gram-scale practicality of this catalytic system, the oxidation of 1-phenylethanol (20 mmol) was carried out under optimized conditions to give 2.294 g of acetophenone with an isolated yield of 94% (Scheme 4). These results suggest that this reaction system exhibits excellent catalytic ability for the aerobic oxidation of alcohols and could be amenable to scale-up.

**Scheme 5:**
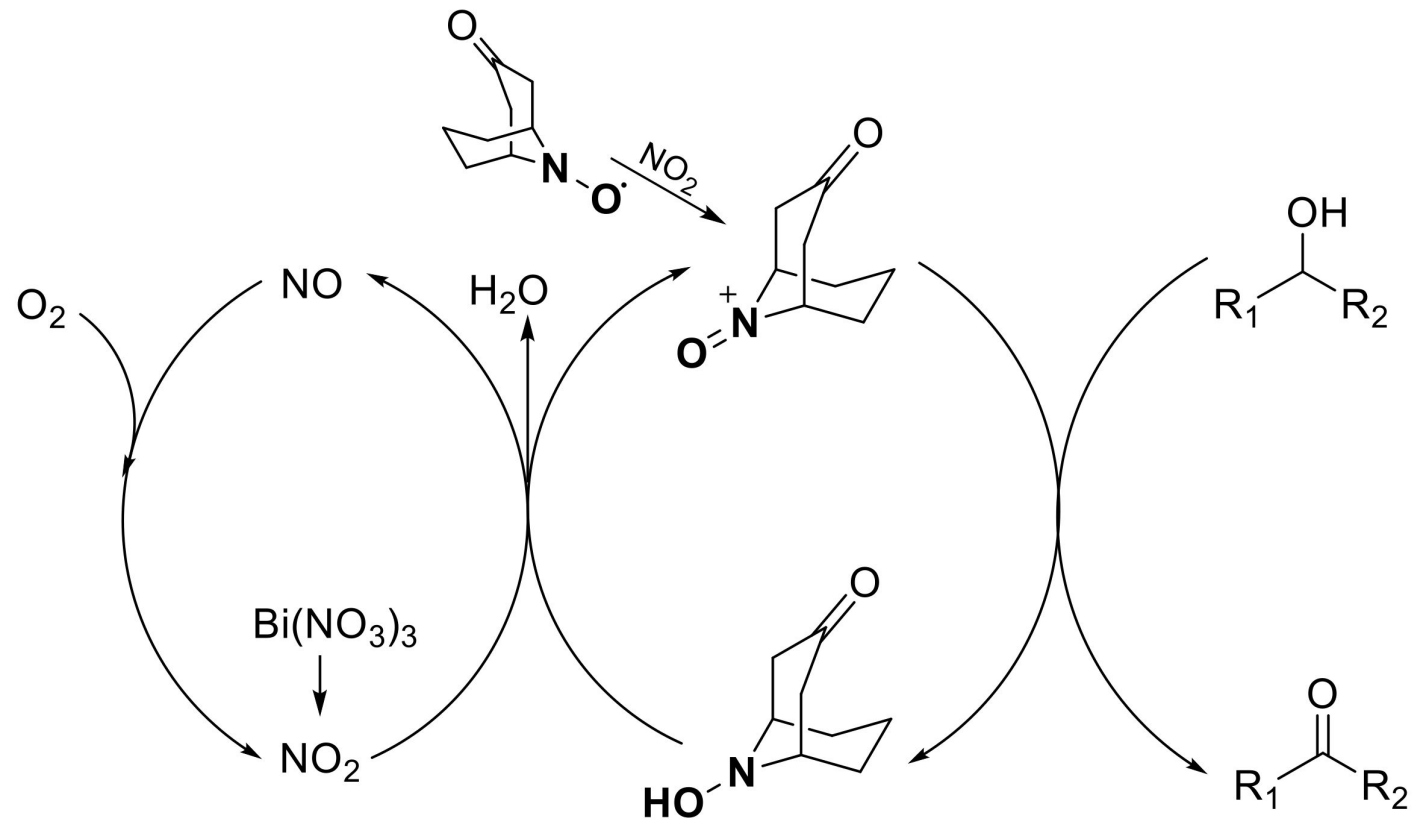
Proposed reaction mechanism.

According to the results above and the literature [40,41,42,43,44,45], a reasonable mechanism for the aerobic oxidation of alcohols catalyzed by Bi(NO_3_)_3_/Keto-ABNO is proposed in Scheme 5. Under a certain reaction temperature, bismuth nitrate released NO_2_, which was of great significance in the oxidation of Keto-ABNO to Keto-ABNO^+^. Next, the Keto-ABNO^+^ oxidized the alcohols to carbonyl compounds, and was itself reduced to Keto-ABNOH at the same time. Subsequently, the Keto-ABNOH was also oxidized to Keto-ABNO^+^ by NO_2_, which turned into NO immediately. Finally, the NO was easily reoxidized to NO_2_ by molecular oxygen.

## 3. Experimental Section

### 3.1. General Information

All reagents were purchased from commercial suppliers without further purification (Energy Chemical, Shanghai, China). Experiments were monitored by thin-layer chromatography (TLC), and the TLC was performed on pre-coated silica-gel plates. The ^1^H- and ^13^C-NMR spectra were recorded on a Bruker Avance III 500 MHz spectrometer (Bruker, Karlsruhe, Germany) with CDCl_3_ as the solvent and tetramethylsilane (TMS) as an internal standard at room temperature. Chemical shifts are given in δ relative to TMS, and the coupling constant *J* values are given in Hertz (Hz). Column chromatography was carried out over 200–300 mesh silica gel. High-resolution mass spectra (HRMS) were obtained on an Agilent mass spectrometer (Agilent, Palo Alto, CA, USA) using ESI-TOF (electrospray ionization-time of flight).

### 3.2. General Procedure

Alcohols (1 mmol), Keto-ABNO (5 mol%, 0.05 mmol), Bi(NO_3_)_3_·5H_2_O (10 mol%, 0.1 mmol), and 2 mL CH_3_CN were added to a 10 mL Schlenk tube equipped with a magnetic stirrer. Next, the mixture was stirred at 65 °C for 2 h under air-balloon conditions. The progress of the reaction was monitored by TLC. After completion of the reaction, the solution was diluted with ethyl acetate and washed with water, before the organic layer was separated and dried over anhydrous MgSO_4_. The solvent was concentrated under reduced pressure and the crude product was purified by column chromatography on silica gel using PE/EtOAc as eluent to afford the pure products. All products were characterized by ^1^H-NMR and ^13^C-NMR (Supplementary Materials).

## 4. Conclusions

In summary, we described a highly efficient, simple, and practical Bi(NO_3_)_3_·5H_2_O/Keto-ABNO-catalyzed oxidation system for the aerobic oxidation of alcohols to aldehydes/ketones with air as an eco-friendly terminal oxidant. This catalyst system has a broad functional group tolerance, and can be used to oxidize a variety of primary and secondary alcohols to corresponding aldehydes and ketones in moderate to high yields. Moreover, this methodology avoids using a ligand and base. The utilization of catalytic amounts of Bi(NO_3_)_3_·5H_2_O and Keto-ABNO was successful in a scaled-up reaction, which demonstrated its high potential for use on an industrial scale. Further studies of the reaction mechanism, as well as the applications of this method, are in progress.

## Figures and Tables

**Table 1:** Optimization of reaction conditions ^a^.

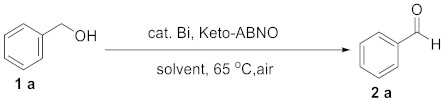					
**Entry**	**Bi-Catalyst (mol%)**	**Nitroxyl Radical (mol%)**	**Solvent**	**Time (h)**	**Yield (%) ^b^**
1	-	TEMPO (3)	CH_3_CN	3	trace
2	Bi(NO_3_)_3_ (5)	-	CH_3_CN	3	trace
3	Bi_2_(SO_4_)_3_ (5)	TEMPO (3)	CH_3_CN	3	10
4	Bi_2_O_3_ (5)	TEMPO (3)	CH_3_CN	3	23
5	BiBr_3_ (5)	TEMPO (3)	CH_3_CN	3	trace
6	Bi(OTf)_3_ (5)	TEMPO (3)	CH_3_CN	3	15
7	Bi(NO_3_)_3_ (5)	TEMPO (3)	CH_3_CN	3	89
8	Bi(NO_3_)_3_ (5)	4-HO-TEMPO (3)	CH_3_CN	3	86
9 ^c^	Bi(NO_3_)_3_ (5)	ACT (3)	CH_3_CN	3	92
10	Bi(NO_3_)_3_ (5)	Keto-ABNO (3)	CH_3_CN	3	95
11	Bi(NO_3_)_3_ (10)	Keto-ABNO (5)	CH_3_CN	2	98
12	Bi(NO_3_)_3_ (15)	Keto-ABNO (10)	CH_3_CN	2	98
13	Bi(NO_3_)_3_ (10)	Keto-ABNO (5)	CH_3_CN	1	93
14	Bi(NO_3_)_3_ (10)	Keto-ABNO (5)	Ethanol	2	86
15	Bi(NO_3_)_3_ (10)	Keto-ABNO (5)	DMSO	2	75
16	Bi(NO_3_)_3_ (10)	Keto-ABNO (5)	DCE	2	94
17	Bi(NO_3_)_3_ (10)	Keto-ABNO (5)	H_2_O	2	45
18 ^d^	Bi(NO_3_)_3_ (10)	Keto-ABNO (5)	CH_3_CN	2	98
19 ^e^	Bi(NO_3_)_3_ (10)	Keto-ABNO (5)	CH_3_CN	2	15

^a^ Reaction condition: **1a** (1 mmol), Bi catalyst, nitroxyl radical, solvent (2 mL) in a 10 mL Schlenk tube, at 65 °C, under air-balloon conditions. Bi(NO_3_)_3_ = Bi(NO_3_)_3_·5H_2_O, unless otherwise noted. ^b^ Isolated yield. ^c^ ACT = 4-Acetamido-TEMPO. ^d^ Under O_2_ atmosphere. ^e^ Under N_2_ atmosphere.

## Data Availability

The data presented in this study are available in this article.

## Supplementary Materials

The following supporting information can be downloaded at: https://www.mdpi.com/article/10.3390/molecules27123727/s1, ^1^H-NMR and ^13^C-NMR spectra of compounds **2a**–**2v** and **4a**–**4v**. Reference [46] is cited in the Supplementary Materials.

## Sample Availability

Samples of the compounds are available from the authors.
