# Supplementary material for: A Highly Efficient Bismuth Nitrate/Keto-ABNO Catalyst System for Aerobic Oxidation of Alcohols to Carbonyl Compounds under Mild Conditions
Source: Molecules. 2022 Jun 9;27(12):3727. doi: 10.3390/molecules27123727 (PMC9230008; doi:10.3390/molecules27123727)

Supplementary Materials

A Highly Efficient Bismuth Nitrate/Keto-ABNO Catalyst System for Aerobic Oxidation of Alcohols to Carbonyl Compounds under Mild Conditions

Yongke Hu [1,*], Lei Chen [2], Gulou Shen [1], Jin Li [1], Shaozhong Li [1], Huaju Li [1] and Yanxing Li [1]

[1] National & Local Joint Engineering Research Center for Mineral Salt Deep Utilization, Huaiyin Institute of Technology, Huaian 223003, China; lsheng@hyit.edu.cn (G.S.); lijin96998@hyit.edu.cn (J.L.); lsz114@hyit.edu.cn (S.L.); huajuli@hyit.edu.cn (H.L.); liyanxing@hyit.edu.cn (Y.L.)

[2] Zhongbei College, Nanjing Normal University, Zhenjiang 212300, China; zb_chenlei@163.com

* Correspondence: huyongke@hyit.edu.cn

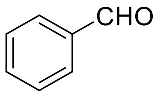

Benzaldehyde (2 a) [1]: ^{1}H NMR (500 MHz, $CDCl_3$) δ 10.03 (s, 1H), 7.89 (d, *J* = 6.9 Hz, 2H), 7.64 (t, *J* = 7.4 Hz, 1H), 7.54 (t, *J* = 7.6 Hz, 2H). ^{13}C NMR (125 MHz, $CDCl_3$) δ 192.33, 136.29, 134.42, 129.67, 128.96.

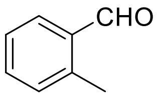

2-Methylbenzaldehyde (2b) [1]: ^{1}H NMR (500 MHz, $CDCl_3$) δ 10.26 (s, 1H), 7.79 (d, *J* = 7.6 Hz, 1H), 7.47 (t, *J* = 7.5 Hz, 1H), 7.35 (t, *J* = 7.5 Hz, 1H), 7.25 (d, *J* = 7.5 Hz, 1H), 2.66 (s, 3H). ^{13}C NMR (125 MHz, $CDCl_3$) δ 192.56, 140.36, 133.92, 133.42, 131.82, 131.55, 126.10, 77.47, 77.21, 76.96, 19.35.

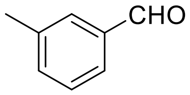

3-Methylbenzaldehyde (2c) [2]: ^{1}H NMR (500 MHz, $CDCl_3$) δ 10.00 (s, 1H), 7.69 (d, *J* = 7.6 Hz, 2H), 7.44 (m, 2H), 2.44 (s, 3H). ^{13}C NMR (125 MHz, $CDCl_3$) δ 192.32, 138.67, 136.26, 135.04, 129.77, 128.64, 126.98, 20.95.

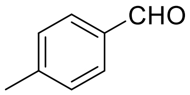

4-Methylbenzaldehyde (2d) [1]: ^{1}H NMR (500 MHz, $CDCl_3$) δ 9.96 (s, 1H), 7.78 (d, *J* = 8.1 Hz, 2H), 7.33 (d, *J* = 8.0 Hz, 2H), 2.44 (s, 3H). ^{13}C NMR (125 MHz, $CDCl_3$) δ 191.71, 145.27, 133.94, 129.56, 129.43, 21.56.

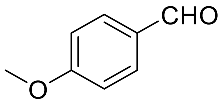

4-Methoxybenzaldehyde (2e) [1]: ^{1}H NMR (500 MHz, $CDCl_3$) δ 9.87 (s, 1H), 7.82 (d, *J* = 8.8 Hz, 2H), 6.99 (d, *J* = 8.8 Hz, 2H), 3.87 (s, 3H). ^{13}C NMR (125 MHz, $CDCl_3$) δ 190.52, 164.35, 131.70, 129.69, 114.05, 55.30.

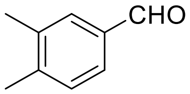

3,4-Dimethylbenzaldehyde (2f) [3]: ^{1}H NMR (500 MHz, $CDCl_3$) δ 9.93 (s, 1H), 7.64 (s, 1H), 7.61 (d, *J* = 7.7 Hz, 1H), 7.28 (d, *J* = 7.7 Hz, 1H), 2.34 (s, 3H), 2.33 (s, 3H). ^{13}C NMR (125 MHz, $CDCl_3$) δ 191.97, 144.04, 137.22, 134.35, 130.29, 129.97, 127.46, 19.97, 19.36.

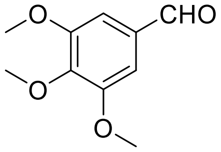

3,4,5-Trimethoxybenzaldehyde (2g) [3]: ^{1}H NMR (500 MHz, $CDCl_3$) δ 9.87 (s, 1H), 7.13 (s, 2H), 3.94 (s, 3H), 3.93 (s, 6H). ^{13}C NMR (125 MHz, $CDCl_3$) δ 190.76, 153.34, 143.30, 131.43, 106.43, 60.66, 55.97.

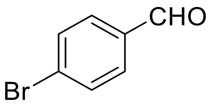

4-Bromobenzaldehyde (2h) [3]: ^{1}H NMR (500 MHz, $CDCl_3$) δ 9.98 (s, 1H), 7.75 (d, *J* = 8.4 Hz, 2H), 7.69 (d, *J* = 8.4 Hz, 2H).^{13}C NMR (125 MHz, $CDCl_3$) δ 190.78, 134.82, 132.17, 130.70, 129.50.

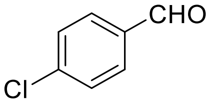

4-Chlorobenzaldehyde (2i) [1]: ^{1}H NMR (500 MHz, $CDCl_3$) δ 9.99 (s, 1H), 7.83 (d, *J* = 8.4 Hz, 2H), 7.52 (d, *J* = 8.4 Hz, 2H). ^{13}C NMR (125 MHz, $CDCl_3$) δ 190.59, 140.70, 134.47, 130.65, 129.21.

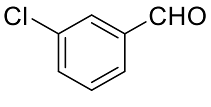

3-Chlorobenzaldehyde (2j) [3]: ^{1}H NMR (500 MHz, $CDCl_3$) δ 9.98 (s, 1H), 7.85 (s, 1H), 7.77 (d, *J* = 7.6 Hz, 1H), 7.60 (d, *J* = 8.9 Hz, 1H), 7.49 (t, *J* = 7.8 Hz, 1H). ^{13}C NMR (125 MHz, $CDCl_3$) δ 190.59, 137.57, 135.20, 134.15, 130.15, 129.04, 127.74.

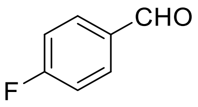

4-Fluorobenzaldehyde (2k) [1]: ^{1}H NMR (500 MHz, $CDCl_3$) δ 9.97 (s, 1H), 7.92 (dd, *J* = 8.5, 5.5 Hz, 2H), 7.22 (t, *J* = 8.5 Hz, 2H). ^{13}C NMR (126 MHz, $CDCl_3$) δ 190.25, 166.29 (d, *J* =257.2), 132.75, 132.0 (d, *J* = 9.0 Hz), 116.13 (d, *J* = 22.1 Hz).

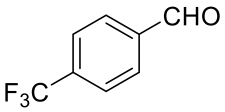

4-(Trifluoromethyl)benzaldehyde (2l) [3]: ^{1}H NMR (500 MHz, $CDCl_3$) δ 10.12 (s, 1H), 8.03 (d, *J* = 8.0 Hz, 2H), 7.83 (d, *J* = 8.1 Hz, 2H). ^{13}C NMR (125 MHz, $CDCl_3$) δ 190.90, 138.47, 135.62 (q, *J* = 32.4 Hz), 129.72, 125.91, 123.2(q, *J* = 274.7 Hz).

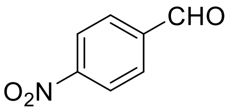

4-Nitrobenzaldehyde (2m) [3]: ^{1}H NMR (500 MHz, $CDCl_3$) δ 10.17 (s, 1H), 8.40 (d, *J* = 8.7 Hz, 2H), 8.09 (d, *J* = 8.8 Hz, 2H). ^{13}C NMR (125 MHz, $CDCl_3$) δ 190.02, 150.88, 139.79, 130.23, 124.06.

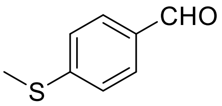

4-(Methylthio)benzaldehyde (2n) [1]: ^{1}H NMR (500 MHz, $CDCl_3$) δ 9.91 (s, 1H), 7.76 (d, *J* = 8.4 Hz, 2H), 7.32 (d, *J* = 8.4 Hz, 2H), 2.53 (s, 3H). ^{13}C NMR (125 MHz, $CDCl_3$) δ 190.91, 147.64, 132.62, 129.67, 124.86, 14.34.

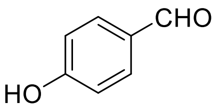

4-Hydroxybenzaldehyde (2o) [3]: ^{1}H NMR (500 MHz, $CDCl_3$) δ 9.84 (s, 1H), 7.83 (d, *J* = 8.6 Hz, 2H), 7.38 (s, 1H), 7.01 (d, *J* = 8.6 Hz, 2H). ^{13}C NMR (125 MHz, $CDCl_3$) δ 191.66, 162.12, 132.52, 129.13,

115.92, 77.03, 76.77, 76.52.

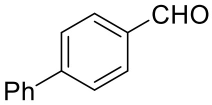

4-Phenylbenzaldehyde (2p) [1]: ^{1}H NMR (500 MHz, $CDCl_3$) δ 10.06 (s, 1H), 7.96 (d, *J* = 8.1 Hz, 2H), 7.76 (d, *J* = 8.2 Hz, 2H), 7.64 (d, *J* = 7.6 Hz, 2H), 7.49 (t, *J* = 7.6 Hz, 2H), 7.43 (t, *J* = 7.3 Hz, 1H). ^{13}C NMR (125 MHz, $CDCl_3$) δ 191.68, 146.88, 139.42, 134.97, 130.03, 128.80, 128.27, 127.58, 127.41, 127.12.

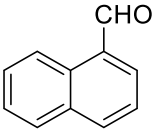

1-Naphthalenecarboxaldehyde (2q) [1]: ^{1}H NMR (500 MHz, $CDCl_3$) δ 10.36 (s, 1H), 9.23 (d, *J* = 8.6 Hz, 1H), 8.05 (d, *J* = 8.2 Hz, 1H), 7.93 (d, *J* = 7.0 Hz, 1H), 7.88 (d, *J* = 8.2 Hz, 1H), 7.66 (t, *J* = 7.1 Hz, 1H), 7.57 (dd, *J* = 15.7, 8.1 Hz, 2H). ^{13}C NMR (125 MHz, $CDCl_3$) δ 193.30, 136.44, 135.05, 133.48, 131.15, 130.28, 128.83, 128.25, 126.73, 124.64.

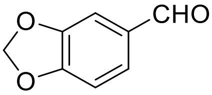

Piperonyl alcohol (2r) [3]: ^{1}H NMR (500 MHz, $CDCl_3$) δ 9.81 (s, 1H), 7.42 (dd, *J* = 7.9, 1.5 Hz, 1H), 7.34 (d, *J* = 1.5 Hz, 1H), 6.94 (d, *J* = 7.9 Hz, 1H), 6.08 (s, 2H). ^{13}C NMR (125 MHz, $CDCl_3$) δ 190.05, 152.85, 148.46, 131.63, 128.43, 108.11, 106.65, 101.87.

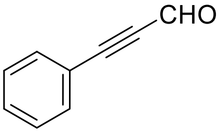

Phenylpropiolaldehyde (2s) [1]: ^{1}H NMR (500 MHz, $CDCl_3$) δ 9.43 (s, 1H), 7.61 (d, *J* = 7.1 Hz, 2H), 7.49 (t, *J* = 7.5 Hz, 1H), 7.41 (t, *J* = 7.6 Hz, 2H). ^{13}C NMR (125 MHz, $CDCl_3$) δ 176.54, 133.04, 131.05, 128.49, 119.18, 94.88, 88.18.

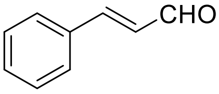

Cinnamaldehyde (2t) [2]: ^{1}H NMR (500 MHz, $CDCl_3$) δ 9.73 (d, *J* = 7.7 Hz, 1H), 7.59 (m, 2H), 7.53 – 7.42 (m, 4H), 6.74 (dd, *J* = 15.9, 7.7 Hz, 1H). ^{13}C NMR (125 MHz, $CDCl_3$) δ 193.51, 152.59, 133.77, 131.07, 128.90, 128.37, 128.29.

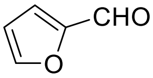

2-Furancarboxaldehyde (2u) [2]: ^{1}H NMR (500 MHz, $CDCl_3$) δ 9.69 (s, 1H), 7.72 (s, 1H), 7.28 (d, *J* = 3.5 Hz, 1H), 6.63 (dd, *J* = 3.5, 1.4 Hz, 1H). ^{13}C NMR (125 MHz, $CDCl_3$) δ 177.69, 152.78, 147.92, 120.76, 112.39, 77.55, 77.29, 77.04.

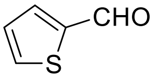

2-Thiophenecarboxaldehyde (2v) [4]: ^{1}H NMR (500 MHz, $CDCl_3$) δ 9.94 (s, 1H), 7.79 (d, *J* = 3.7 Hz, 1H), 7.77 (d, *J* = 4.9 Hz, 1H), 7.23 – 7.20 (m, 1H). ^{13}C NMR (125 MHz, $CDCl_3$) δ 182.77, 143.79,

136.11, 134.91, 128.10.

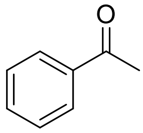

Acetophenone (4a) [4]**:** ^{1}HNMR (500 MHz, $CDCl_3$) δ 7.96 (d, *J* = 8.1 Hz, 2H), 7.57 (t, *J* = 7.4 Hz, 1H), 7.47 (t, *J* = 7.7 Hz, 2H), 2.61 (s, 3H).^{13}C NMR (125 MHz, $CDCl_3$) δ 197.91, 136.86, 132.85, 128.39, 128.18, 26.36.

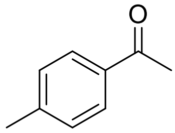

4-Methylacetophenone (4b) [5]**:** ^{1}H NMR (500 MHz, $CDCl_3$) δ 7.85 (d, *J* = 8.2 Hz, 2H), 7.25 (d, *J* = 7.9 Hz, 2H), 2.57 (s, 3H), 2.41 (s, 3H). ^{13}C NMR (125 MHz, $CDCl_3$) δ 197.61, 143.61, 134.46, 128.98, 128.18, 26.25, 21.36.

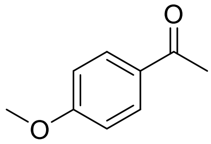

1-(4-Methoxyphenyl)ethenone (4c) [4]**:** ^{1}H NMR (500 MHz, $CDCl_3$) δ 7.94 (d, *J* = 8.1 Hz, 2H), 6.93 (d, *J* = 8.1 Hz, 2H), 3.87 (s, 3H), 2.55 (s, 3H). ^{13}C NMR (125 MHz, $CDCl_3$) δ 196.50, 163.22, 130.31, 130.08, 113.41, 55.19, 26.05.

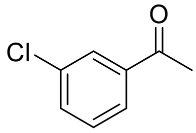

1-(3-Chlorophenyl)ethanone (4d) [5]**:** ^{1}H NMR (500 MHz, $CDCl_3$) δ 7.93 (s, 1H), 7.83 (d, *J* = 7.7 Hz, 1H), 7.54 (d, *J* = 7.9 Hz, 1H), 7.41 (t, *J* = 7.8 Hz, 1H), 2.60 (s, 3H). ^{13}C NMR (125 MHz, $CDCl_3$) δ 196.45, 138.36, 134.67, 132.77, 129.67, 128.15, 126.14, 26.37.

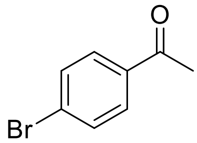

1-(4-Bromophenyl)ethanone (4e) [5]**:** ^{1}H NMR (500 MHz, $CDCl_3$) δ 7.82 (d, *J* = 8.4 Hz, 2H), 7.61 (d, *J* = 8.4 Hz, 2H), 2.59 (s, 3H). ^{13}C NMR (125 MHz, $CDCl_3$) δ 196.75, 135.58, 131.64, 129.58, 128.04, 26.26.

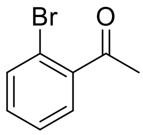

1-(2-Bromophenyl)ethanone (4f) [5]**:**^{1}H NMR (500 MHz, $CDCl_3$) δ 7.60 (d, *J* = 8.0 Hz, 1H), 7.45 (d, *J* = 7.6 Hz, 1H), 7.36 (t, *J* = 7.5 Hz, 1H), 7.28 (t, *J* = 7.6 Hz, 1H), 2.62 (s, 3H).^{13}C NMR (125 MHz, $CDCl_3$) δ 201.07, 141.19, 133.57, 131.54, 128.65, 127.19, 118.62, 30.06.

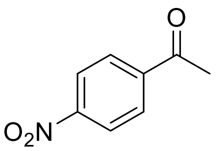

1-(4-Nitrophenyl)ethanone (4g) [5]: ^{1}H NMR (500 MHz, $CDCl_3$) δ 8.32 (d, *J* = 7.7 Hz, 2H), 8.13 (d, *J* = 7.6 Hz, 2H), 2.70 (s, 3H). ^{13}C NMR (125 MHz, $CDCl_3$) δ 196.05, 150.10, 141.13, 129.05, 123.59, 26.71.

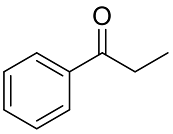

Propiophenone (4h) [4]: ^{1}H NMR (500 MHz, $CDCl_3$) δ 7.96 (d, *J* = 8.5 Hz, 2H), 7.55 (t, *J* = 7.4 Hz, 1H), 7.45 (t, *J* = 7.6 Hz, 2H), 3.00 (q, *J* = 7.2 Hz, 2H), 1.23 (t, *J* = 7.2 Hz, 3H). ^{13}C NMR (125 MHz, $CDCl_3$) δ 200.57, 136.67, 132.61, 128.29, 127.71, 31.51, 7.97.

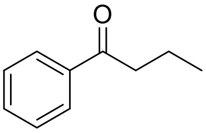

***n*-Butyrophenone(4i) [4]:** ^{1}H NMR (500 MHz, $CDCl_3$) δ 7.96 (d, *J* = 7.7 Hz, 2H), 7.55 (t, *J* = 7.3 Hz, 1H), 7.45 (t, *J* = 7.5 Hz, 2H), 2.95 (t, *J* = 7.3 Hz, 2H), 1.83 – 1.70 (m, 2H), 1.01 (t, *J* = 7.4 Hz, 3H). ^{13}C NMR (125 MHz, $CDCl_3$) δ 200.17, 136.86, 132.60, 128.28, 127.77, 40.25, 17.51, 13.63.

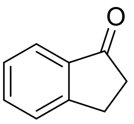

1-Indanone (4j) [4]: ^{1}H NMR (500 MHz, $CDCl_3$) δ 7.75 (d, *J* = 7.7 Hz, 1H), 7.58 (t, *J* = 7.4 Hz, 1H), 7.48 (d, *J* = 7.7 Hz, 1H), 7.36 (t, *J* = 7.8 Hz, 1H), 3.4 (t, *J* = 6.0 Hz, 2H), 2.68 (t, *J* = 6.2 Hz, 2H). ^{13}C NMR (125 MHz, $CDCl_3$) δ 206.82, 154.91, 136.81, 134.34, 127.01, 126.44, 123.43, 35.95, 25.54.

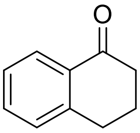

3,4-Dihydronaphthalen-1(2H)-one (4k) [4]: ^{1}H NMR (500 MHz, $CDCl_3$) δ 8.01 (d, *J* = 7.8 Hz, 1H), 7.44 (t, *J* = 7.4 Hz, 1H), 7.28 (t, *J* = 7.4 Hz, 1H), 7.23 (d, *J* = 7.6 Hz, 1H), 2.94 (t, *J* = 6.0 Hz, 2H), 2.63 (t, *J* = 6.4 Hz, 2H), 2.15 – 2.08 (m, 2H). ^{13}C NMR (125 MHz, $CDCl_3$) δ 198.06, 144.23, 133.12, 132.34, 128.52, 126.86, 126.34, 38.89, 29.42, 23.02.

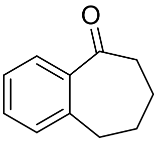

6,7,8,9-tetrahydrobenzo[7]annulen-5-one (4l) [4]: ^{1}H NMR (500 MHz, $CDCl_3$) δ 7.72 (dd, *J* = 7.7, 1.3 Hz, 1H), 7.42 (td, *J* = 7.5, 1.4 Hz, 1H), 7.30 (t, *J* = 7.5 Hz, 1H), 7.20 (d, *J* = 7.5 Hz, 1H), 2.93 (t, *J* = 6.5 Hz,2H), 2.76 – 2.71 (m, 2H), 1.92 – 1.86 (m, 2H), 1.84 – 1.78 (m, 2H). ^{13}C NMR (125 MHz, $CDCl_3$) δ 205.72, 141.03, 138.54, 131.90, 129.42, 128.25, 126.33, 40.56, 32.20, 24.96, 20.65.

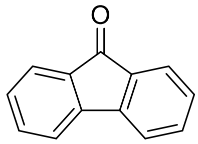

9-Fluorenone (4m) [5]**:** ^{1}H NMR (500 MHz, $CDCl_3$) δ 7.65 (d, J = 7.4 Hz, 2H), 7.51 (d, J = 7.2 Hz, 2H), 7.47 (t, J = 7.3 Hz, 2H), 7.28 (t, J = 7.3 Hz, 2H). ^{13}C NMR (125 MHz, $CDCl_3$) δ 193.64, 144.18, 134.41, 133.90, 128.81, 124.05, 120.04.

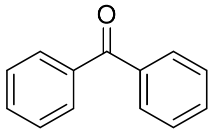

Benzophenone (4n) [4]**:** ^{1}H NMR (500 MHz, $CDCl_3$) δ 7.80 (d, J = 7.0 Hz, 4H), 7.58 (t, J = 7.4 Hz, 2H), 7.48 (t, J = 7.7 Hz, 4H). ^{13}C NMR (125 MHz, $CDCl_3$) δ 196.48, 137.35, 132.18, 129.81, 128.04.

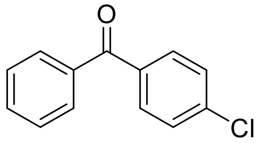

4-Chlorobenzophenone (4o) [5]**:** ^{1}H NMR (500 MHz, $CDCl_3$) δ 7.76 (t, J = 8.3 Hz, 4H), 7.60 (t, J = 7.4 Hz, 1H), 7.50 (d, J = 7.8 Hz, 2H), 7.46 (d, J = 8.5 Hz, 2H). ^{13}C NMR (125 MHz, $CDCl_3$) δ 195.23, 138.64, 136.98, 135.61, 132.40, 131.21, 129.68, 128.38, 128.16.

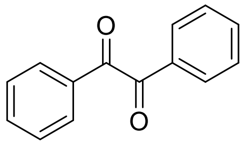

Benzil (4p) [4]**:**^{1}H NMR (500 MHz, $CDCl_3$) δ 7.98 (d, J = 7.2 Hz, 4H), 7.66 (t, J = 7.4 Hz, 2H), 7.52 (t, J = 7.8 Hz, 4H). ^{13}CNMR (125 MHz, $CDCl_3$) δ 194.33, 134.64, 132.75, 129.65, 128.77.

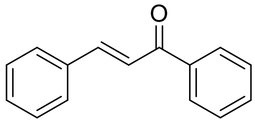

Chalcone (4q) [5]**:** ^{1}H NMR (500 MHz, $CDCl_3$) δ 8.04 (dd, J = 8.5, 1.9 Hz, 2H), 7.82 (d, J = 15.7 Hz, 1H), 7.65 (dd, J = 6.7, 2.9 Hz, 2H), 7.62 – 7.49 (m, 4H), 7.43 (m, 3H). ^{13}C NMR (125 MHz, $CDCl_3$) δ 190.32, 144.61, 137.97, 134.64, 132.55, 130.31, 128.72, 128.39, 128.26, 128.32, 121.85.

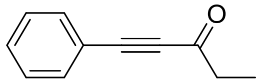

1-phenyl-pent-1-yn-3-one (4r) [3]**:** ^{1}H NMR (500 MHz, $CDCl_3$) δ 7.57 (d, J = 7.0 Hz, 2H), 7.45 (t, J = 7.5 Hz, 1H), 7.38 (t, J = 7.5 Hz, 2H), 2.70 (q, J = 7.4 Hz, 2H), 1.21 (t, J = 7.4 Hz, 3H). ^{13}C NMR (125 MHz, $CDCl_3$) δ 188.29, 132.73, 130.38, 128.36, 119.76, 90.37, 87.37, 38.56, 7.87.

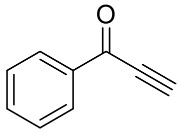

1-phenylprop-2-yn-1-one (4s) [3]**:** ^{1}H NMR (500 MHz, $CDCl_3$) δ 8.17 (d, *J* = 8.0 Hz, 2H), 7.64 (t, *J* = 7.4 Hz, 1H), 7.50 (t, *J* = 7.8 Hz, 2H), 3.44 (s, 1H). ^{13}C NMR (125 MHz, $CDCl_3$) δ 177.13, 135.90, 134.26, 129.45, 128.43, 80.49, 80.02.

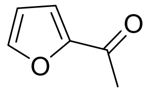

2-Acetylfuran (4t) [5]**:** ^{1}H NMR (500 MHz, $CDCl_3$) δ 7.60 (s, 1H), 7.19 (d, *J* = 3.5 Hz, 1H), 6.54 (m, 1H), 2.48 (s, 3H). ^{13}C NMR (125 MHz, $CDCl_3$) δ 186.52, 152.55, 146.17, 117.00, 111.98 , 25.73 .

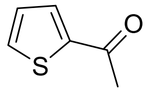

2-Acetylthiophene (4u) [5]**:** ^{1}H NMR (500 MHz, $CDCl_3$) δ 7.68 (d, *J* = 3.8, 1H), 7.62 (d, *J* = 5.0, 1H), 7.11 (m, 1H), 2.55 (s, 3H). ^{13}C NMR (125 MHz, $CDCl_3$) δ 190.49, 144.30, 133.54, 132.25, 127.87, 26.66.

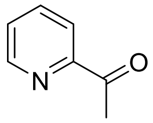

2-Acetylpyridine (4v) [5]**:** ^{1}H NMR (500 MHz, $CDCl_3$) δ 8.67 (d, *J* = 4.8 Hz, 1H), 8.02 (d, *J* = 7.9 Hz, 1H), 7.81 (m, 1H), 7.45 (m, 1H), 2.71 (s, 3H). ^{13}C NMR (125 MHz, $CDCl_3$) δ 199.84, 153.30, 148.70, 136.56, 126.81, 121.37, 25.49 (s).

References

1. Wang, M.; Xu, Z.; Shi, Y.; Cai, F.; Qiu, J.; Yang, G.; Hua, Z.; Chen, T. TEMPO-Functionalized Nanoreactors from Bottlebrush Copolymers for the Selective Oxidation of Alcohols in Water. *J. Org. Chem.* **2021**, *86*, 8027–8035.
2. Wang, L.; Shang, S.; Li, G.; Ren, L.; Lv, Y.; Gao, S. Iron/ABNO-Catalyzed Aerobic Oxidation of Alcohols to Aldehydes and Ketones under Ambient Atmosphere. *J. Org. Chem.* **2016**, *81*, 2189–2193.
3. Hu, Y.; Chen, L; Li, B. $Fe(NO_3)_3$/2,3-dichloro-5,6-dicyano-1,4-benzoquinone (DDQ): An efficient catalyst system for selective oxidation of alcohols under aerobic conditions. *Catal. Commun.* **2018**, *103*, 42–46.
4. Shen, D.; Miao, C.; Xu, D.; Xia, C.; Sun, W. Highly efficient oxidation of secondary alcohols to ketones catalyzed by manganese complexes of N_4 ligands with H_2O_2. *Org. Lett.* **2015**, *17*, 54–57.
5. Hu, Y.; Chen, L.; Li, B. NHPI/*tert*-butyl nitrite: A highly efficient metal-free catalytic system for aerobic oxidation of alcohols to carbonyl compounds using molecular oxygen as the terminal oxidant. *Catal. Commun.* **2016**, *83*, 82–87.

1. ^{1}H NMR and ^{13}C NMR spectra

The ^{1}H and ^{13}C NMR spectra of compounds (2a)

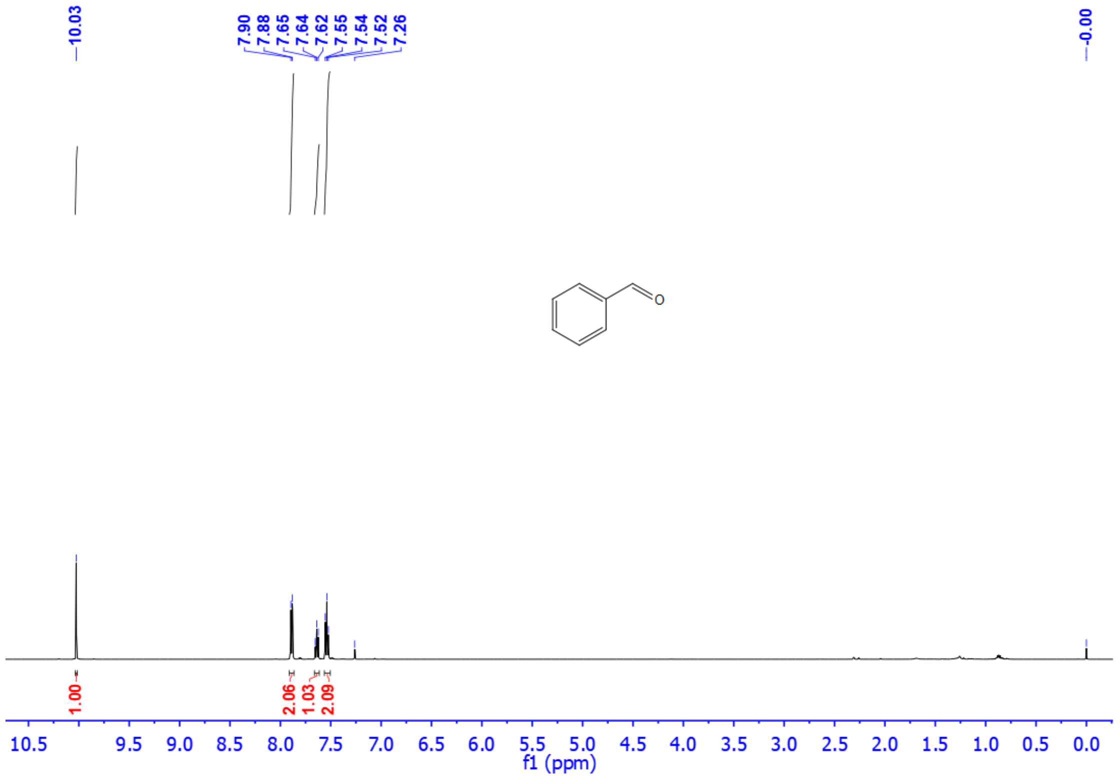

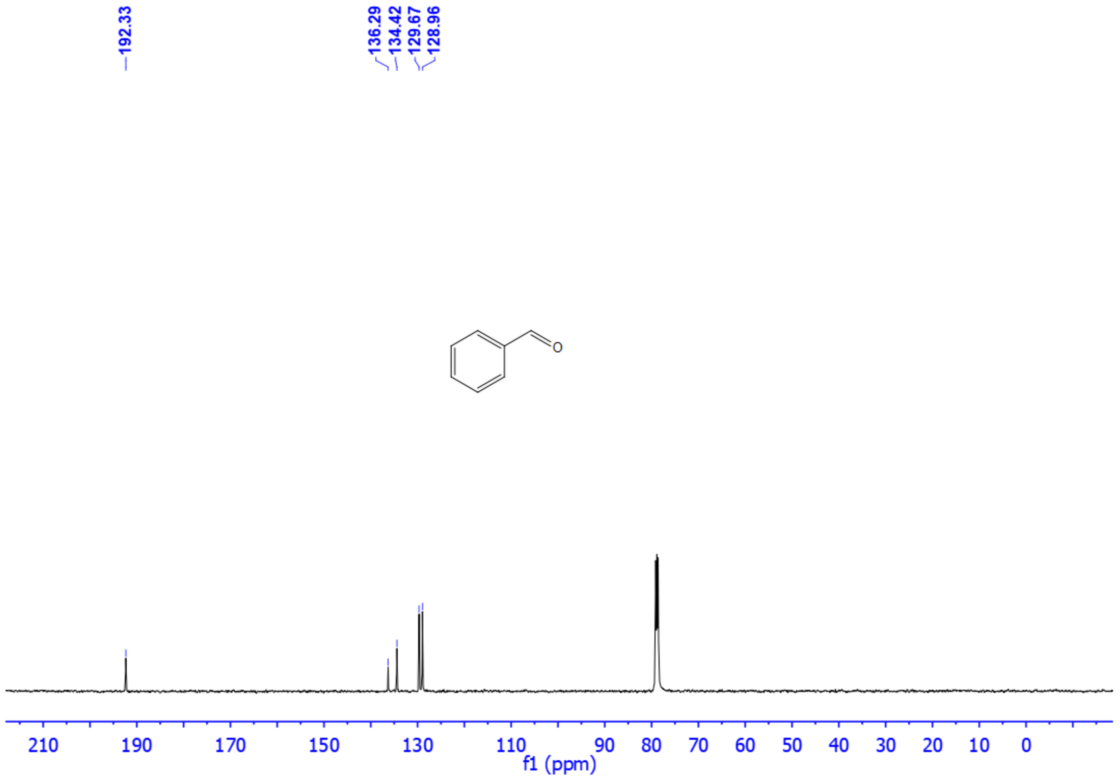

The ^{1}H and ^{13}C NMR spectra of compounds (2b)

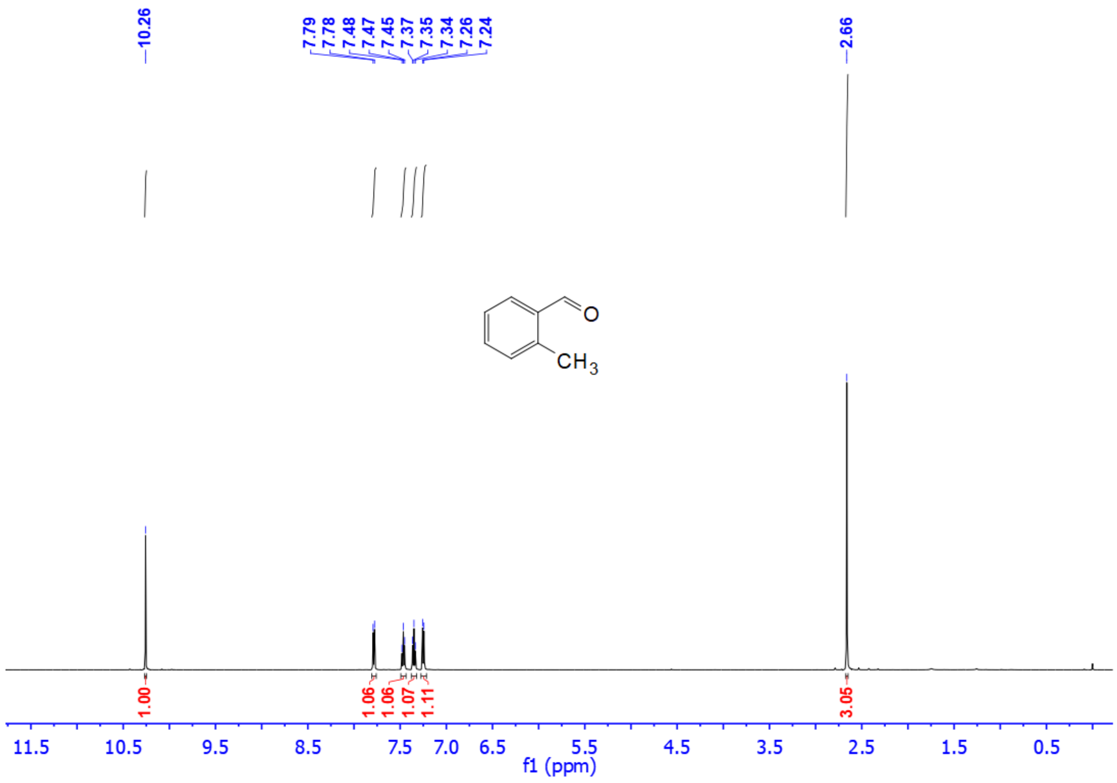

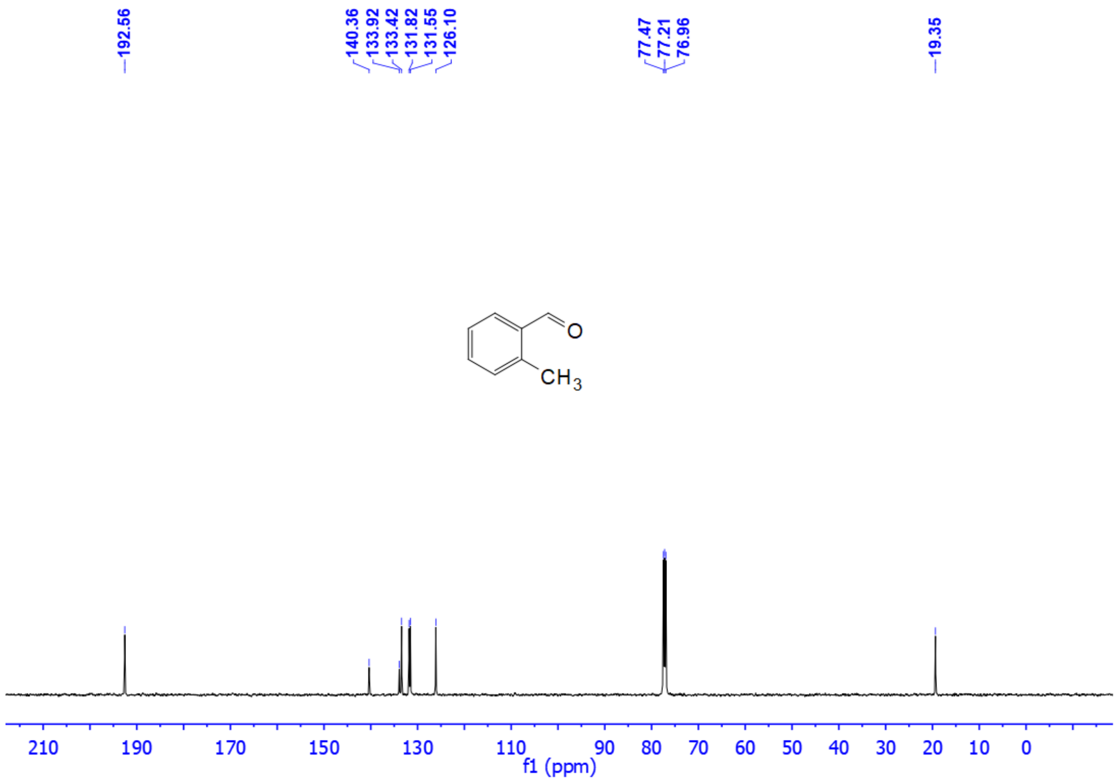

The ^{1}H and ^{13}C NMR spectra of compounds (2c)

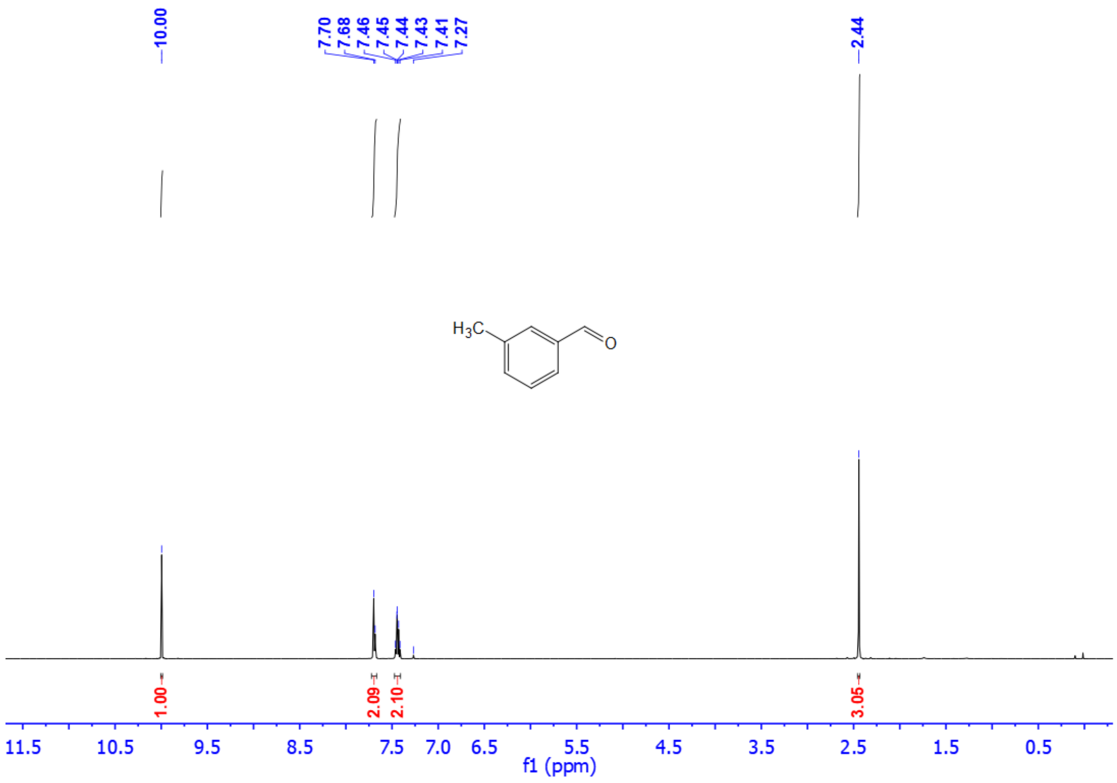

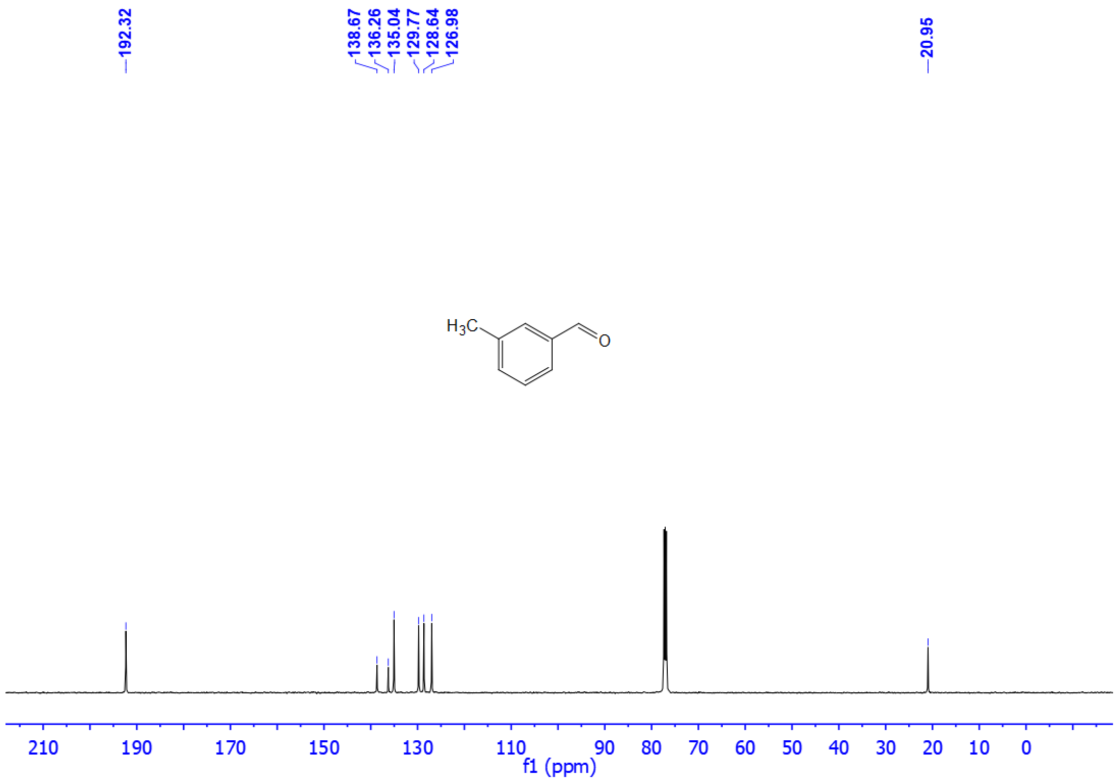

The ^{1}H and ^{13}C NMR spectra of compounds (2d)

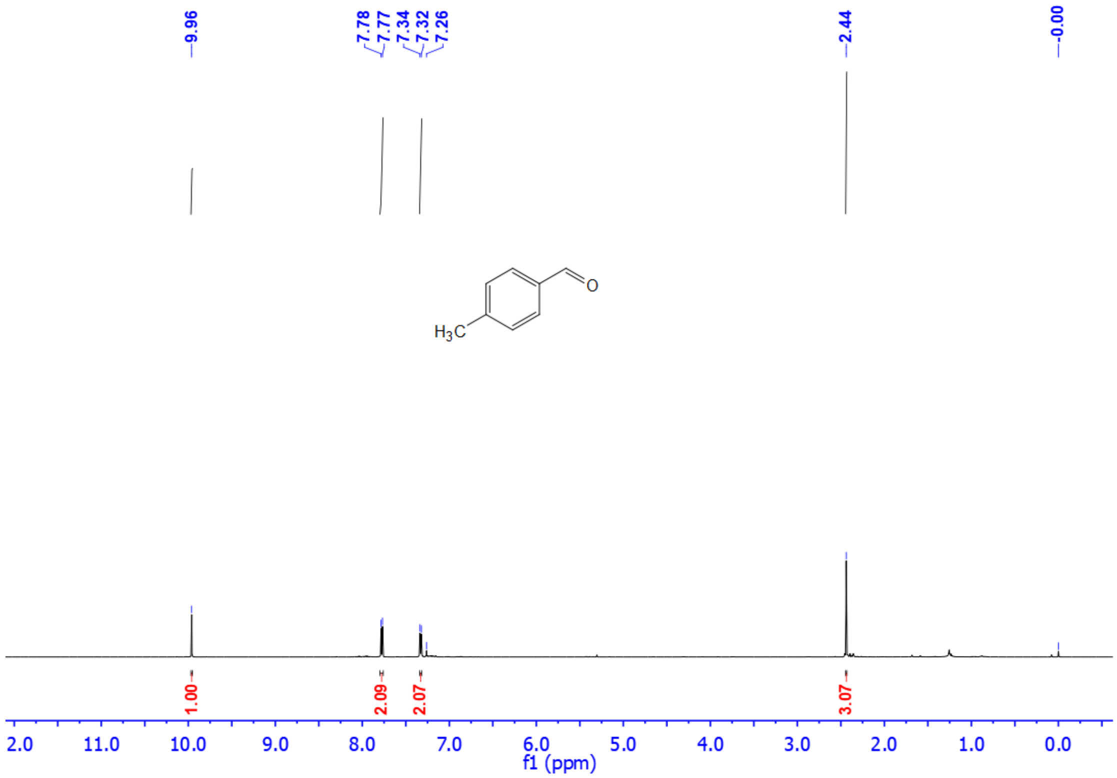

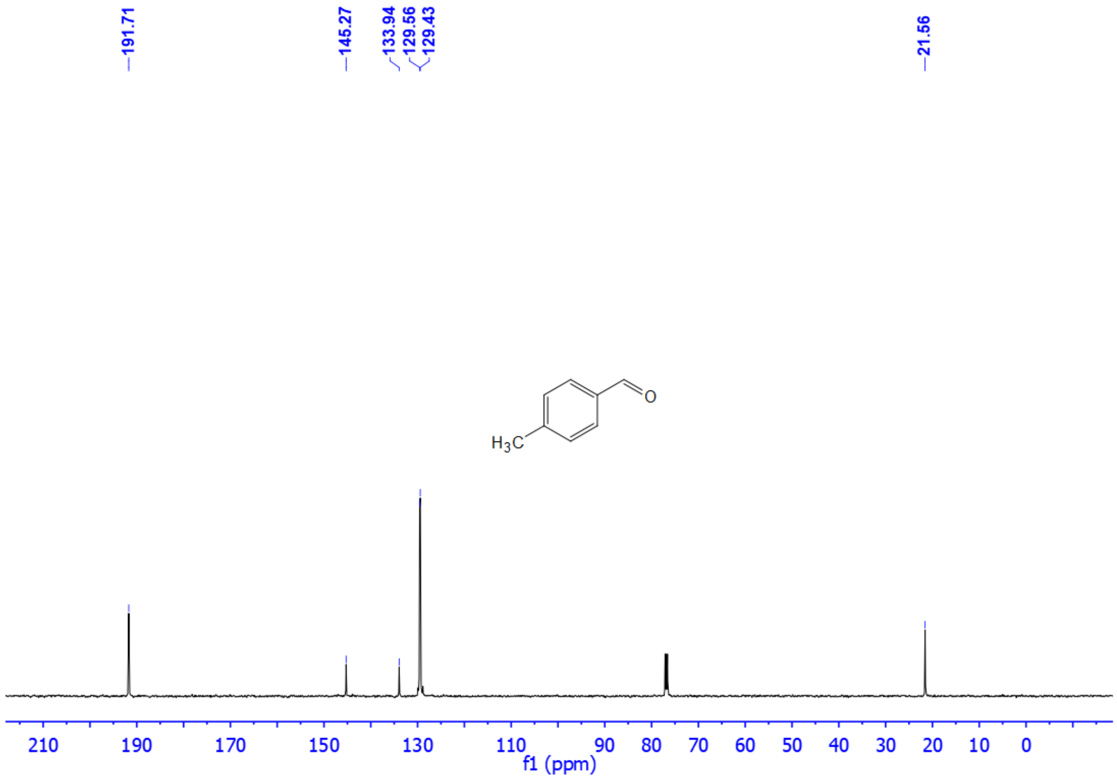

The ^{1}H and ^{13}C NMR spectra of compounds (2e)

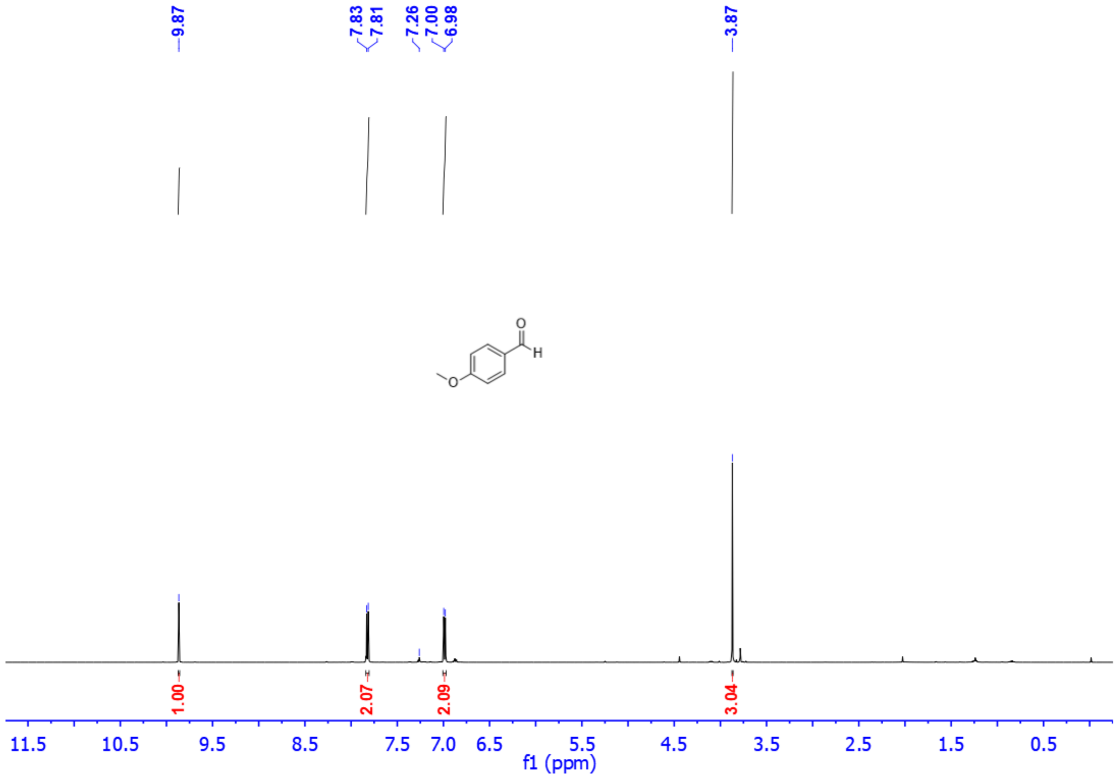

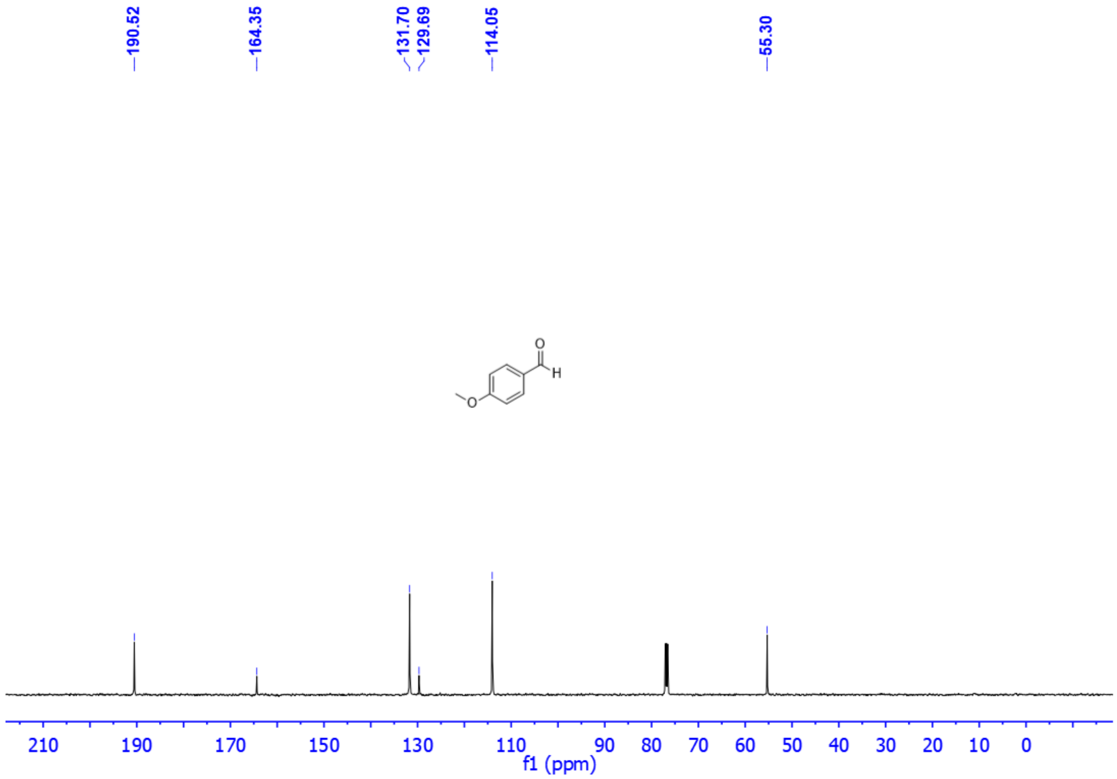

The ^{1}H and ^{13}C NMR spectra of compounds (2f)

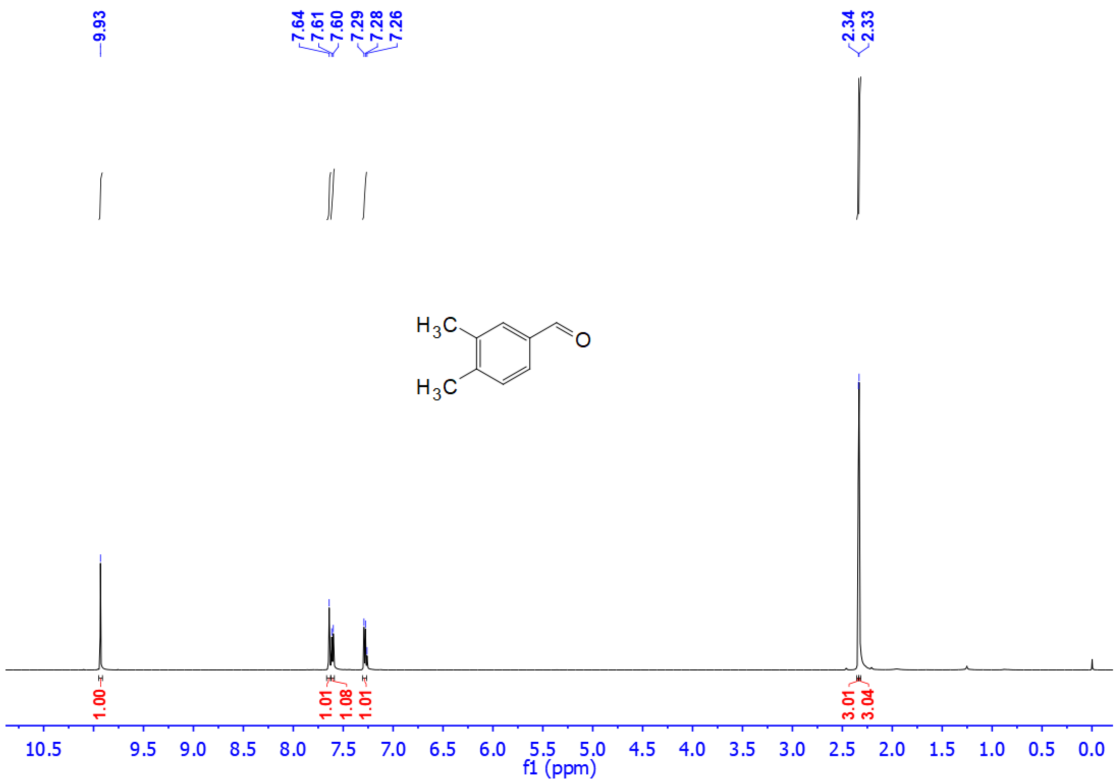

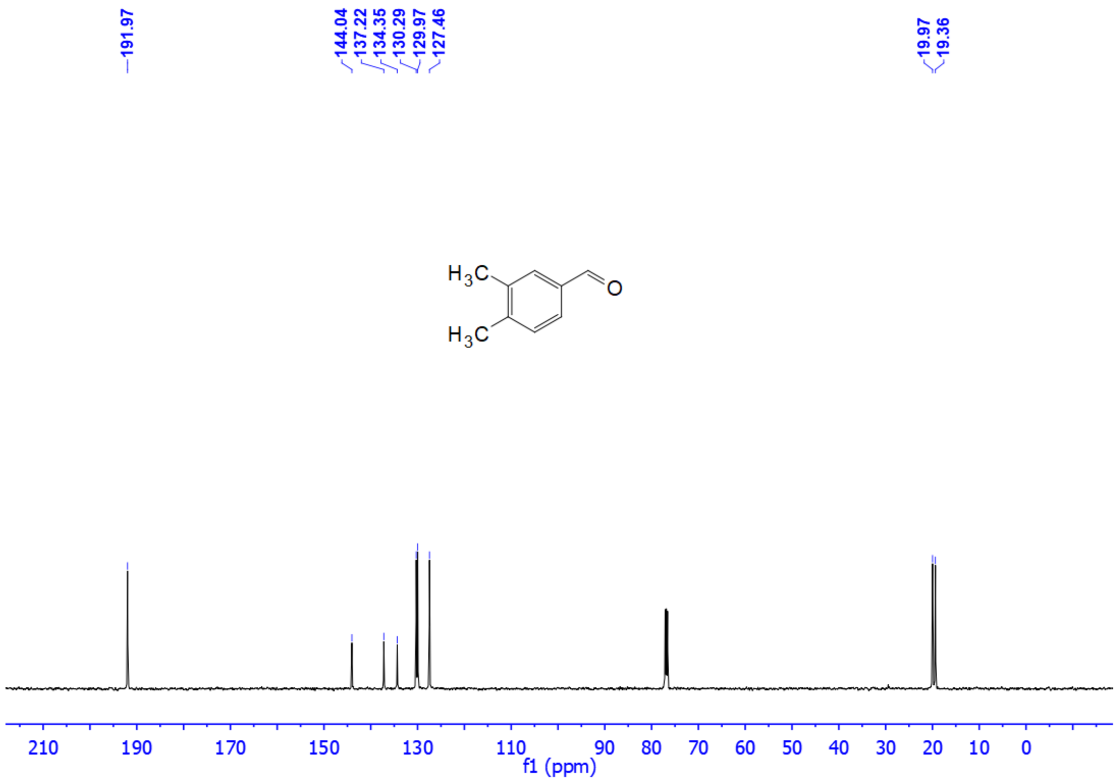

The ^{1}H and ^{13}C NMR spectra of compounds (2g)

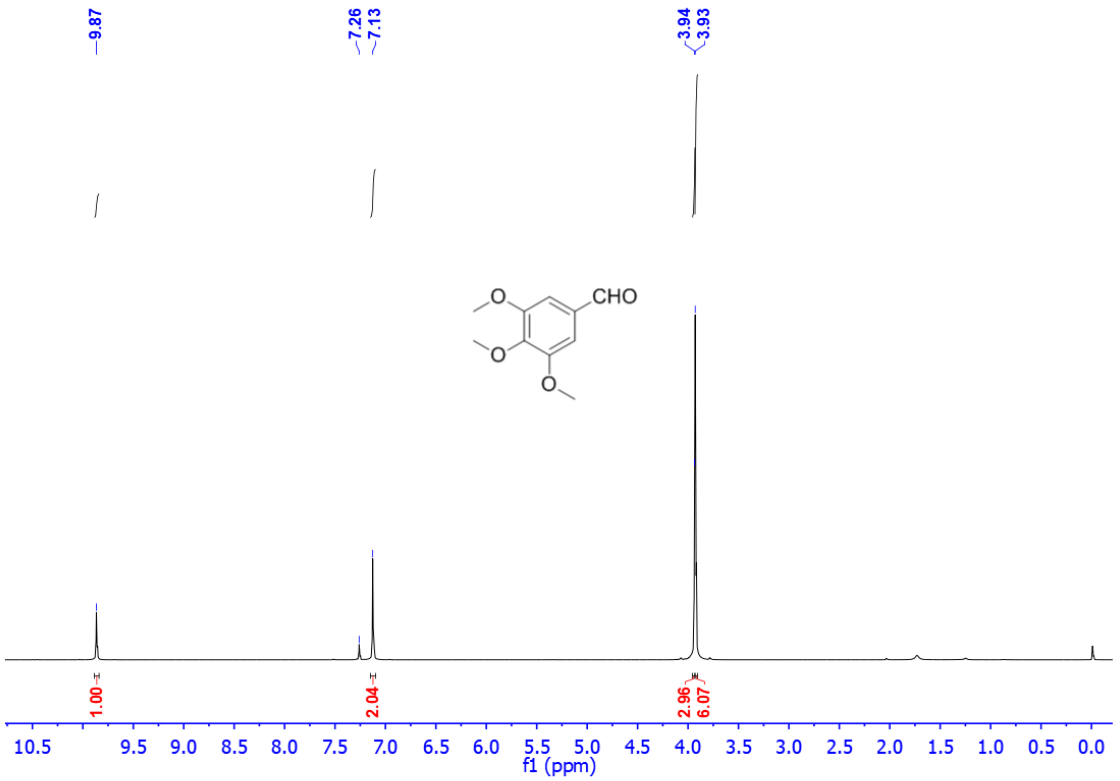

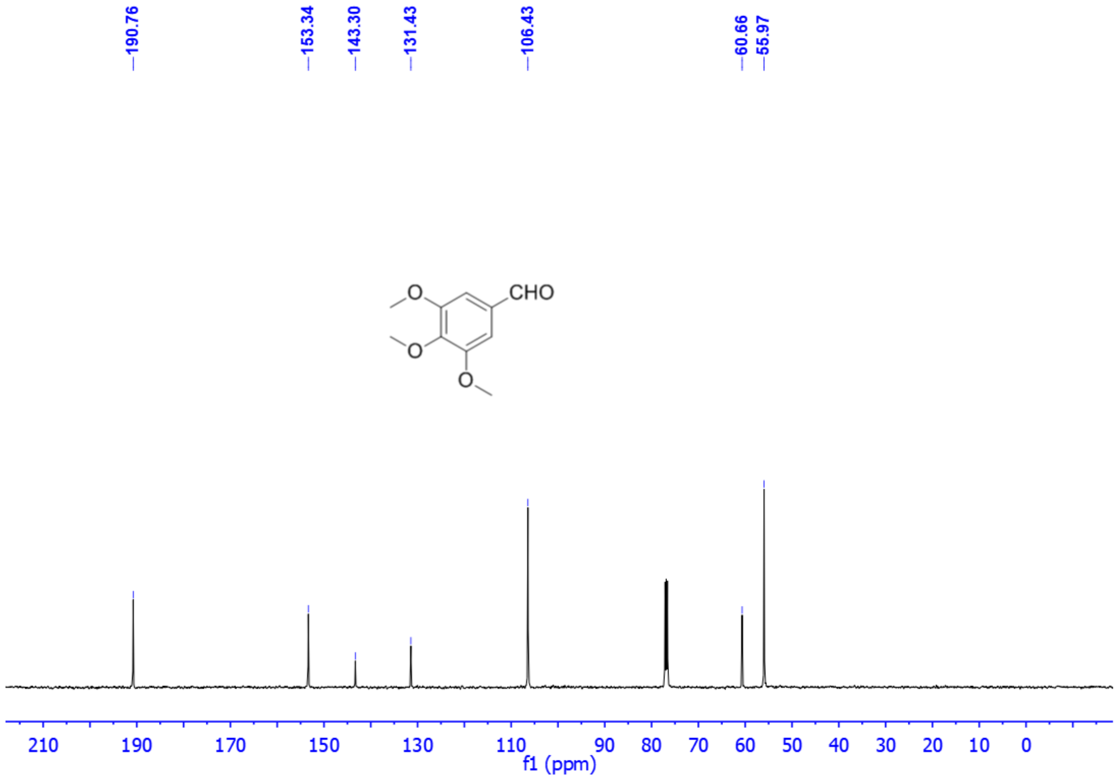

The ^{1}H and ^{13}C NMR spectra of compounds (2h)

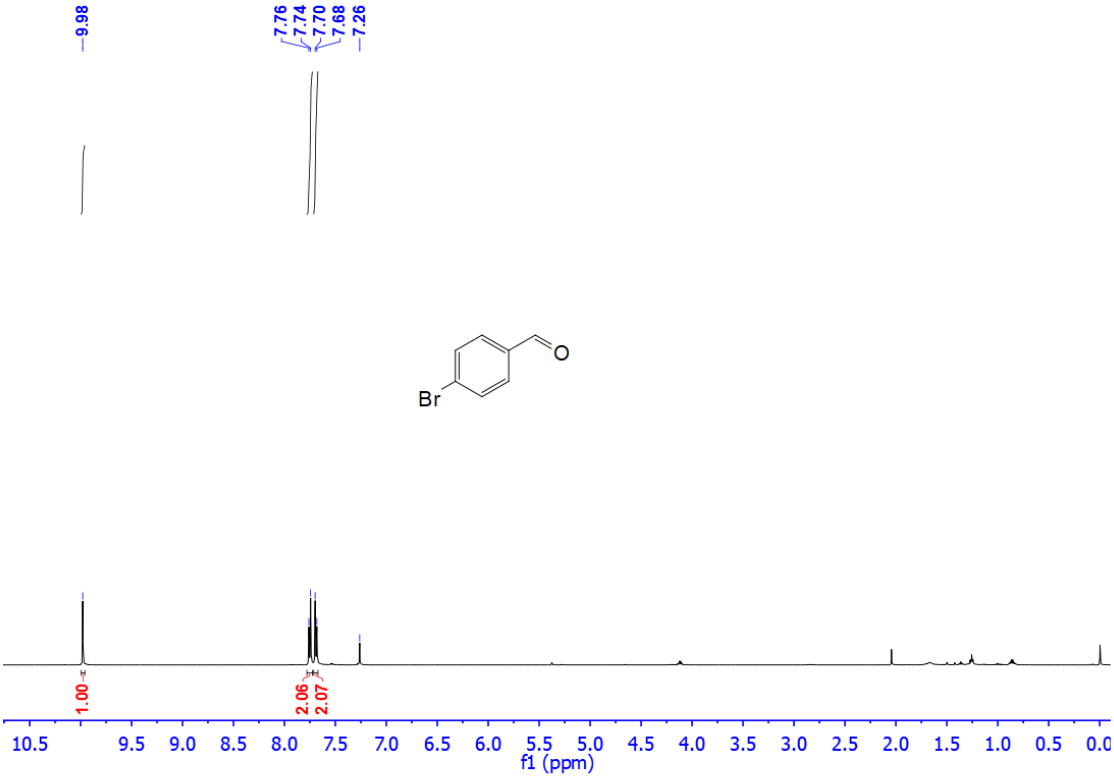

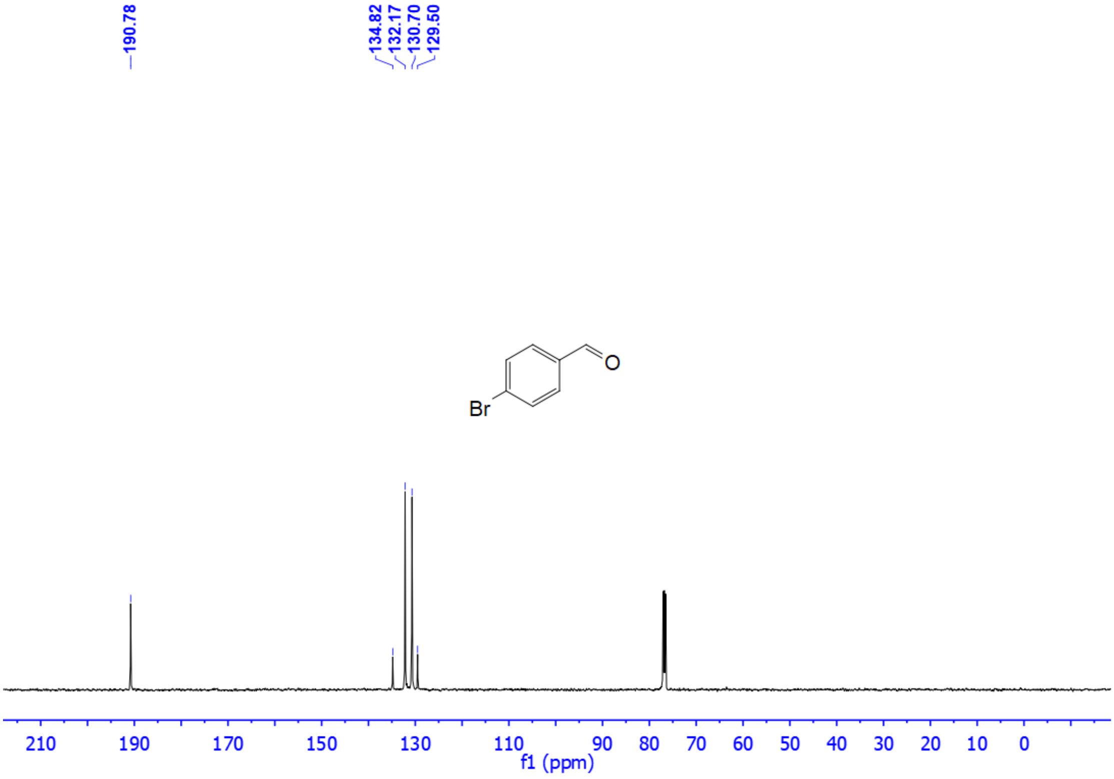

The ^{1}H and ^{13}C NMR spectra of compounds (2i)

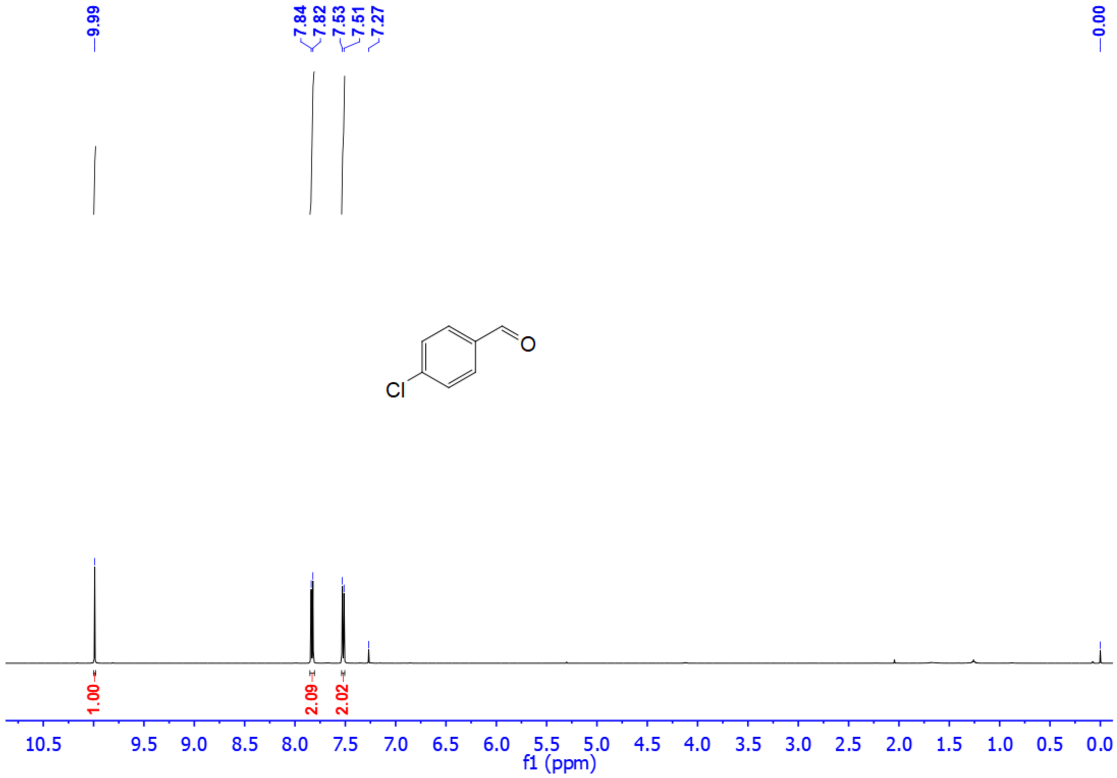

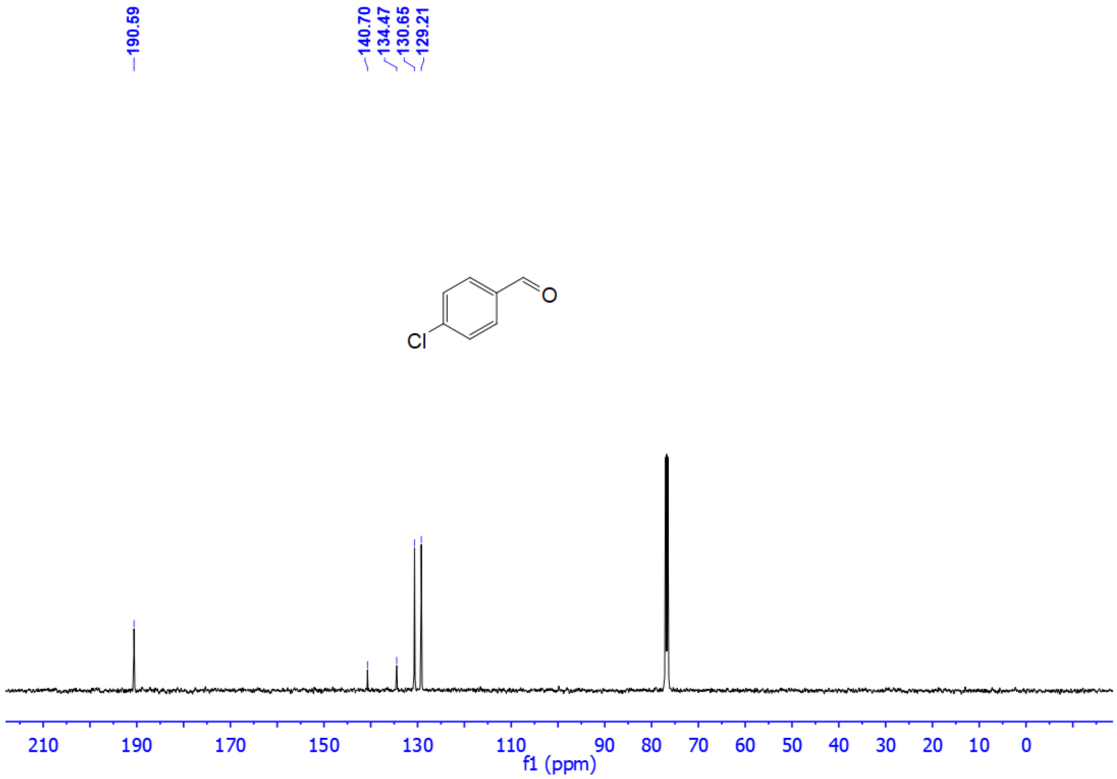

The ^{1}H and ^{13}C NMR spectra of compounds (2j)

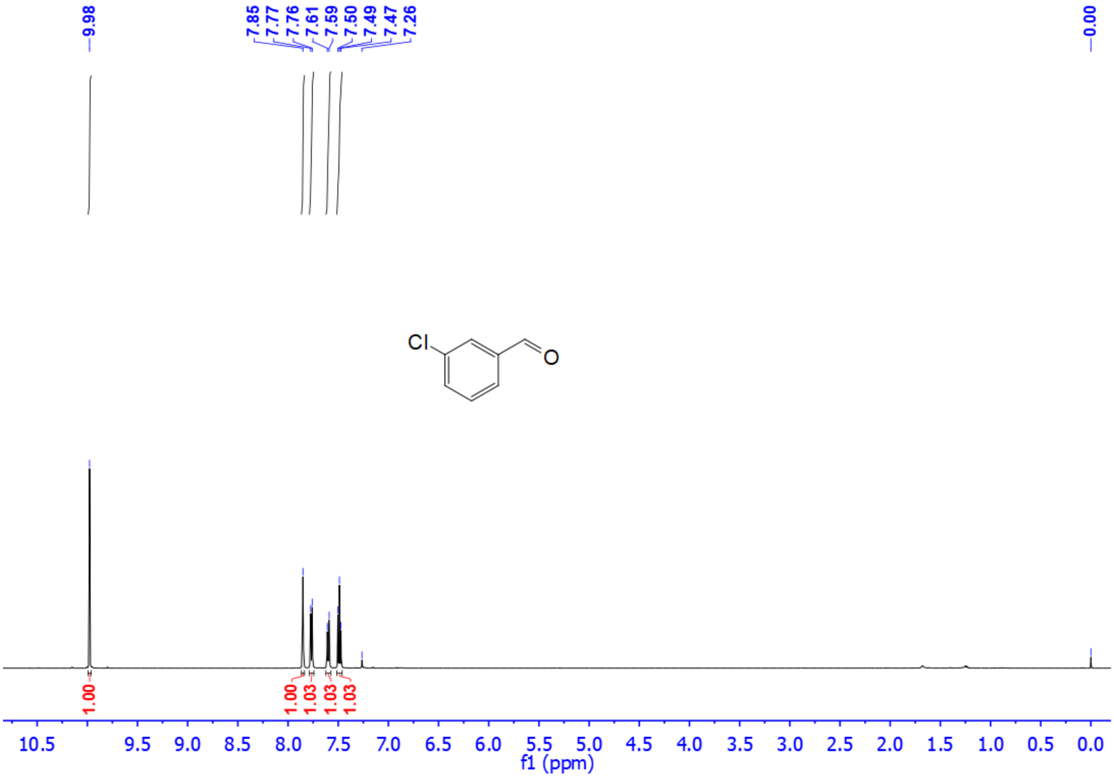

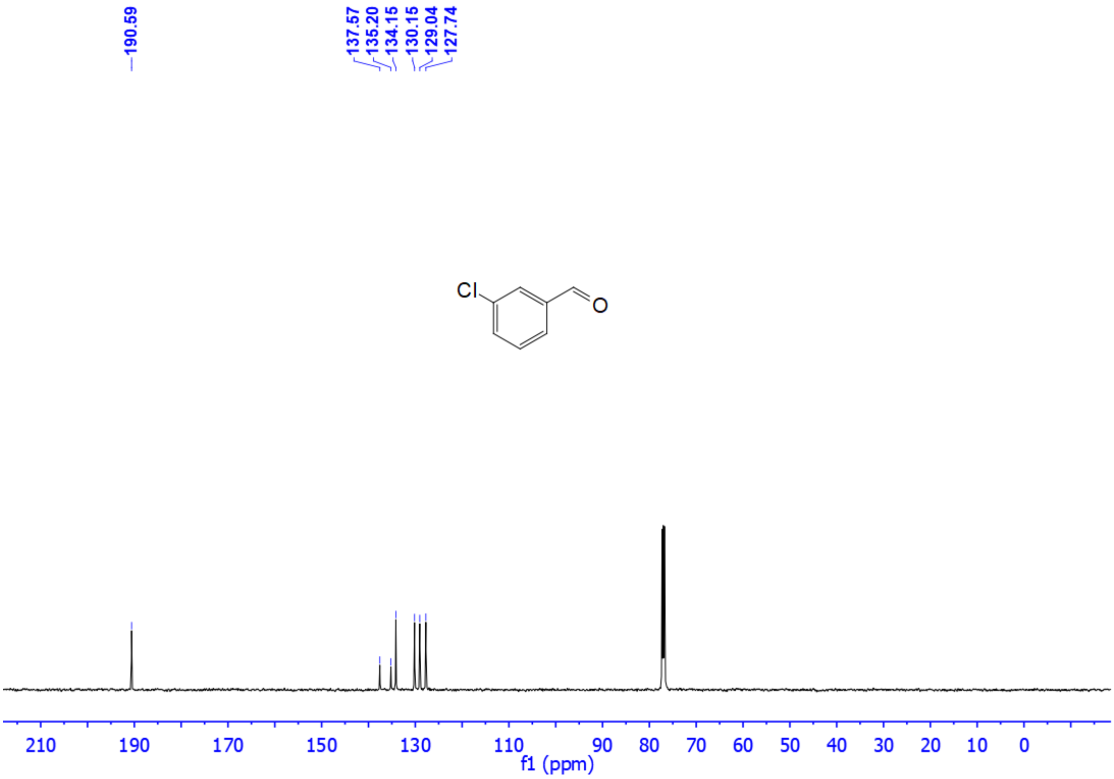

The ^{1}H and ^{13}C NMR spectra of compounds (2k)

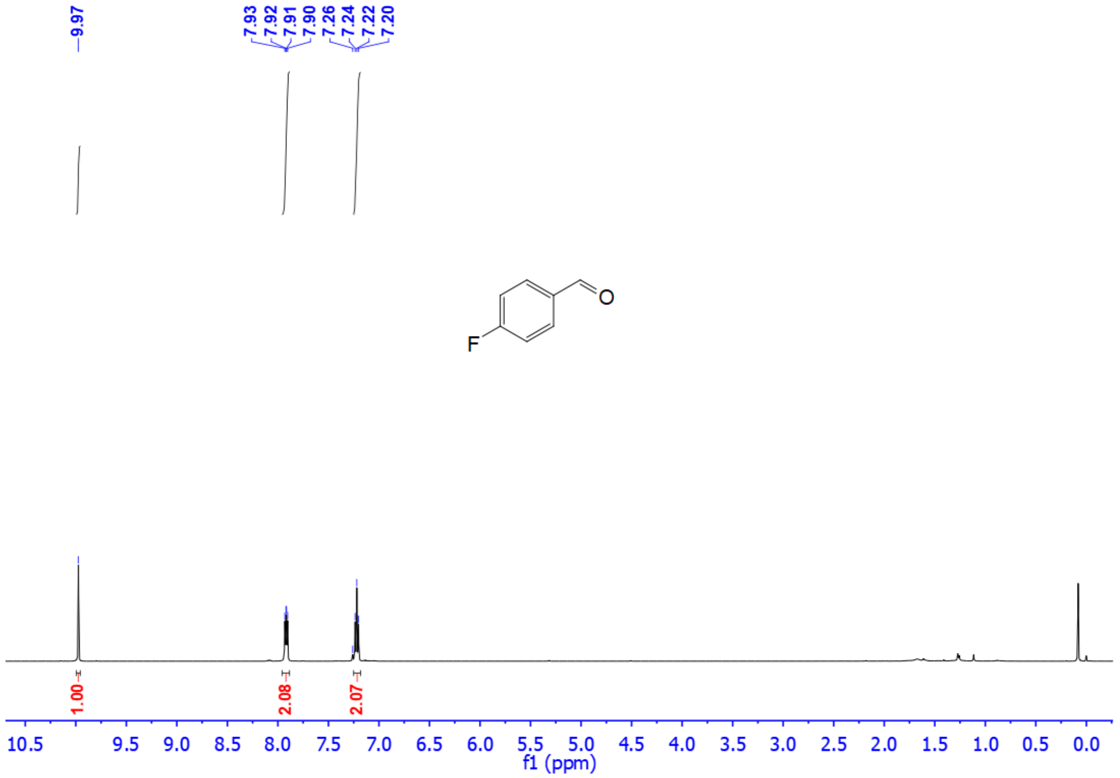

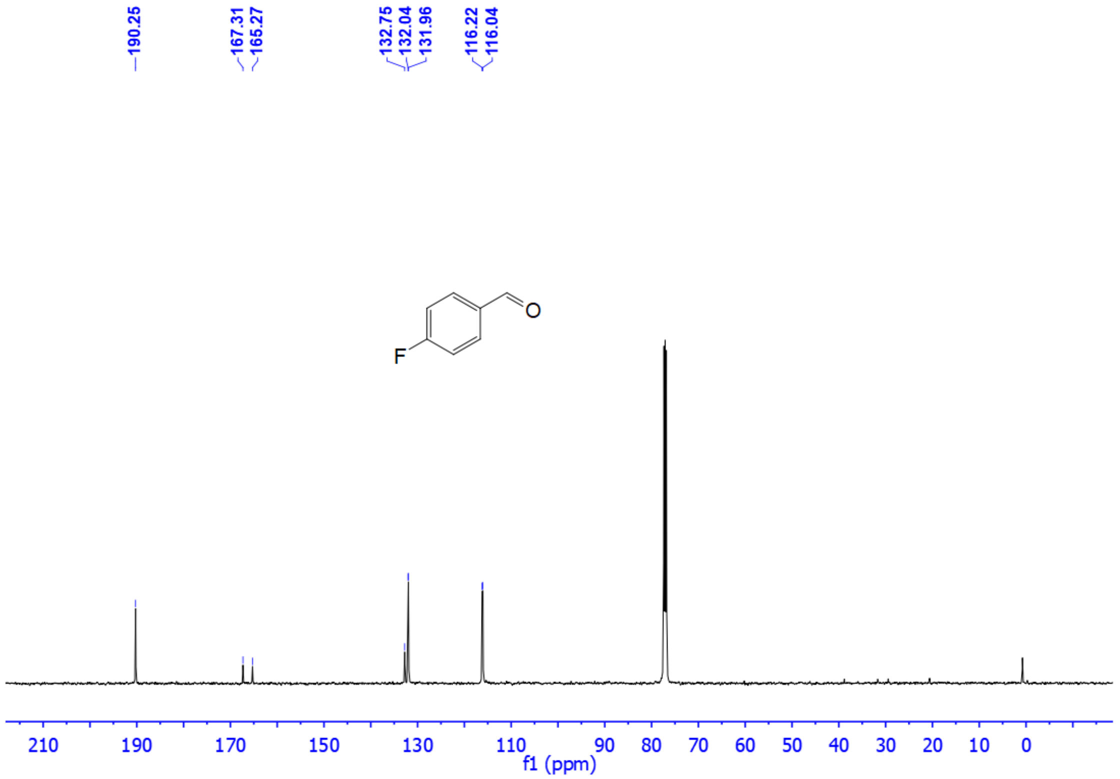

The ^{1}H and ^{13}C NMR spectra of compounds (2l)

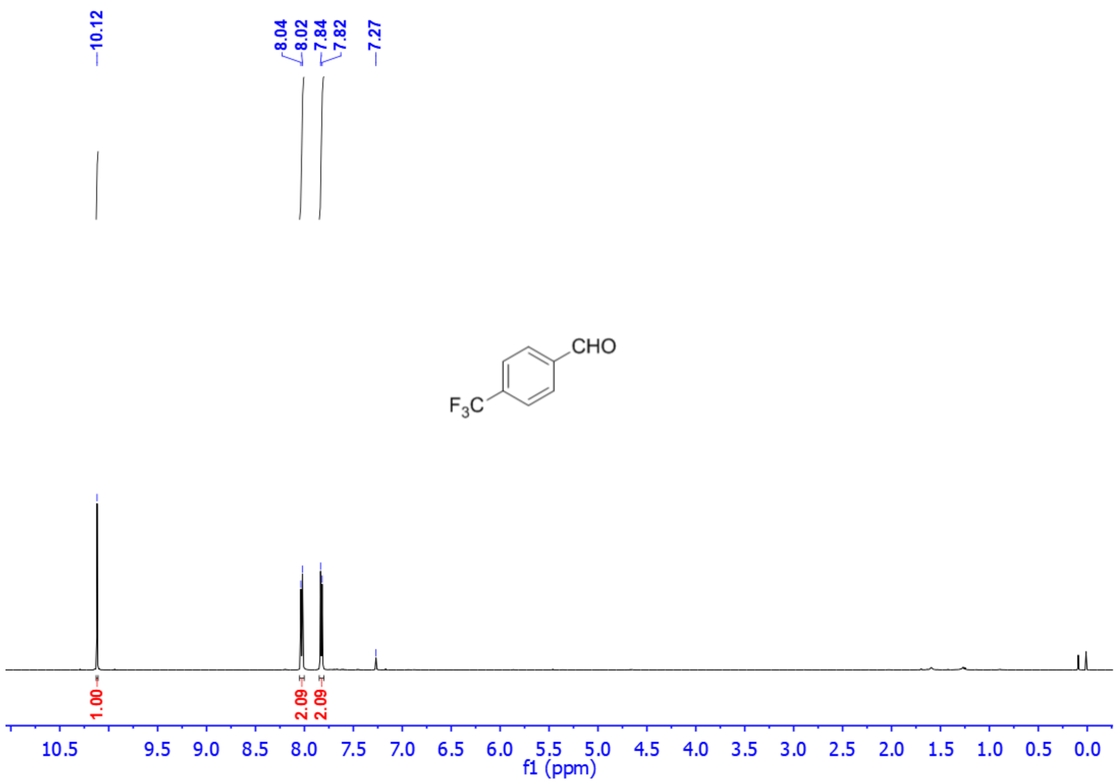

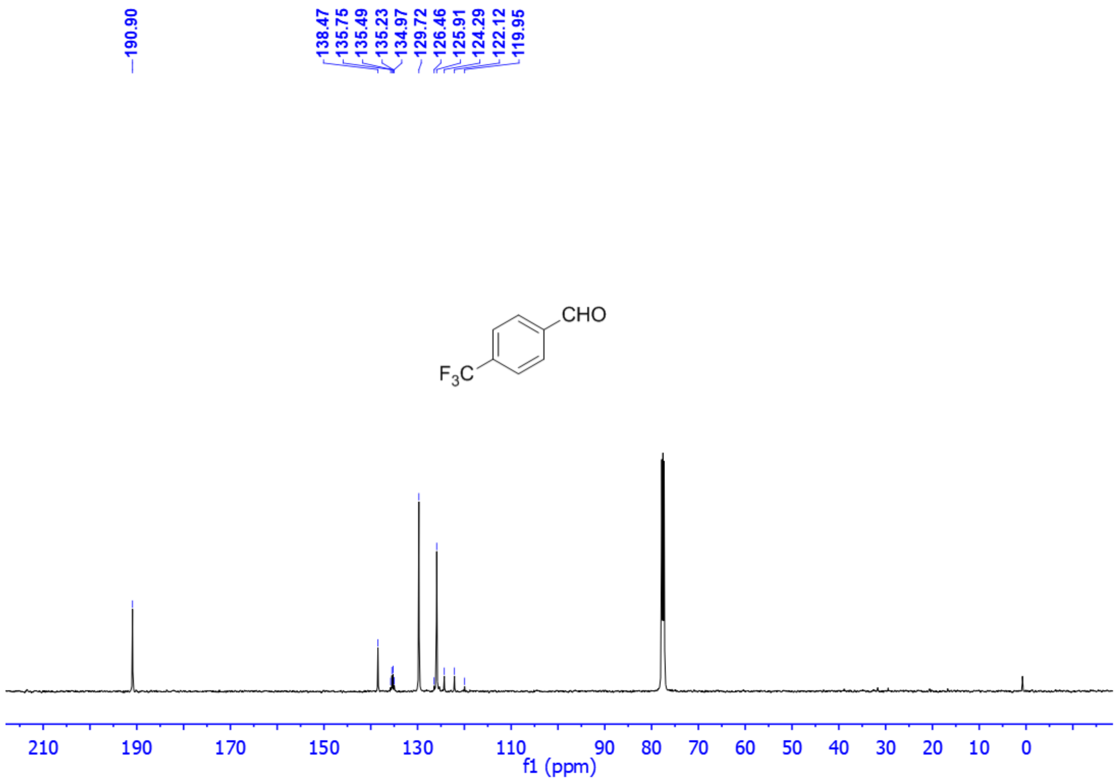

The ^{1}H and ^{13}C NMR spectra of compounds (2m)

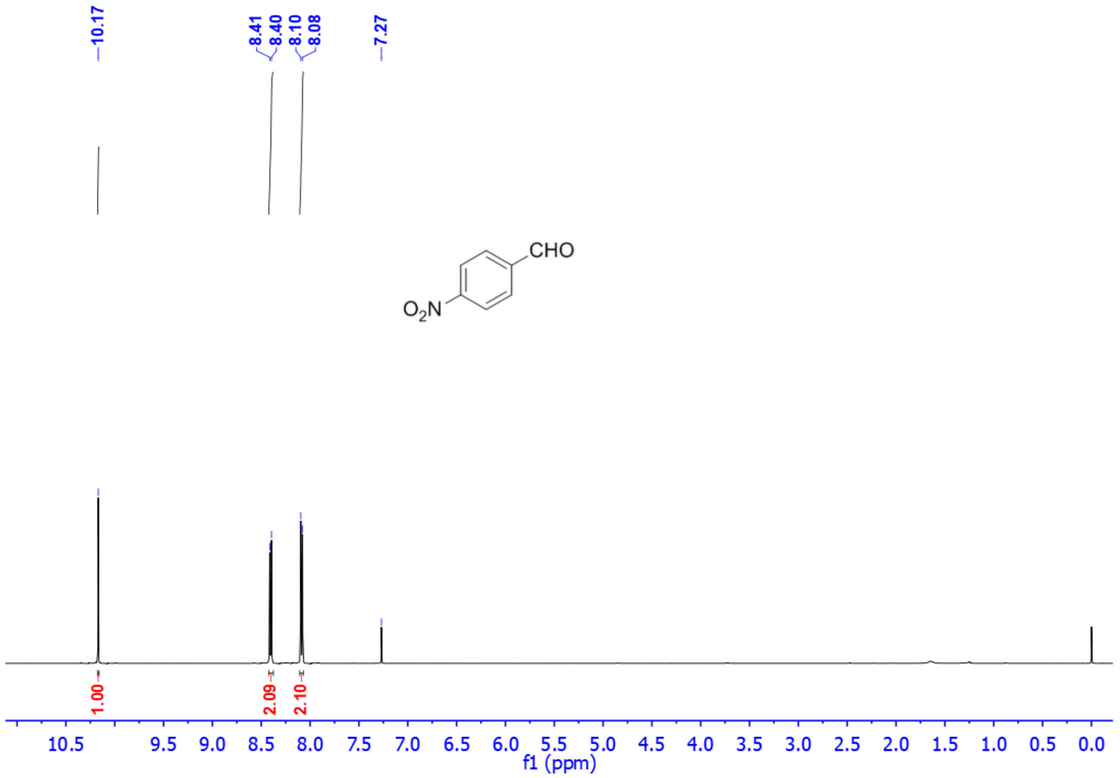

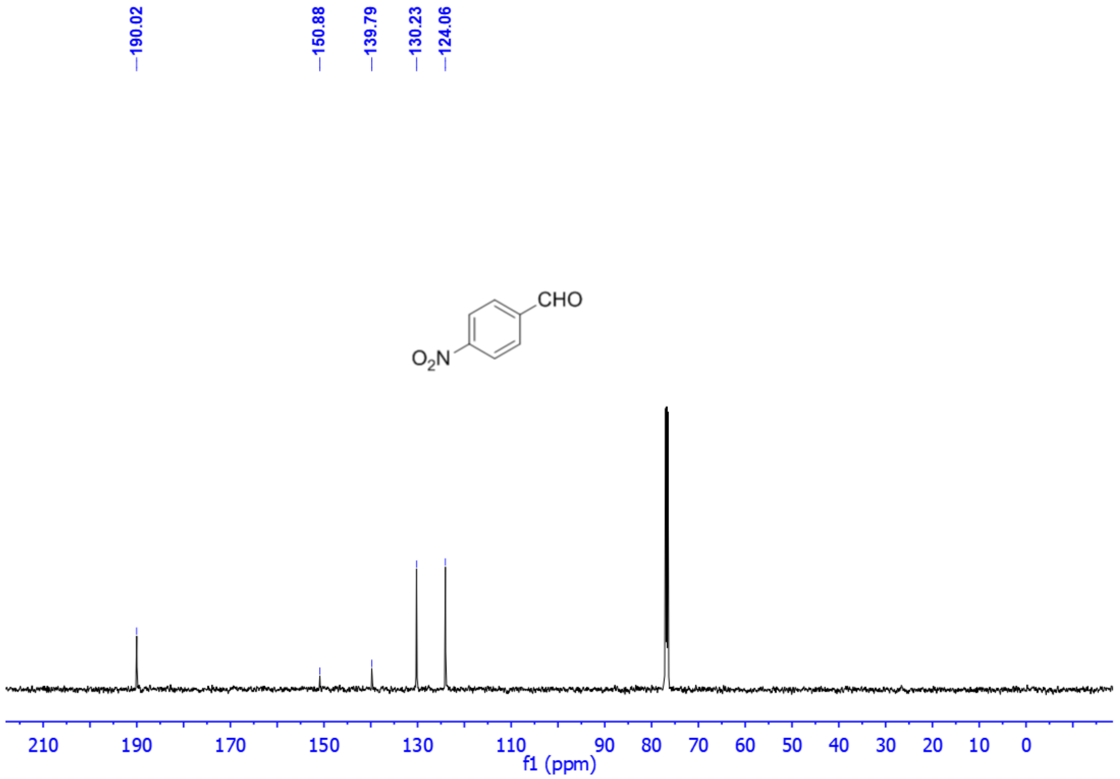

The ^{1}H and ^{13}C NMR spectra of compounds (2n)

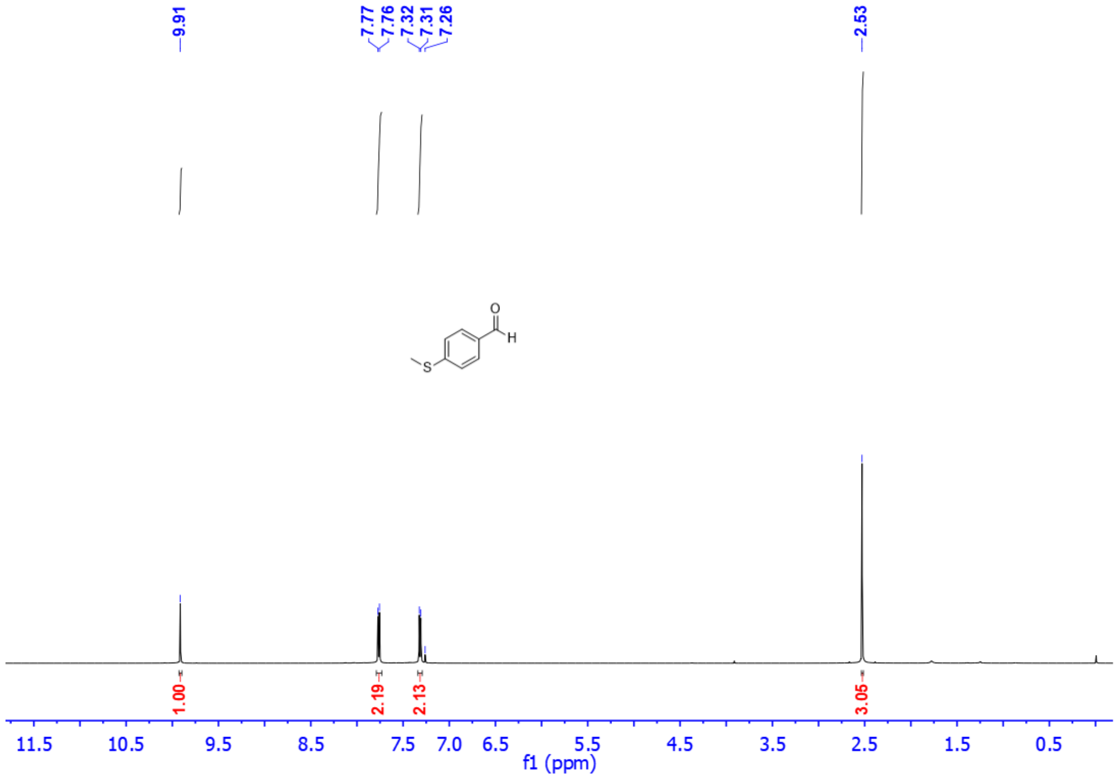

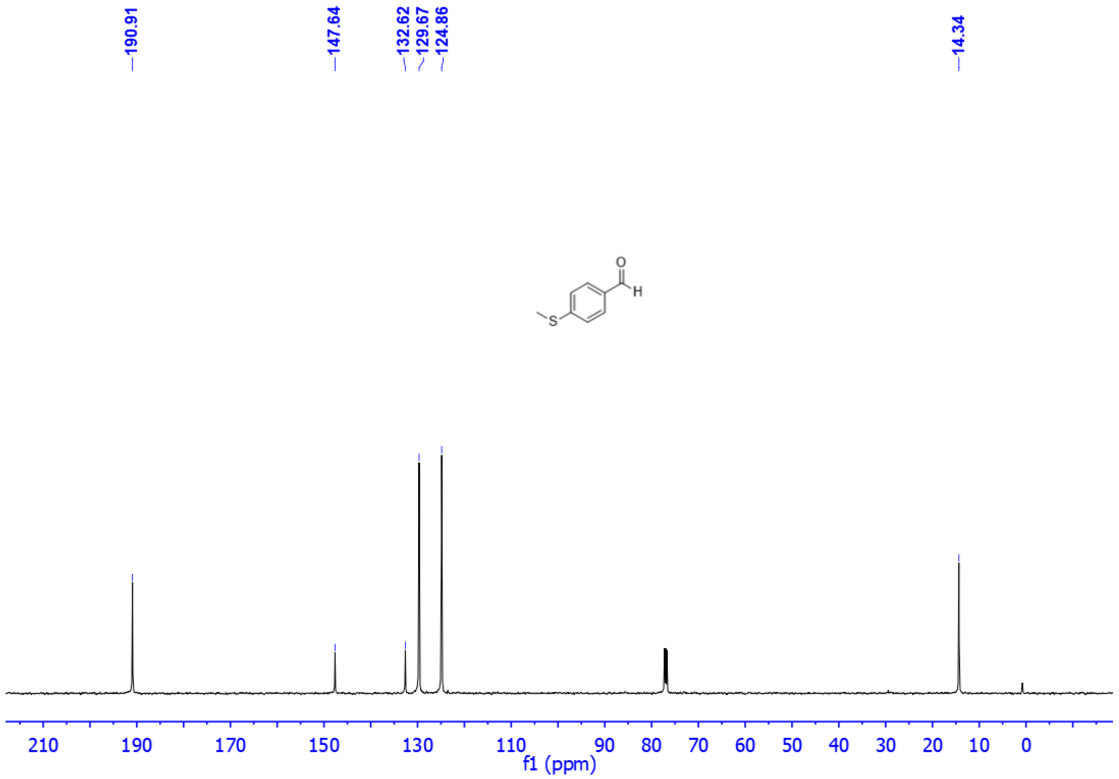

The ^{1}H and ^{13}C NMR spectra of compounds (2o)

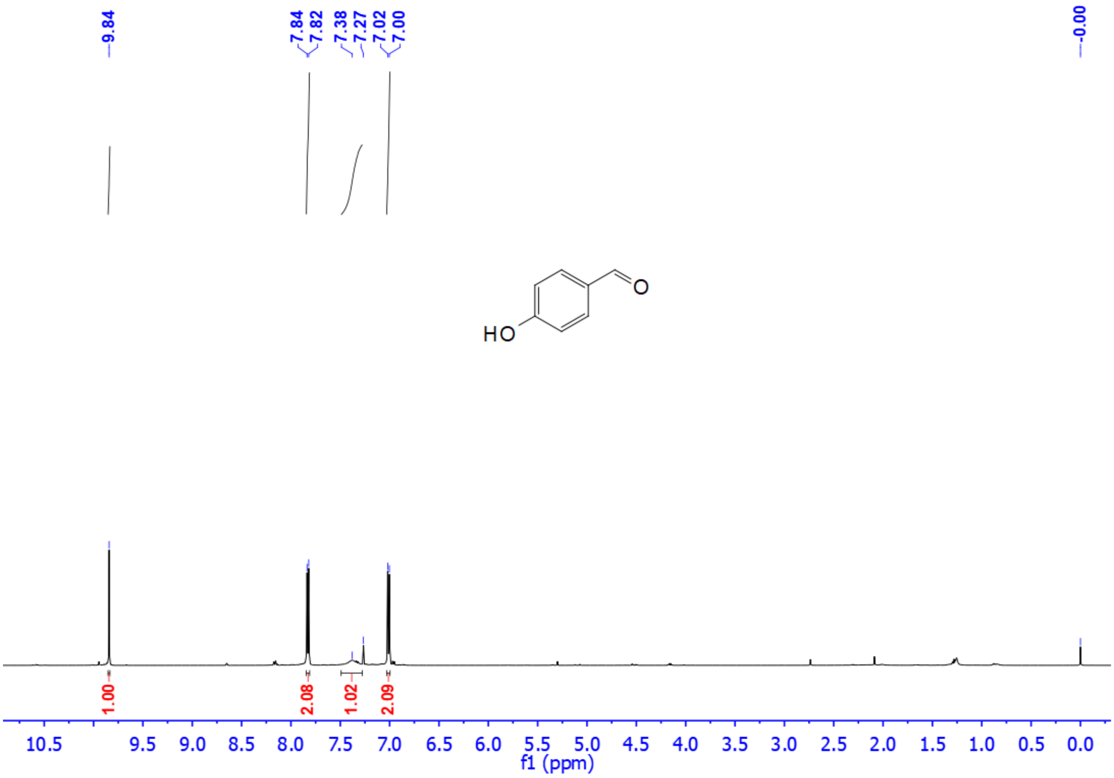

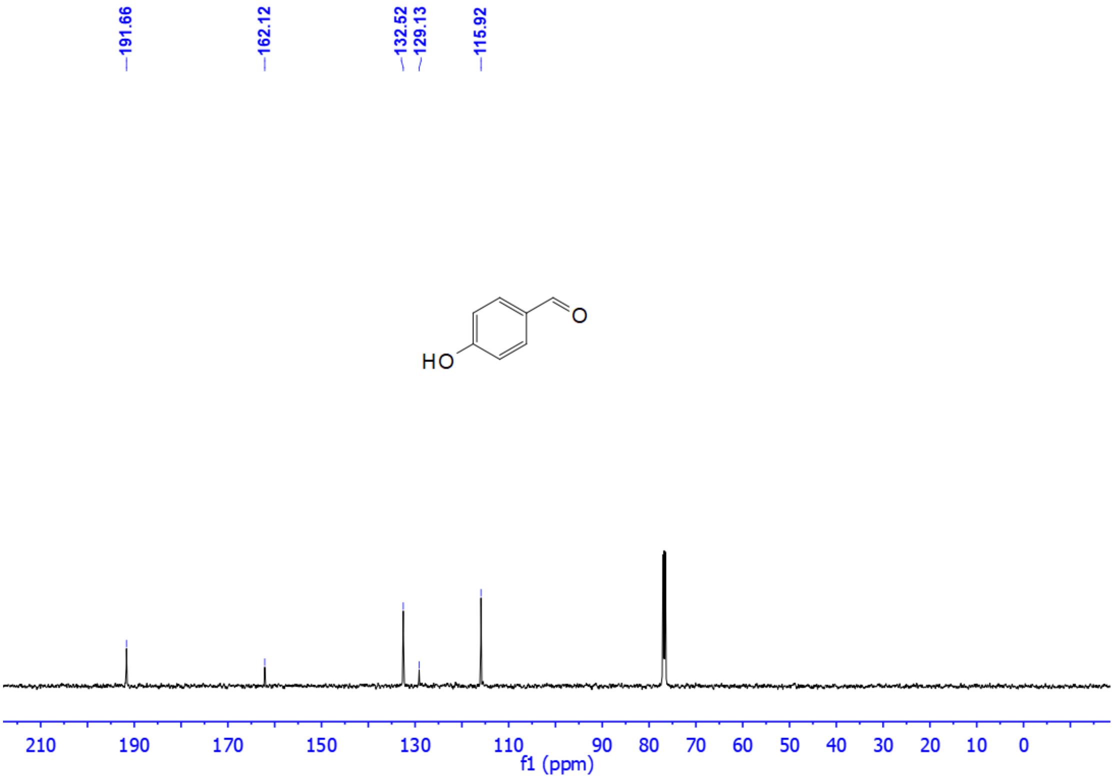

The ^{1}H and ^{13}C NMR spectra of compounds (2p)

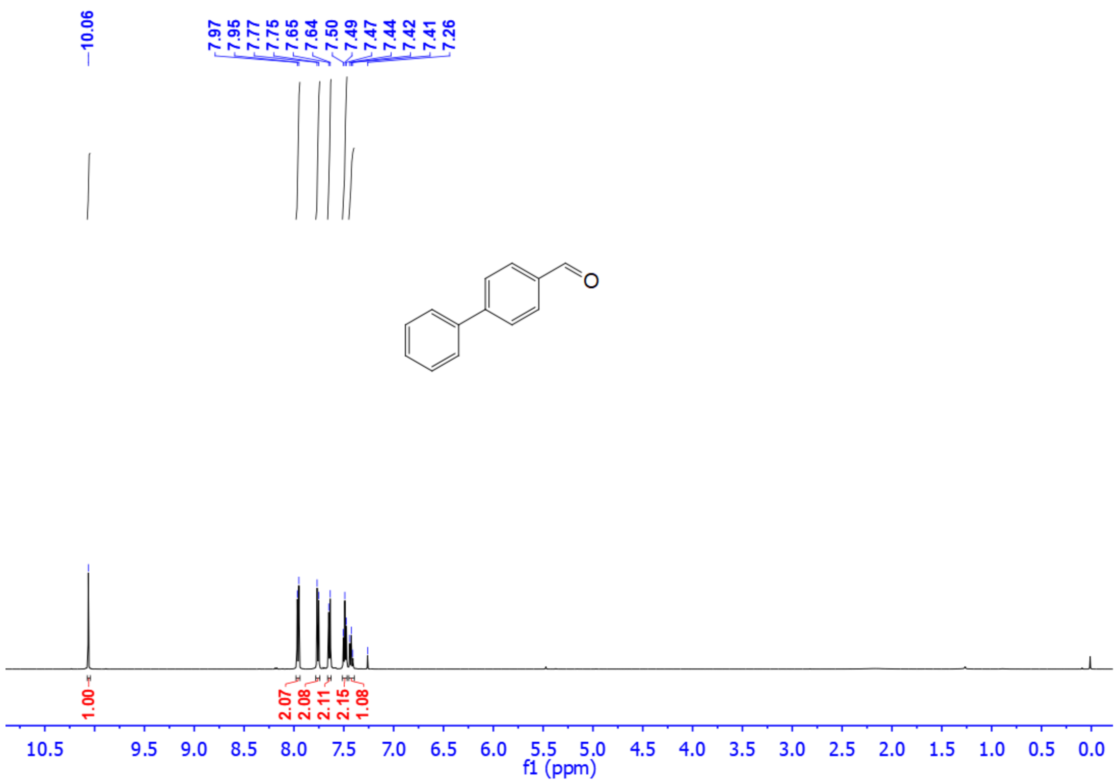

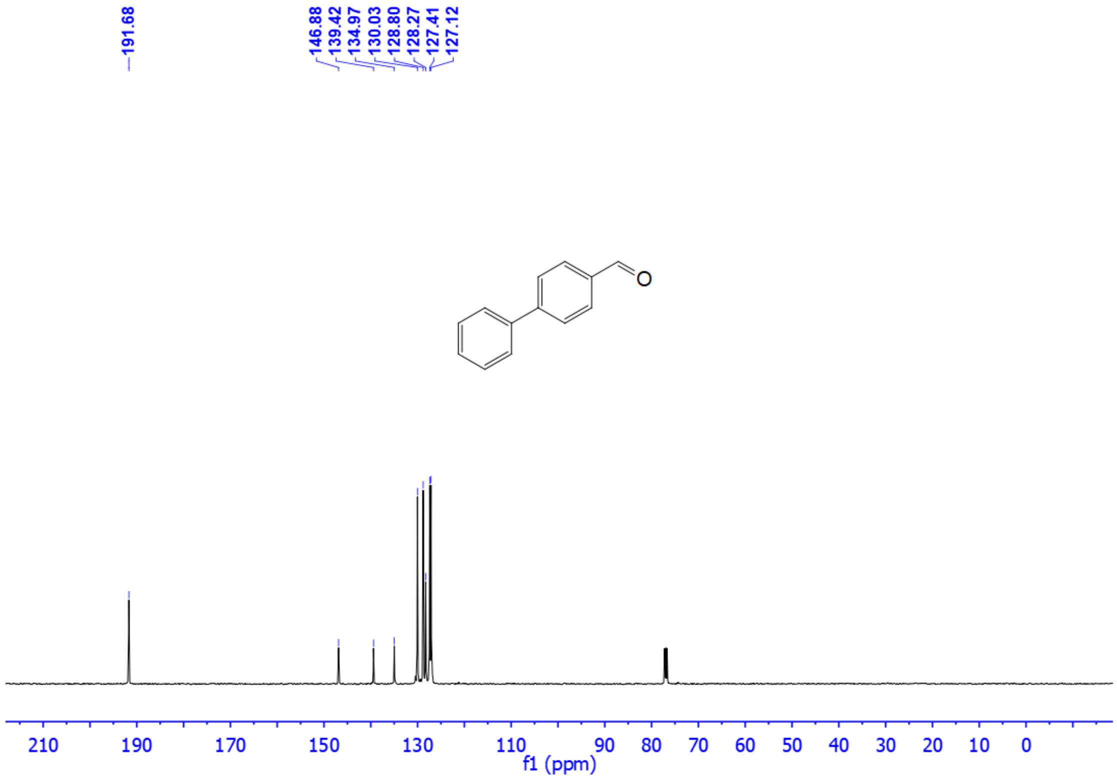

The ^{1}H and ^{13}C NMR spectra of compounds (2q)

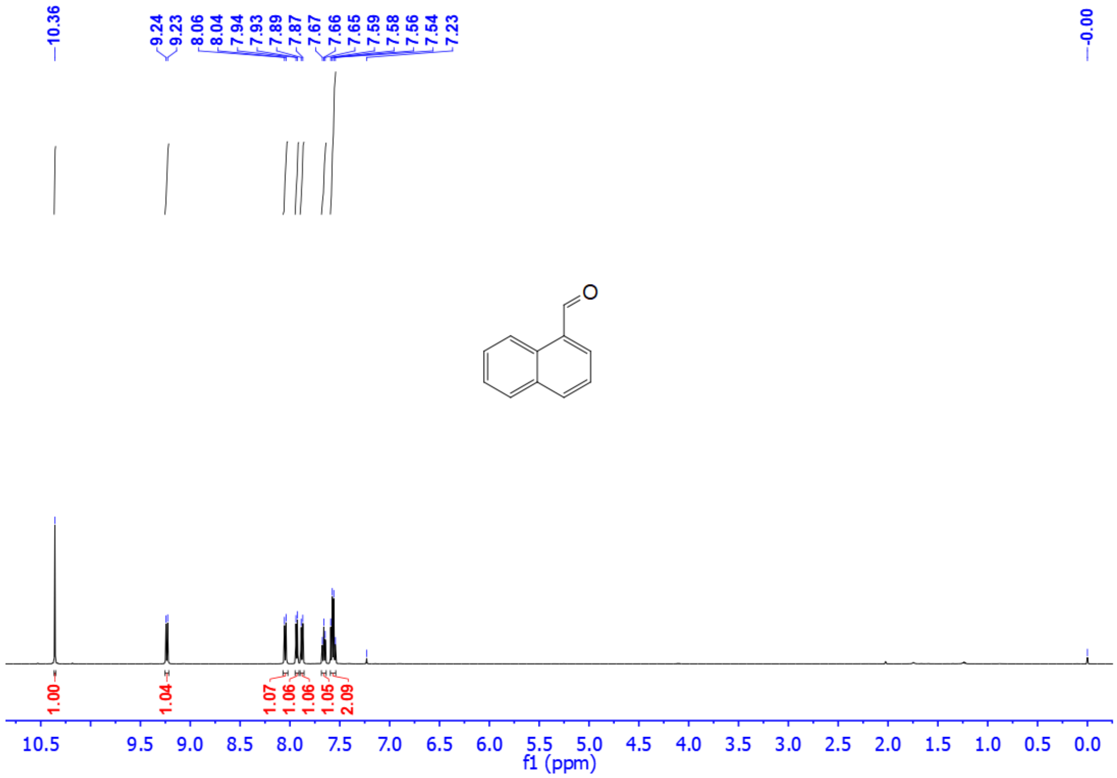

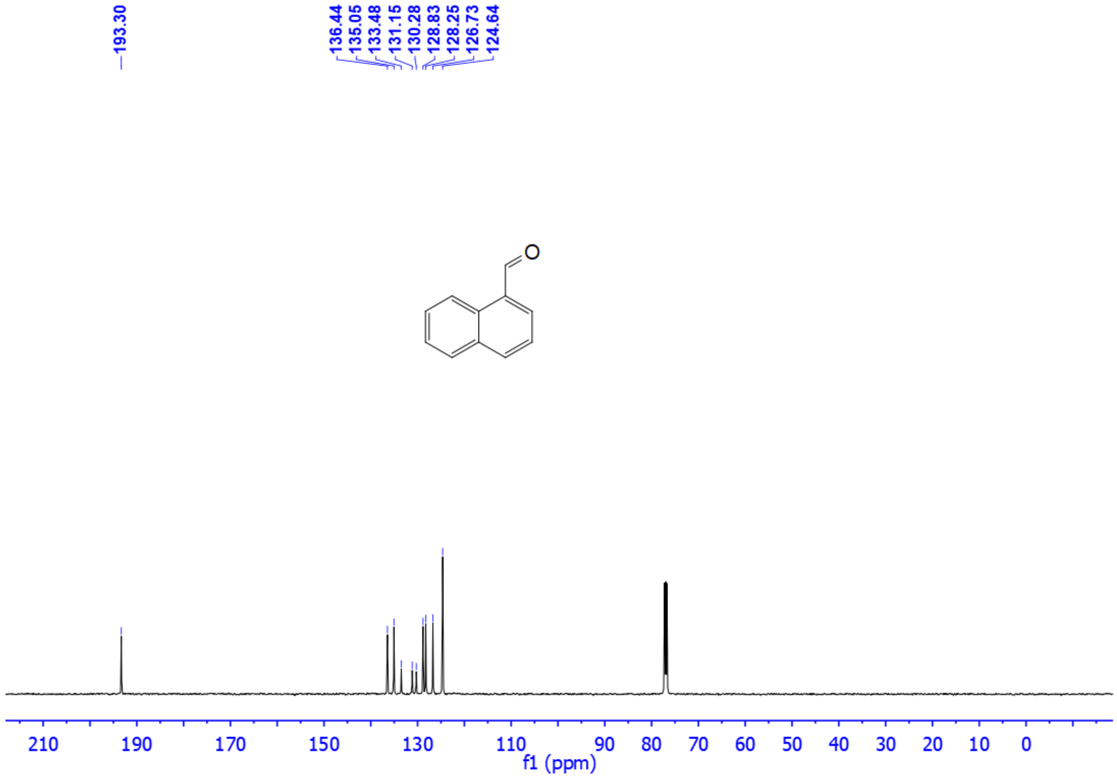

The ^{1}H and ^{13}C NMR spectra of compounds (2r)

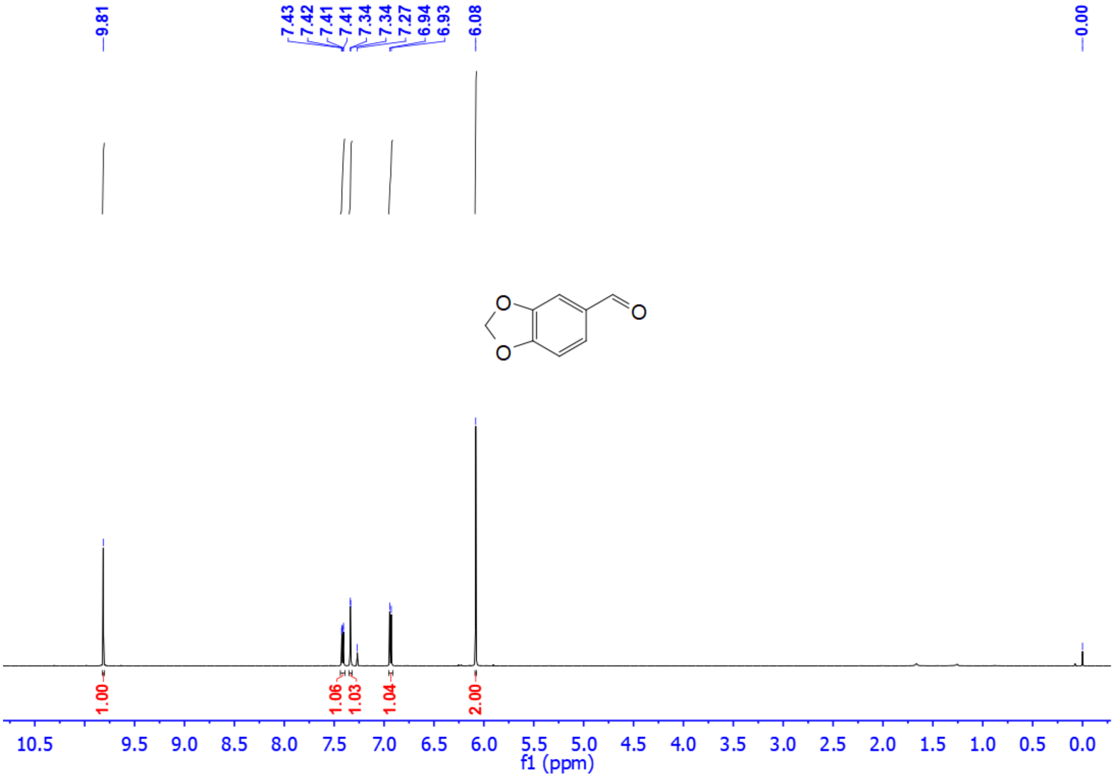

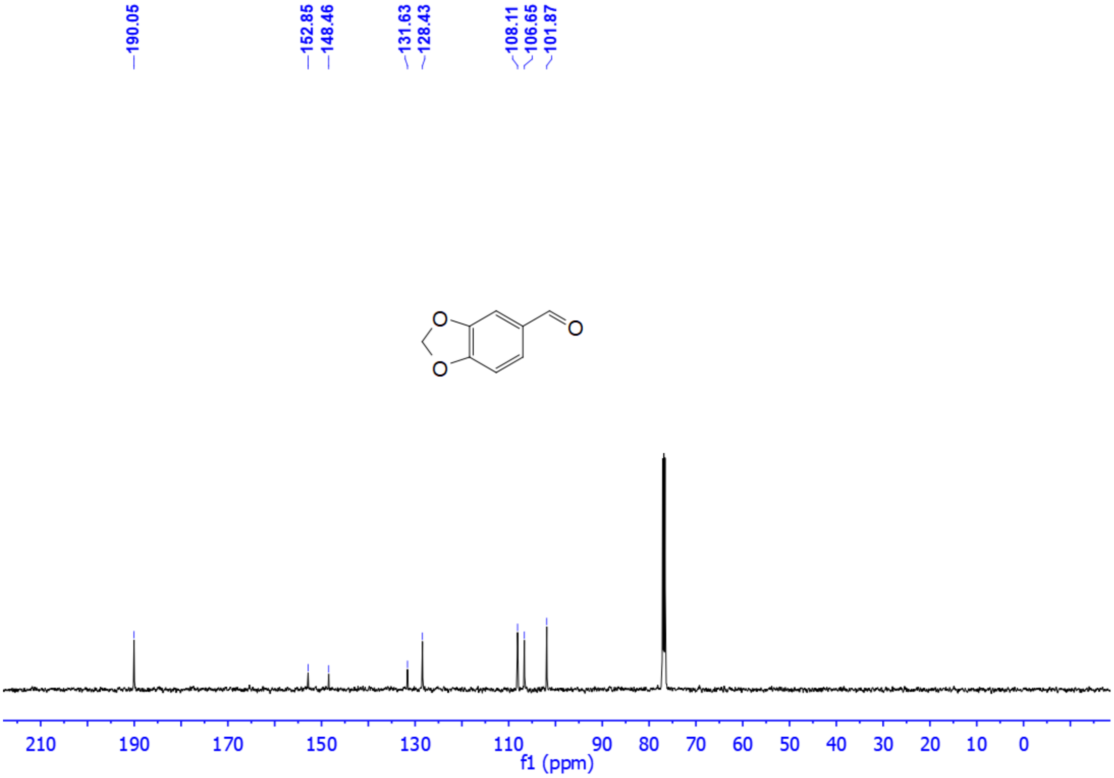

The ^{1}H and ^{13}C NMR spectra of compounds (2s)

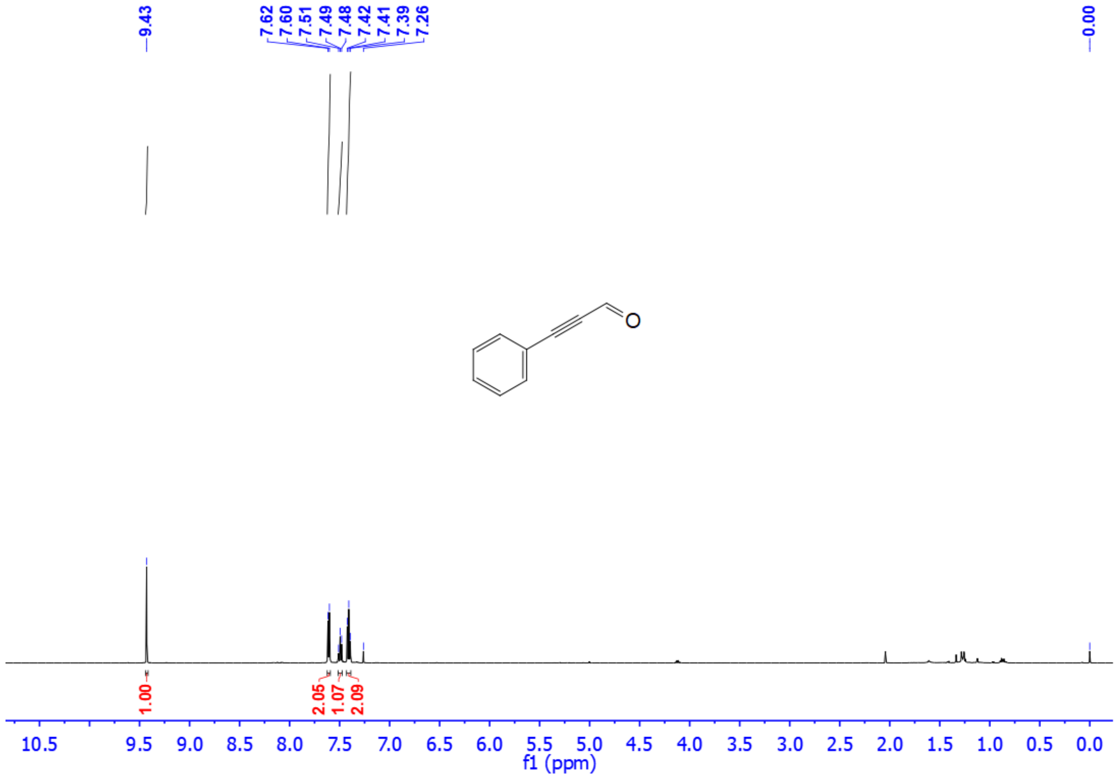

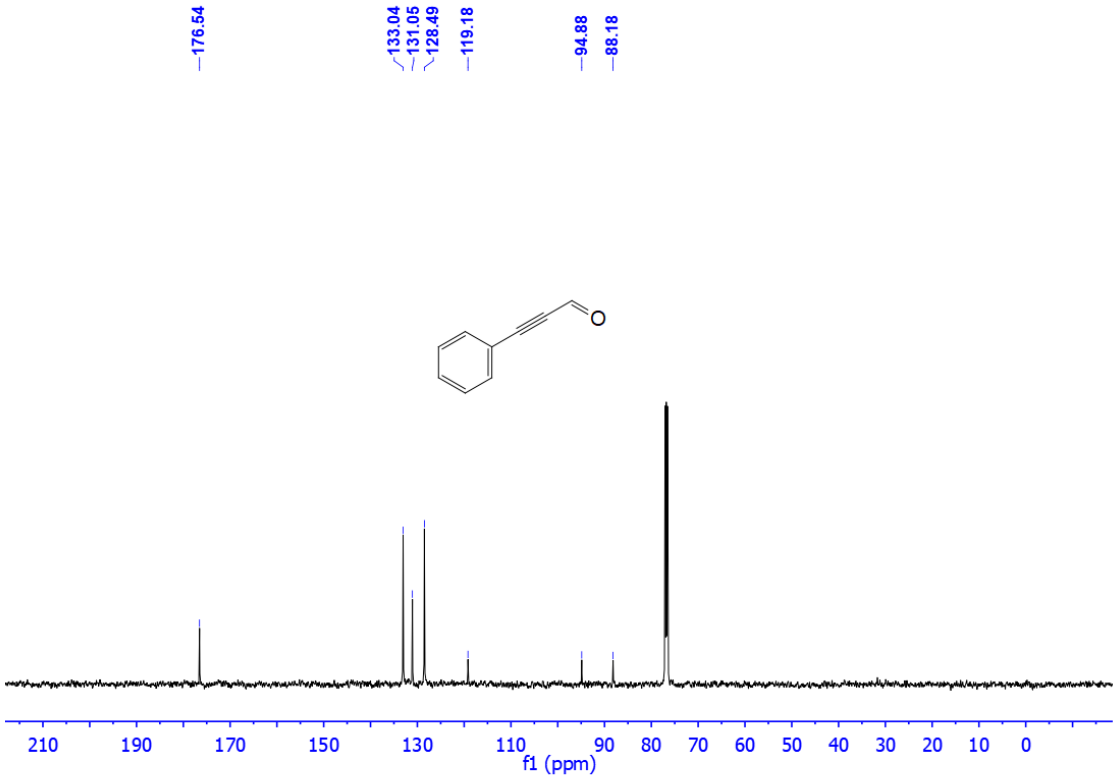

The ^{1}H and ^{13}C NMR spectra of compounds (2t)

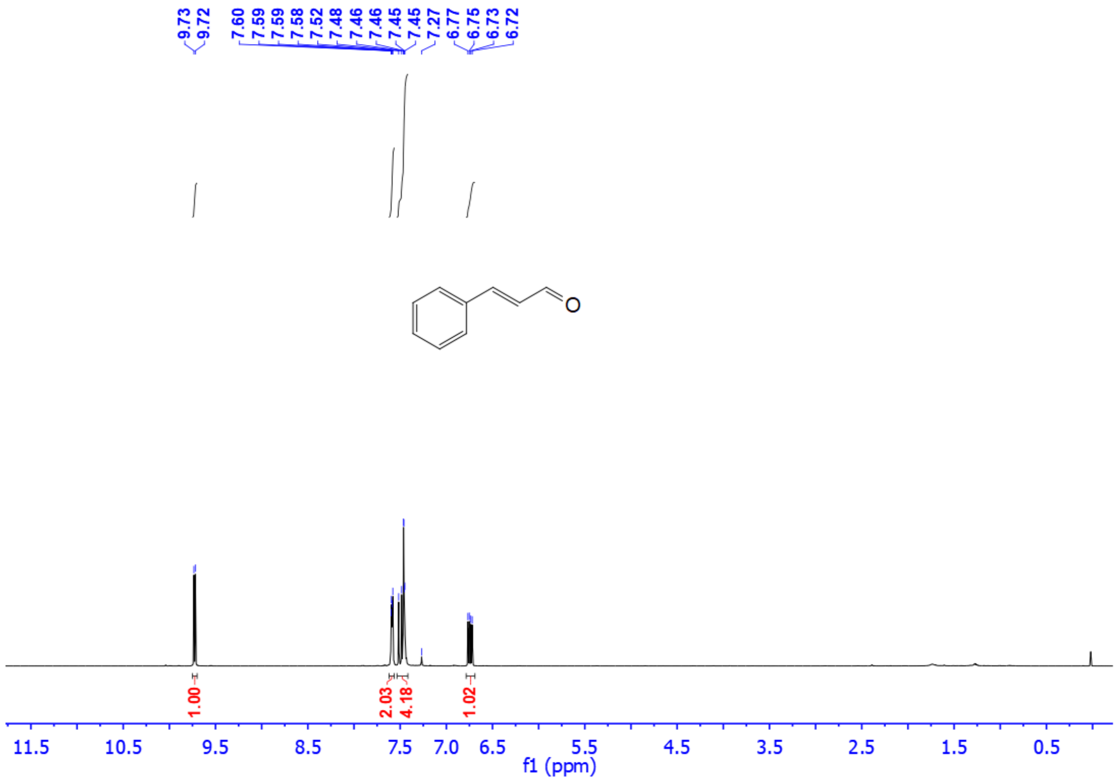

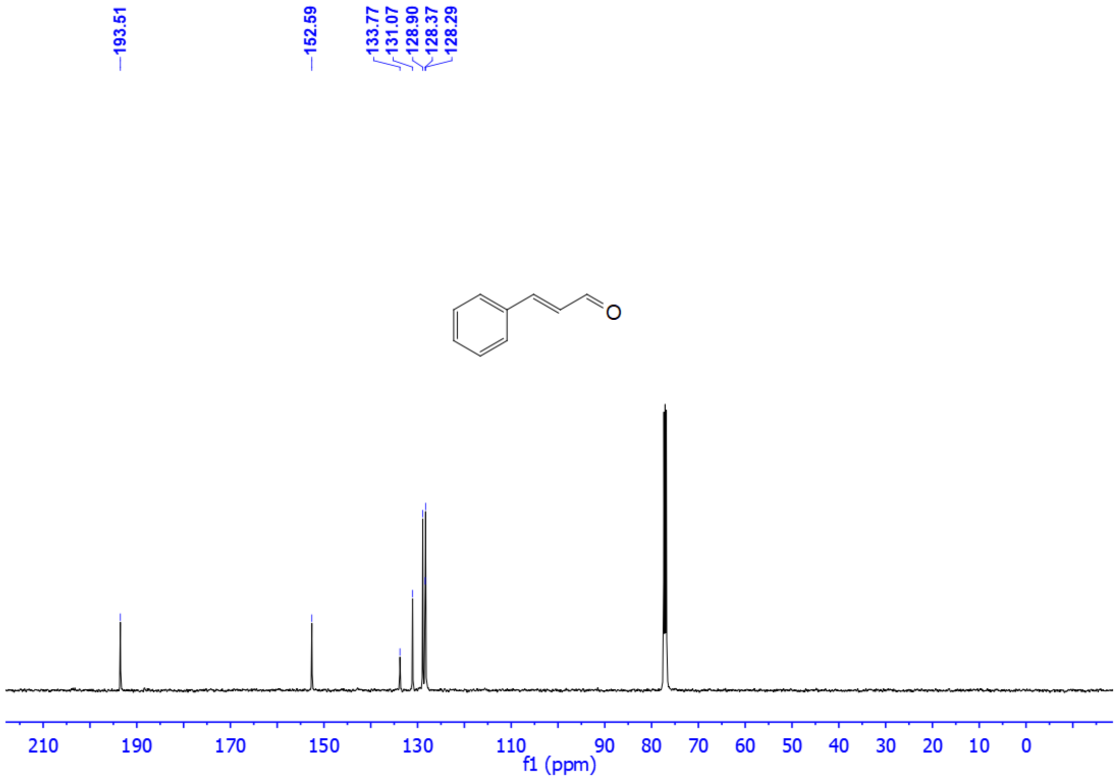

The ^{1}H and ^{13}C NMR spectra of compounds (2u)

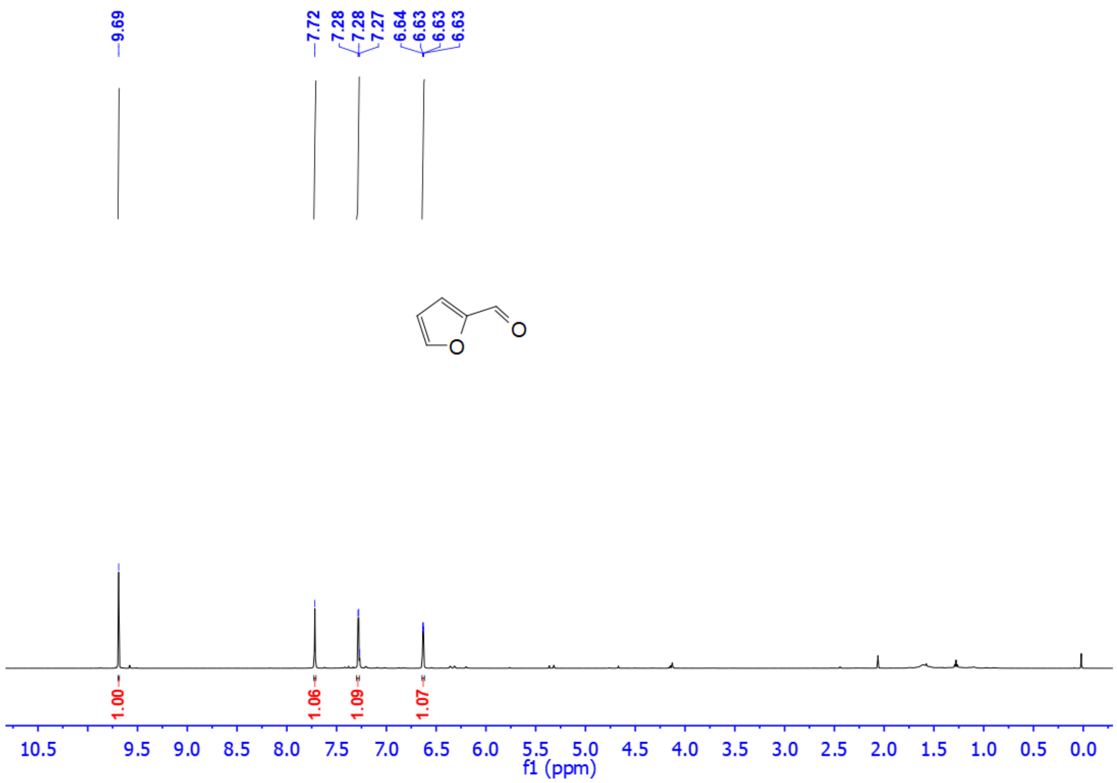

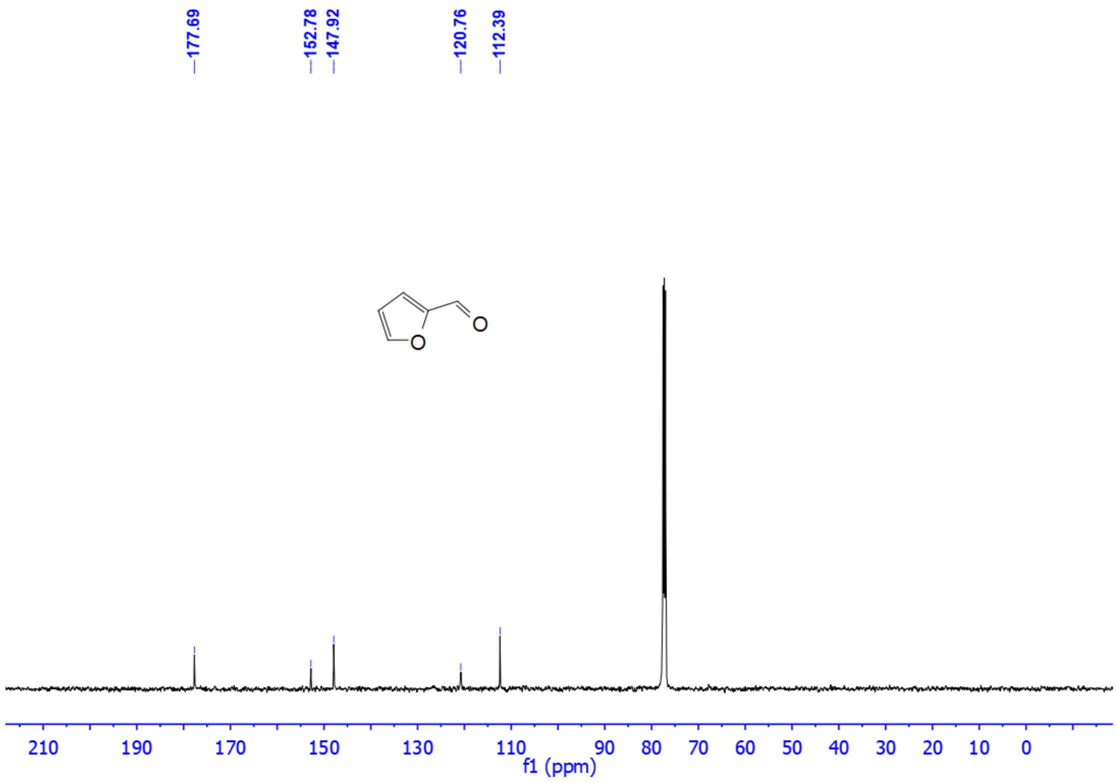

The ^{1}H and ^{13}C NMR spectra of compounds (2v)

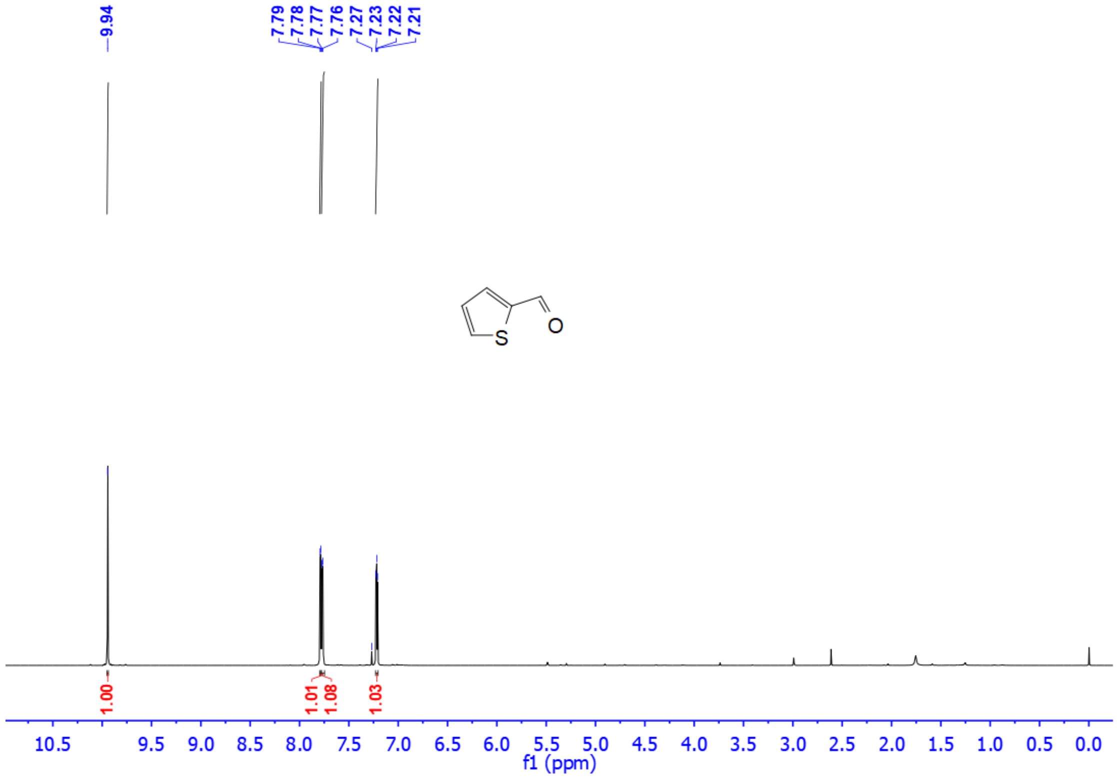

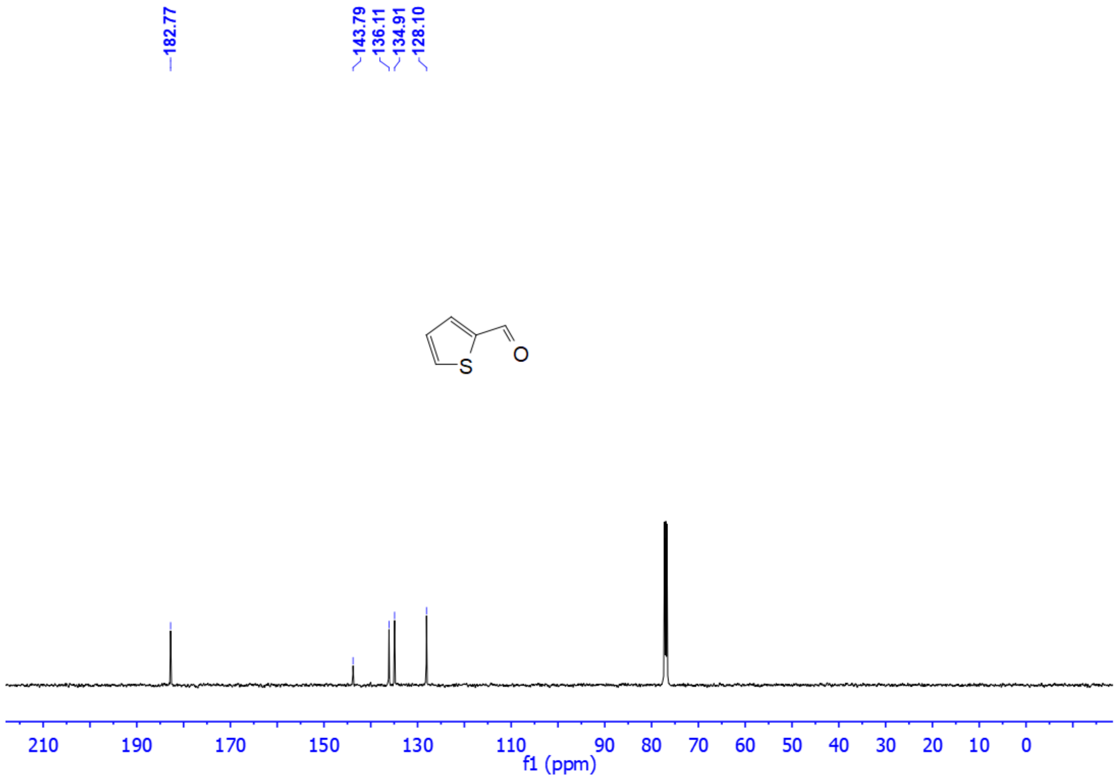

The ^{1}H and ^{13}C NMR spectra of compounds (4a)

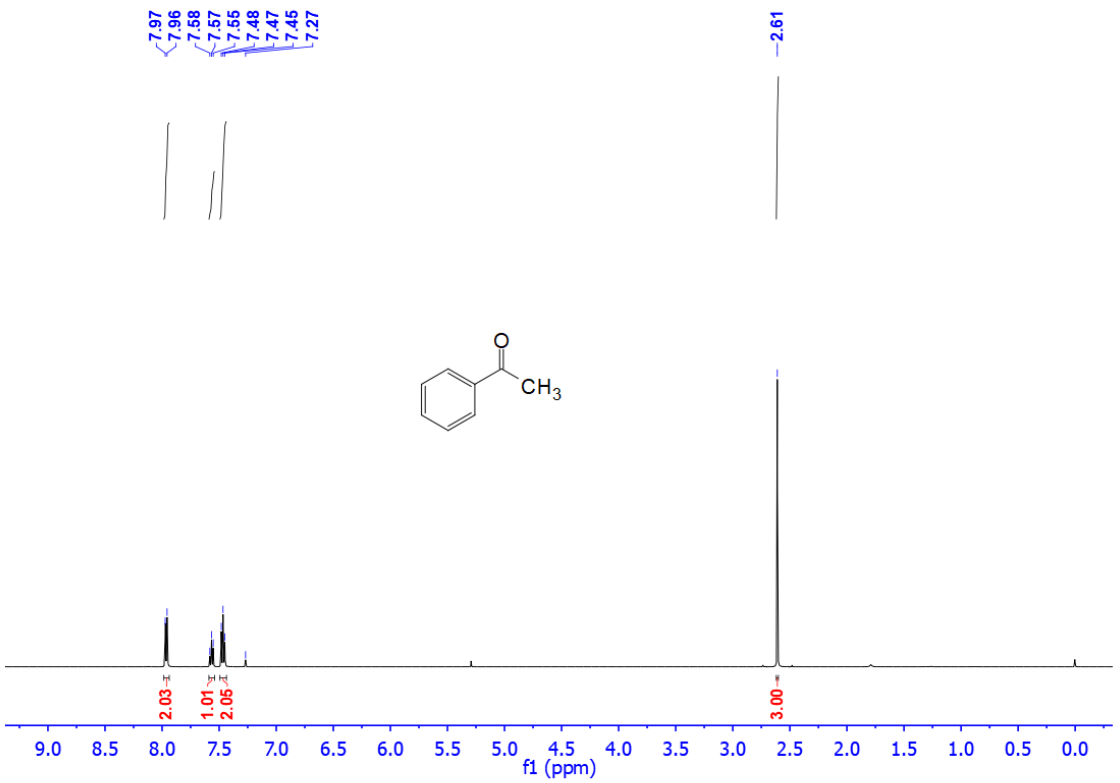

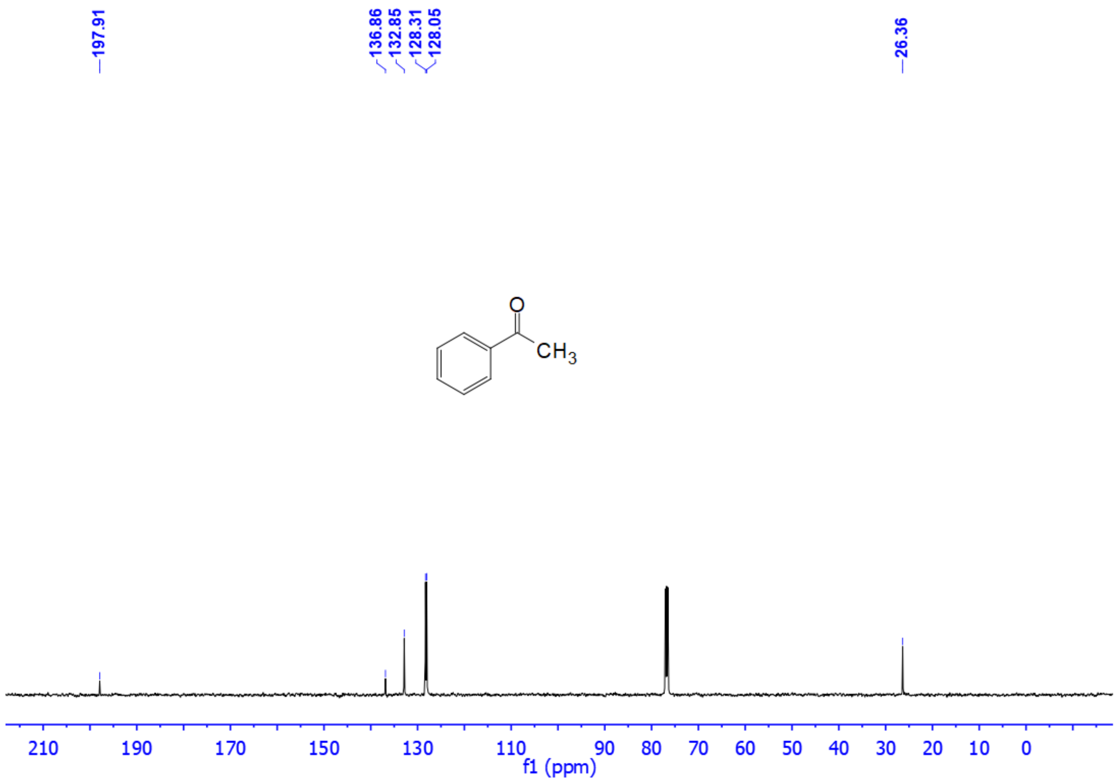

The ^{1}H and ^{13}C NMR spectra of compounds (4b)

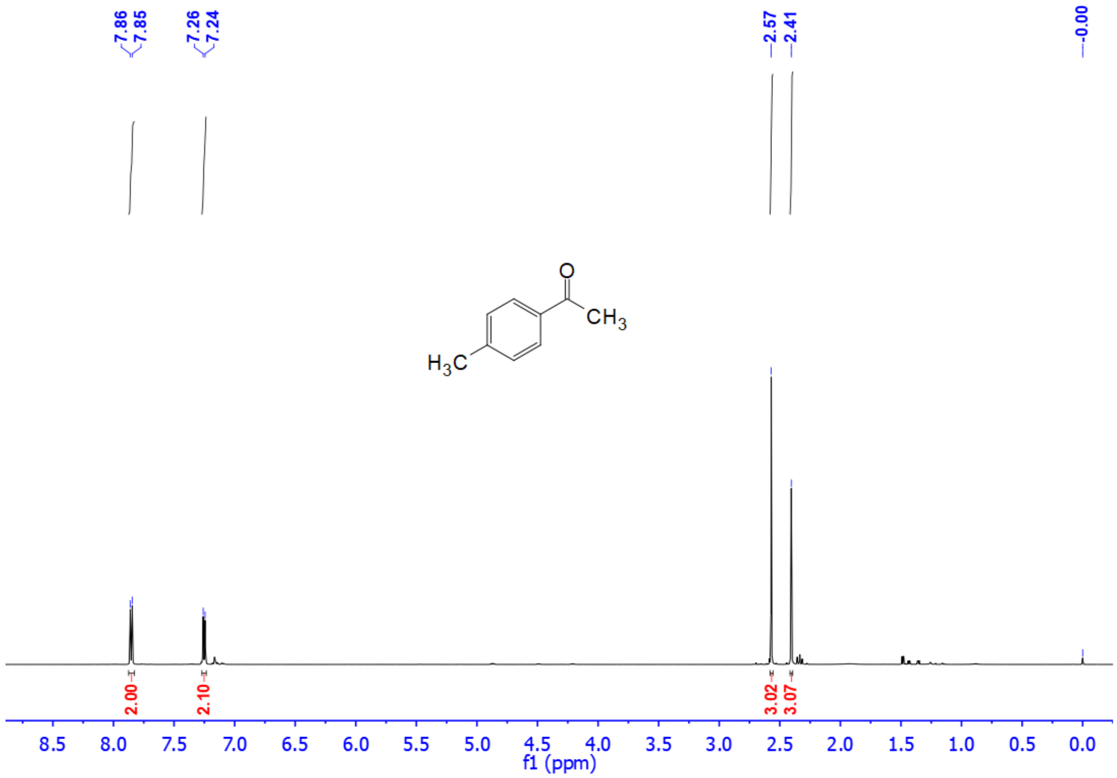

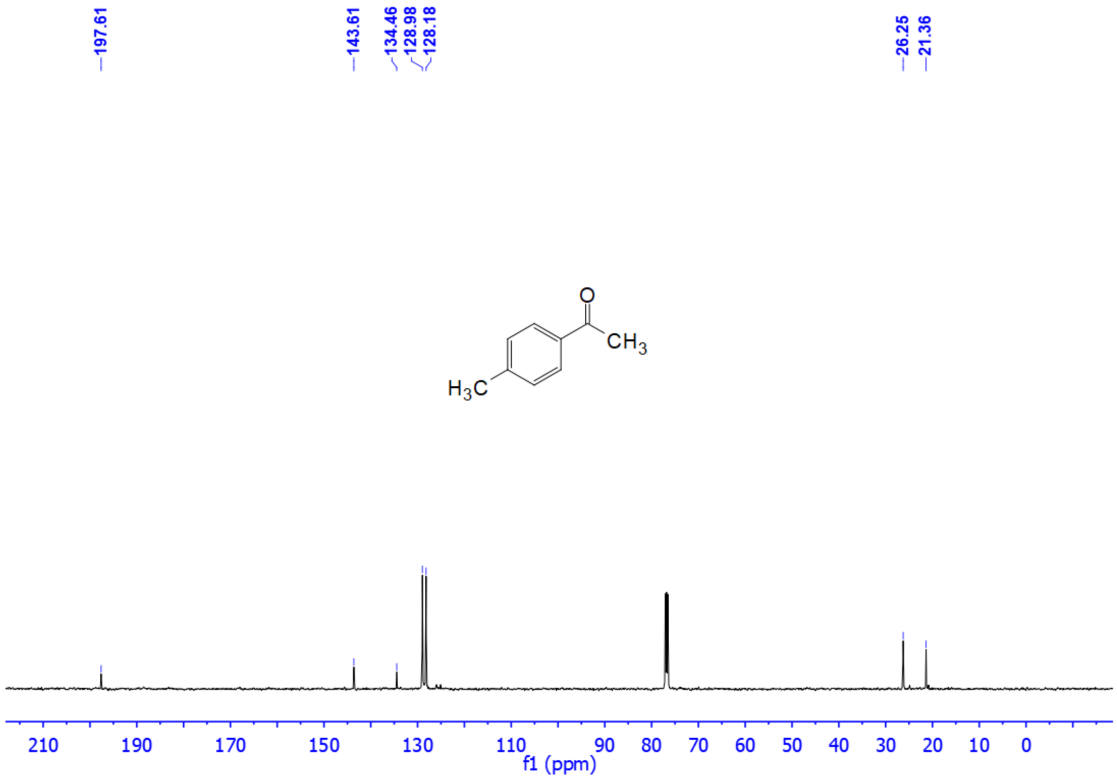

The ^{1}H and ^{13}C NMR spectra of compounds (4c)

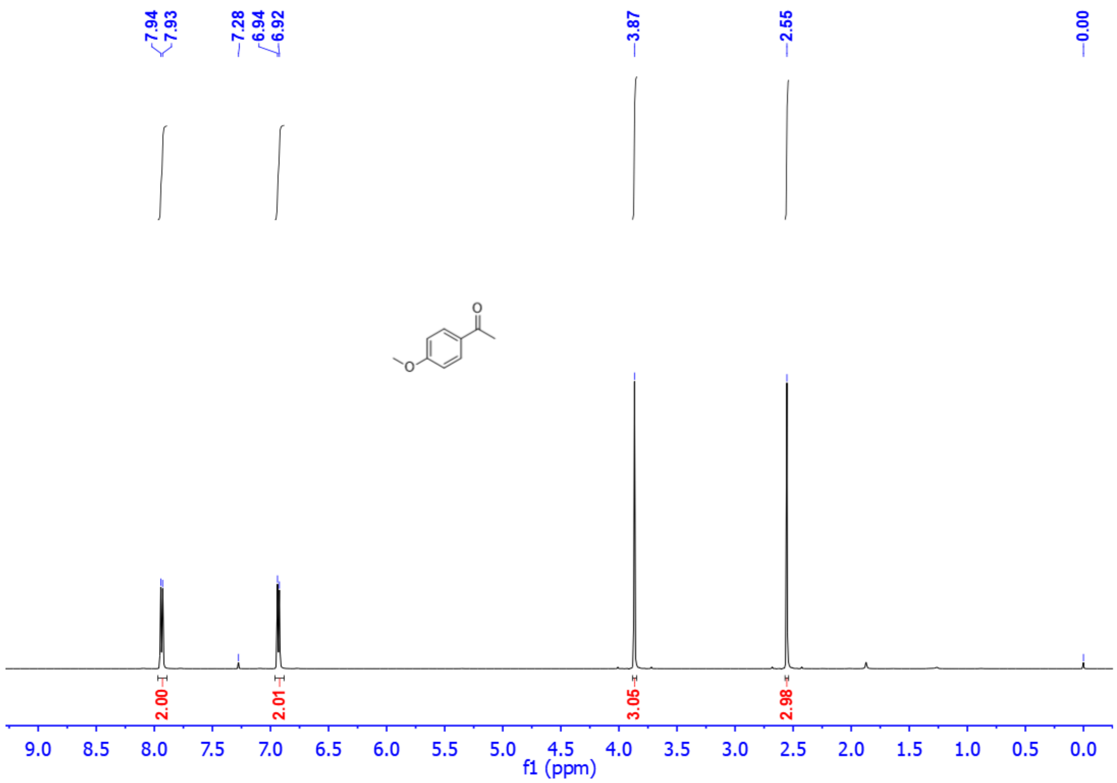

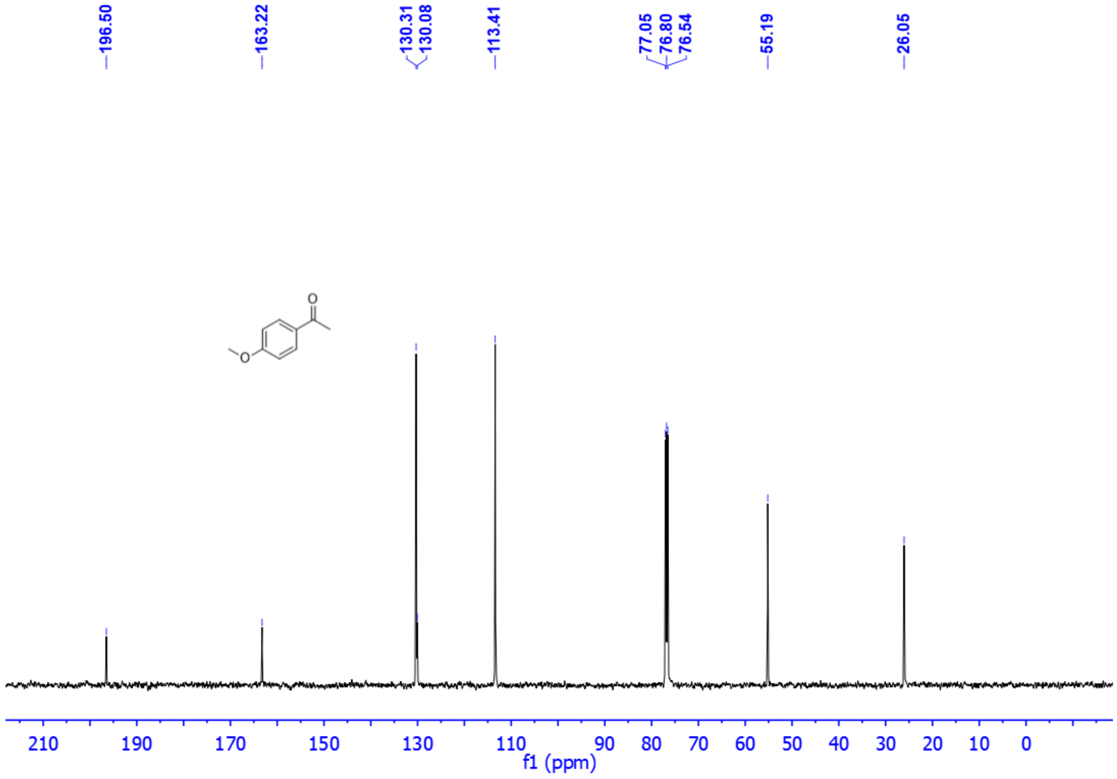

The ^{1}H and ^{13}C NMR spectra of compounds (4d)

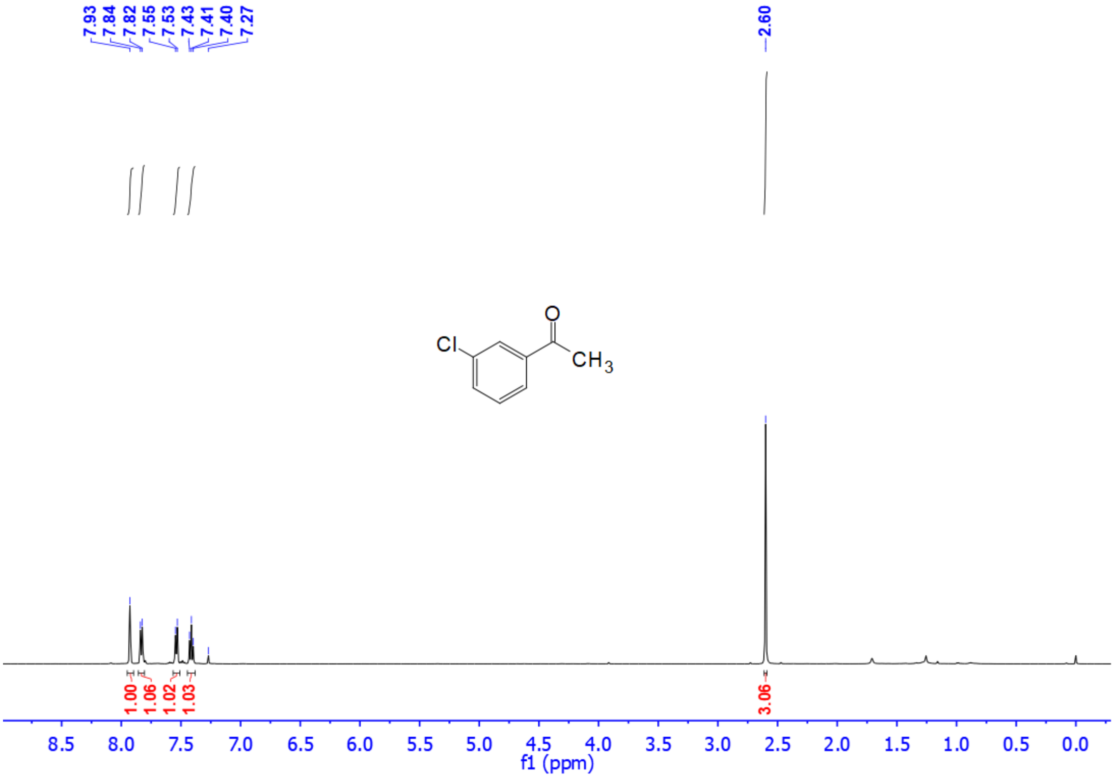

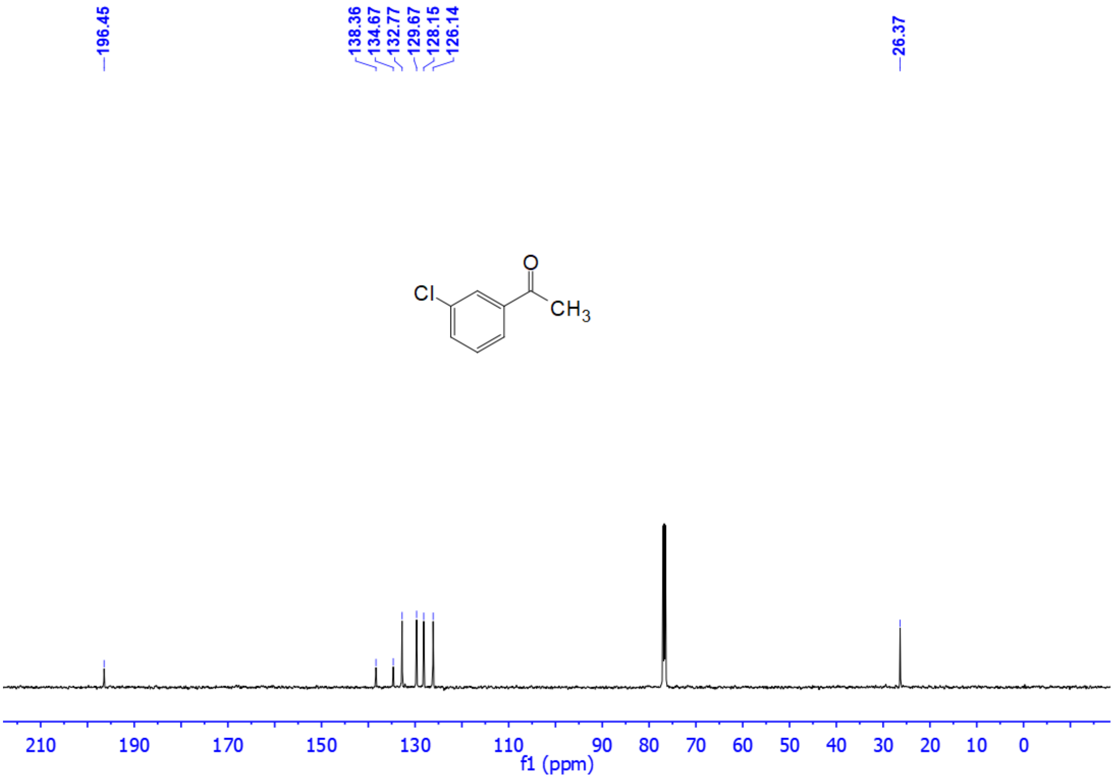

The ^{1}H and ^{13}C NMR spectra of compounds (4e)

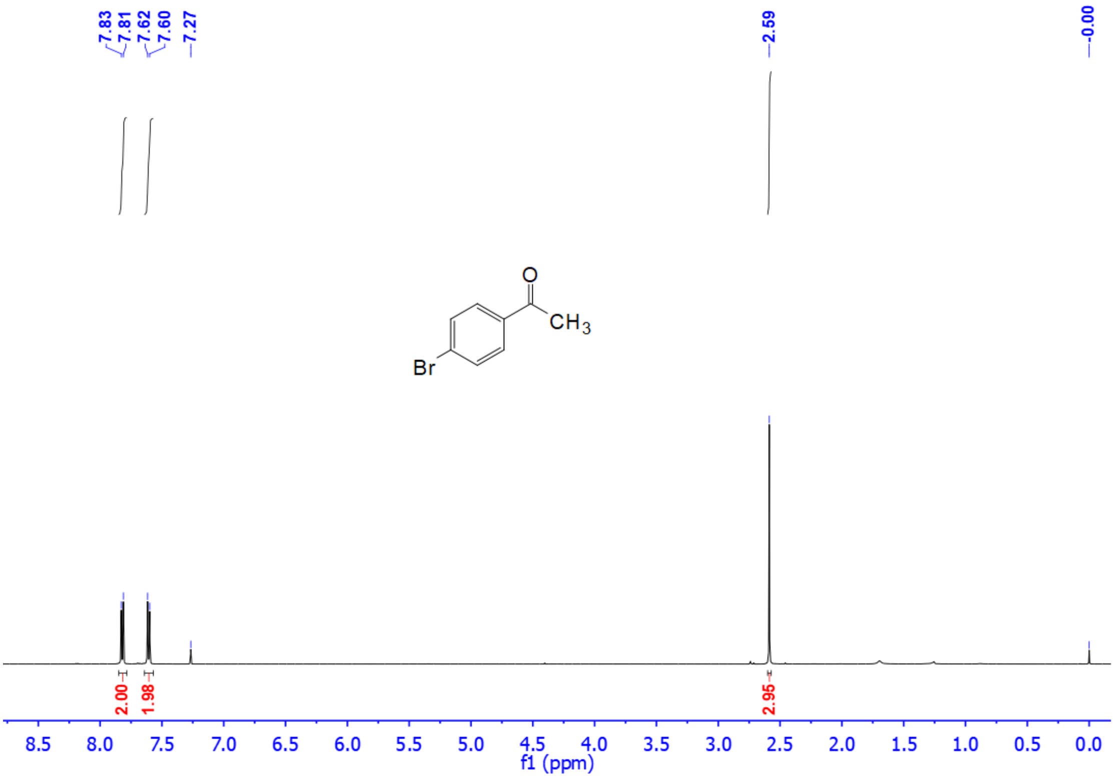

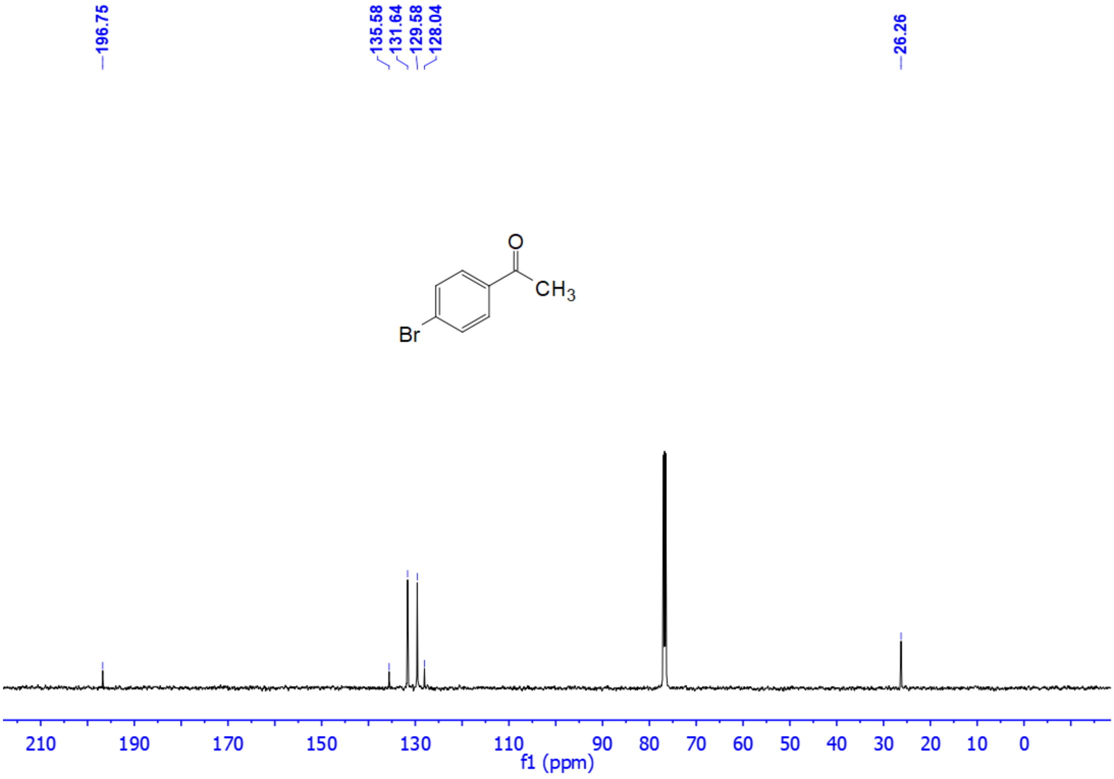

The ^{1}H and ^{13}C NMR spectra of compounds (4f)

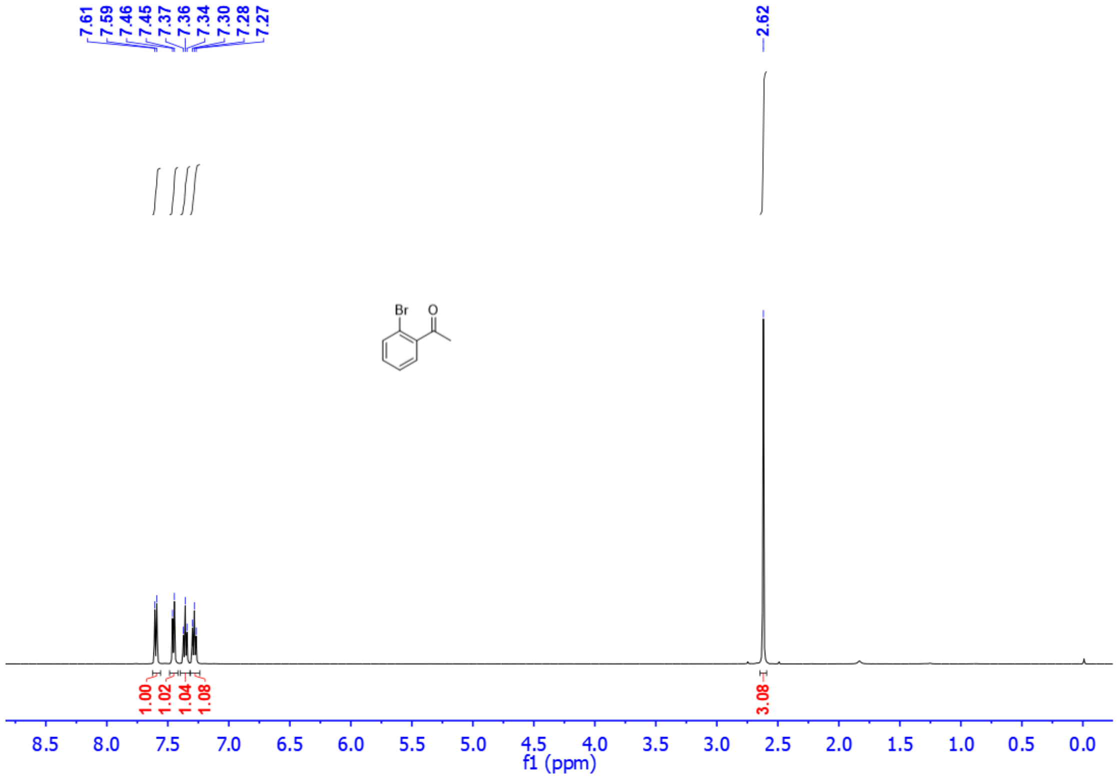

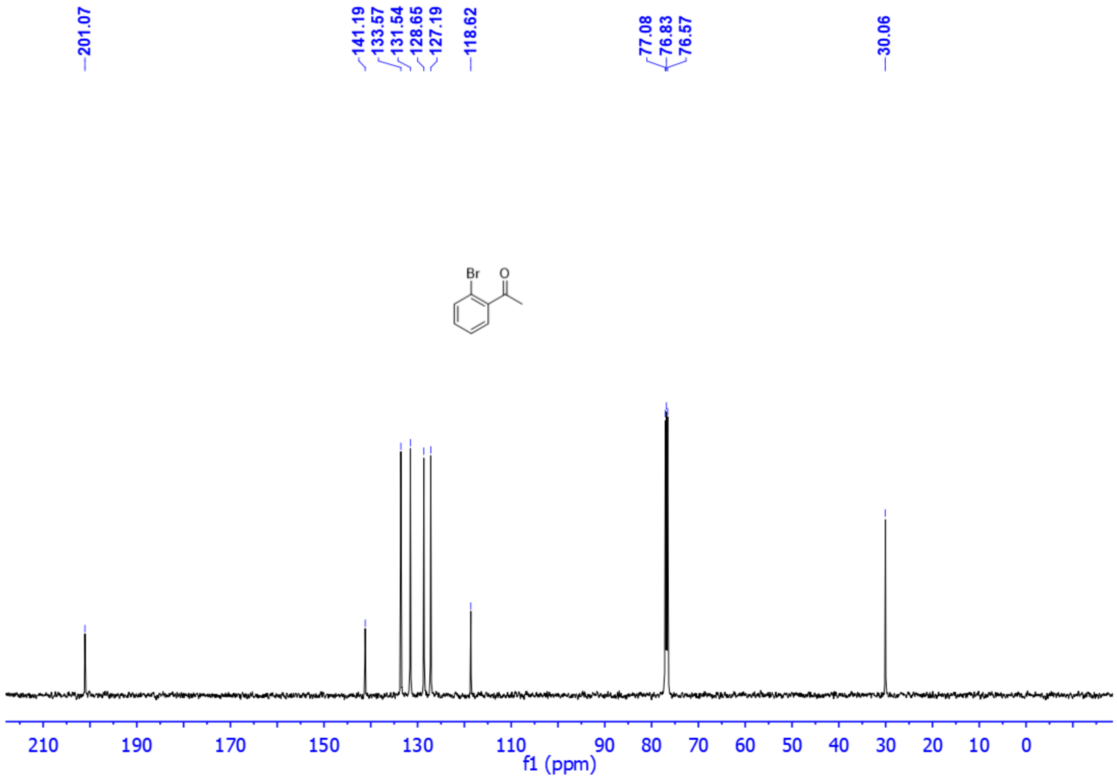

The ^{1}H and ^{13}C NMR spectra of compounds (4g)

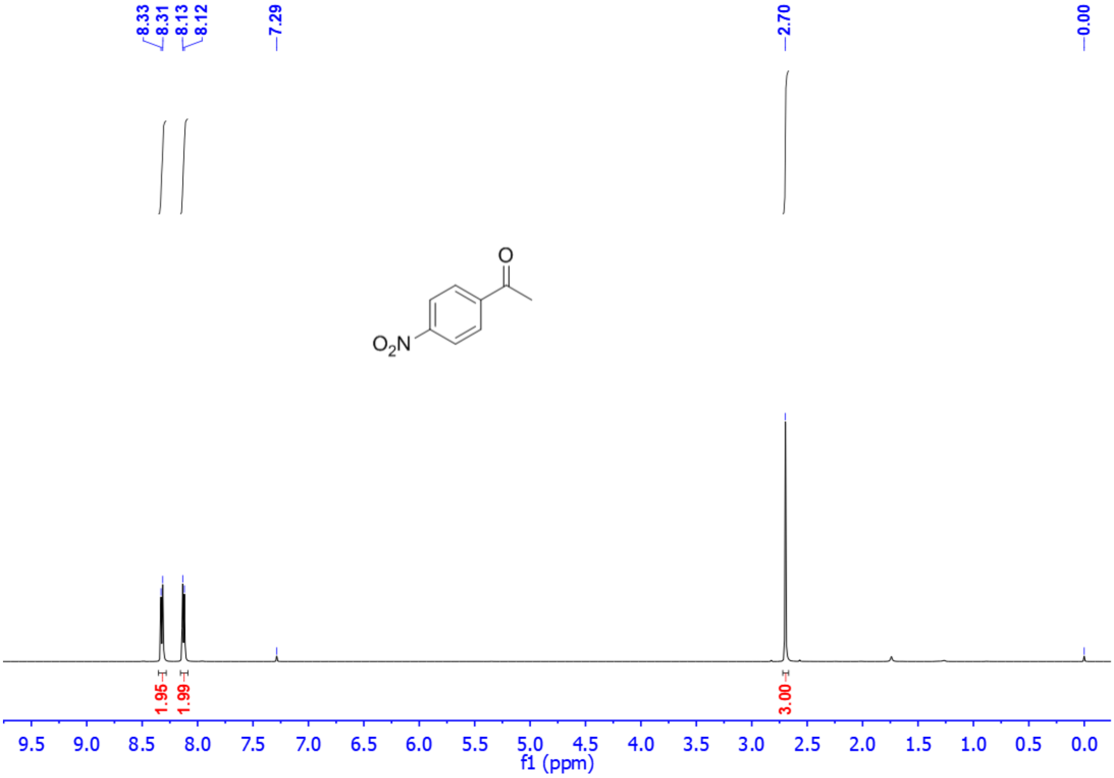

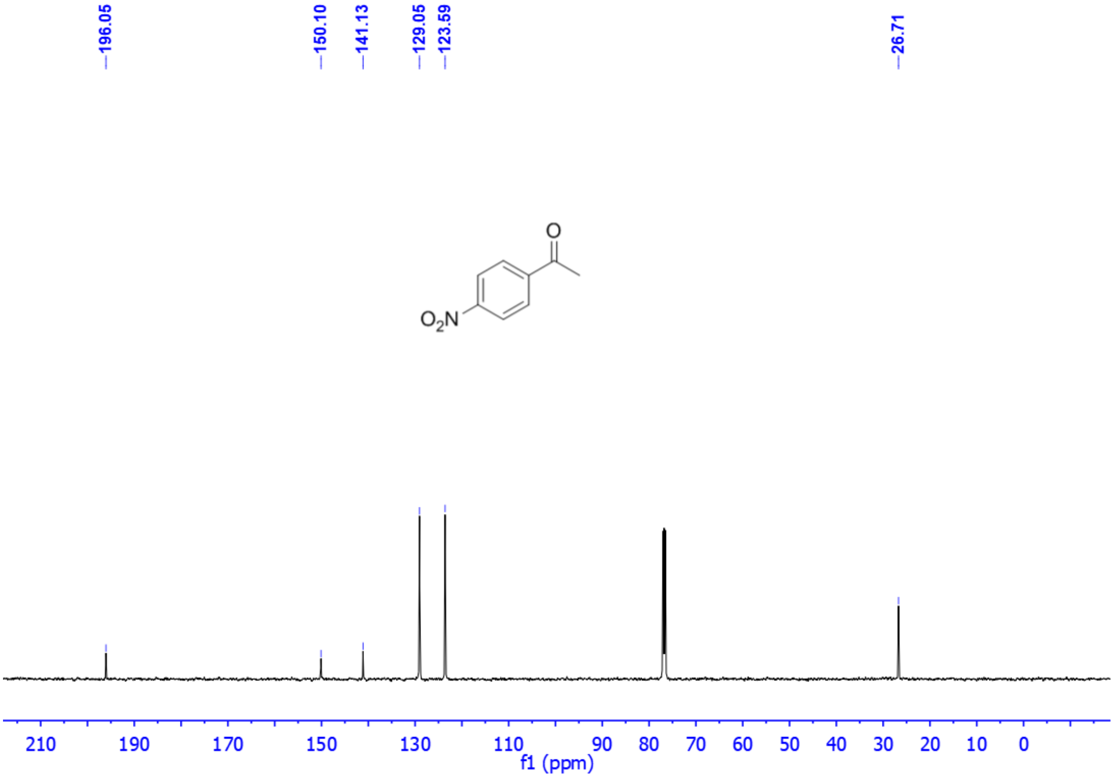

The ^{1}H and ^{13}C NMR spectra of compounds (4h)

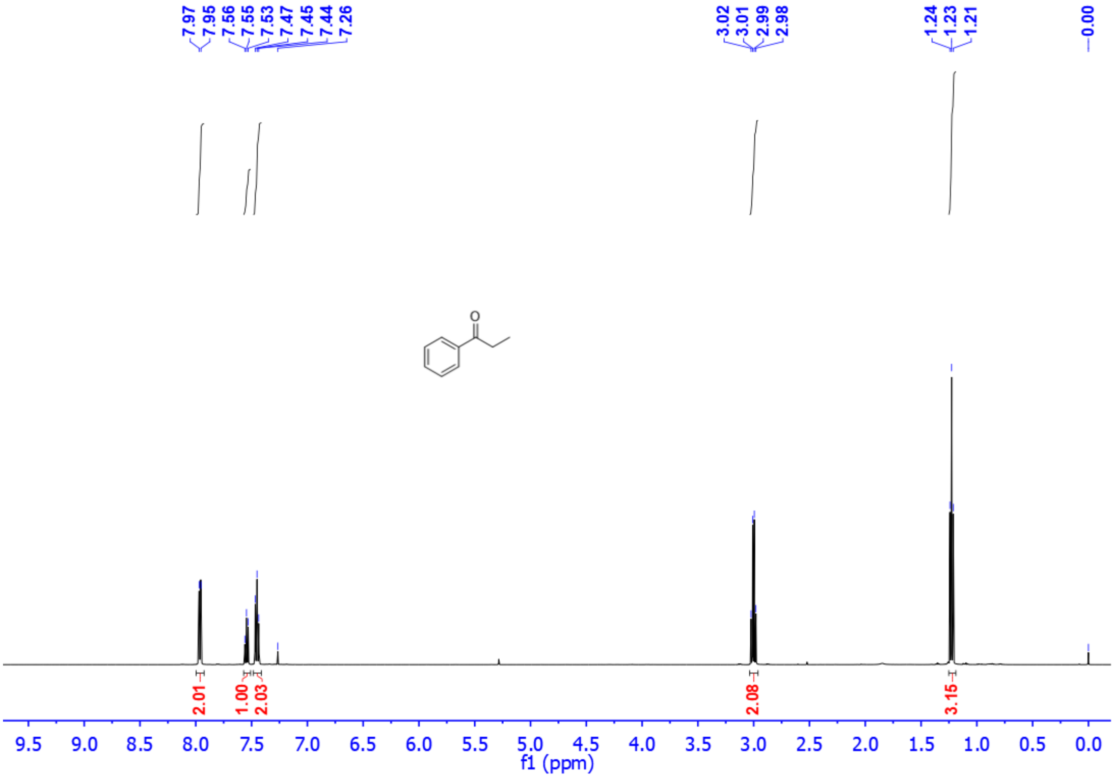

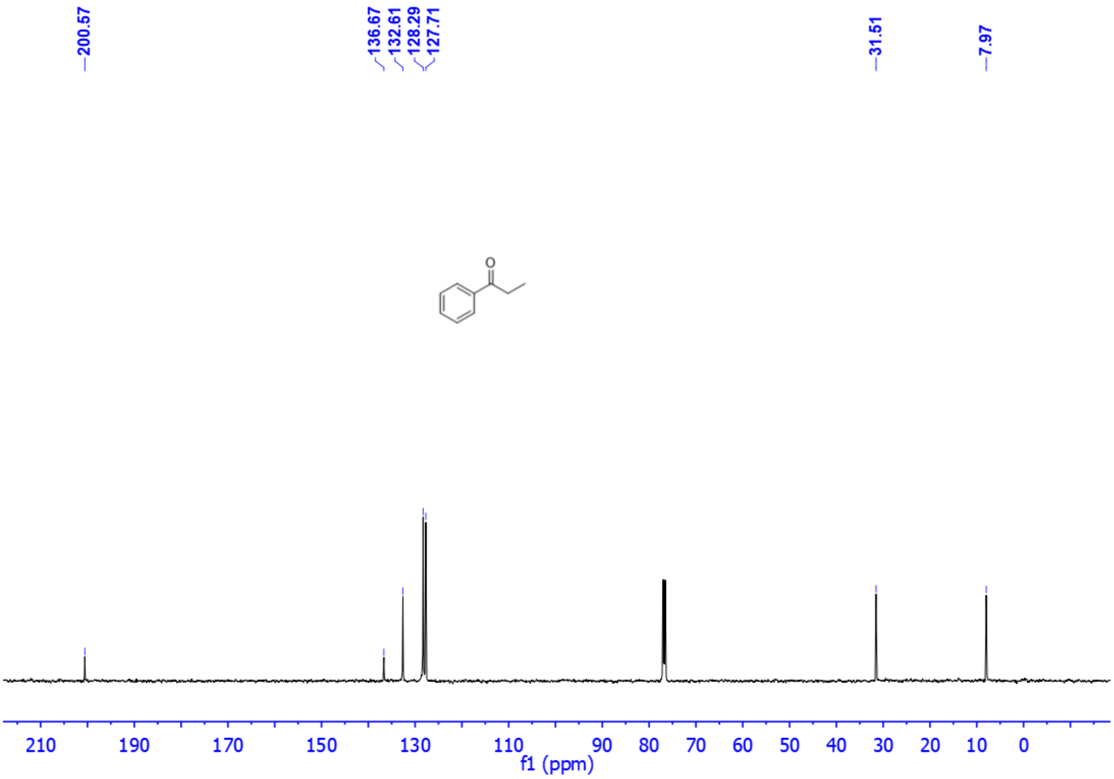

The ^{1}H and ^{13}C NMR spectra of compounds (4i)

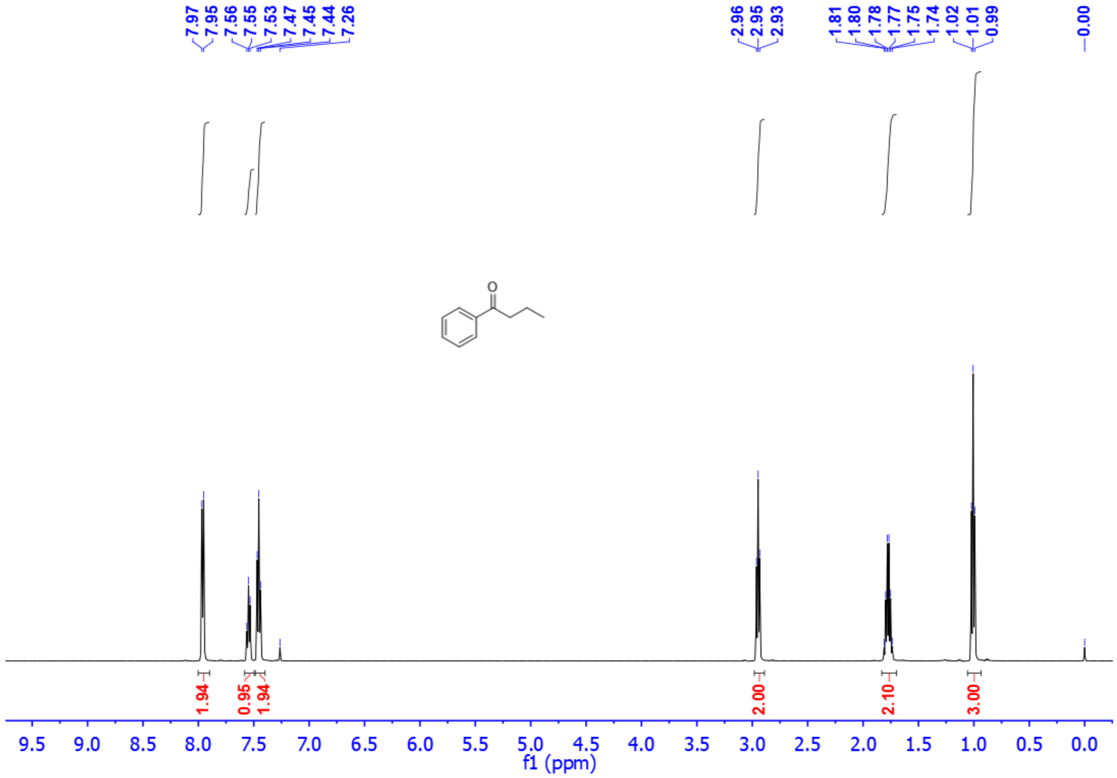

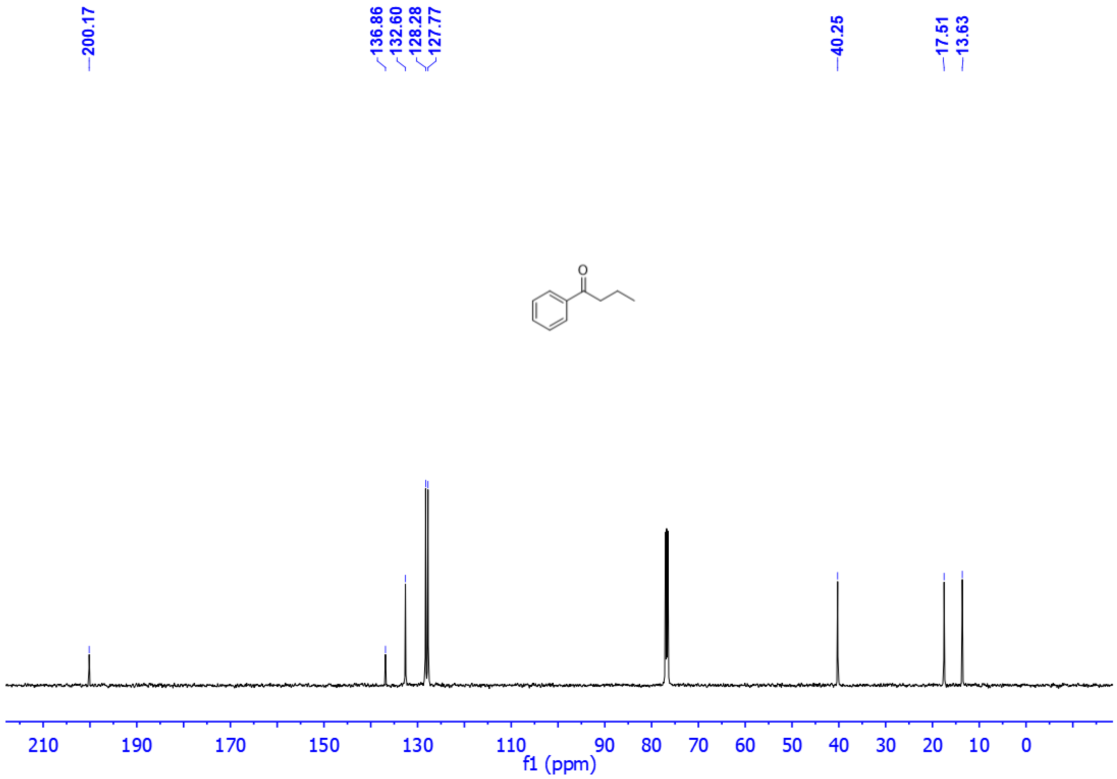

The ^{1}H and ^{13}C NMR spectra of compounds (4j)

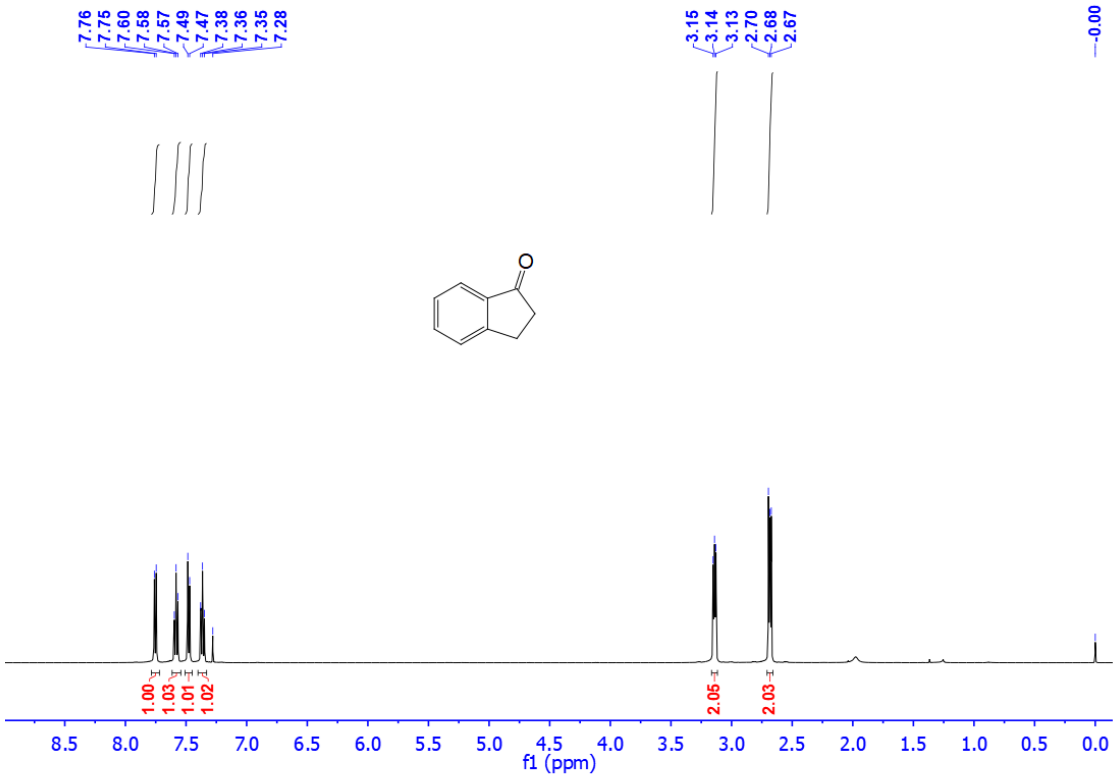

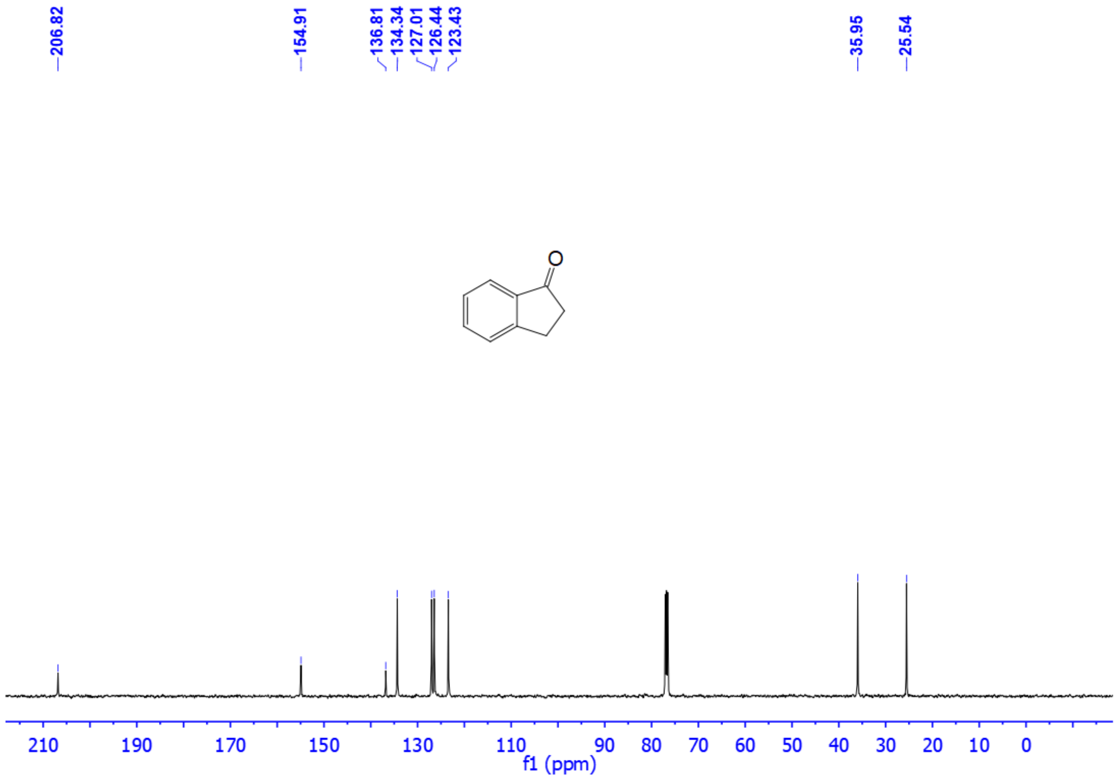

The ^{1}H and ^{13}C NMR spectra of compounds (4k)

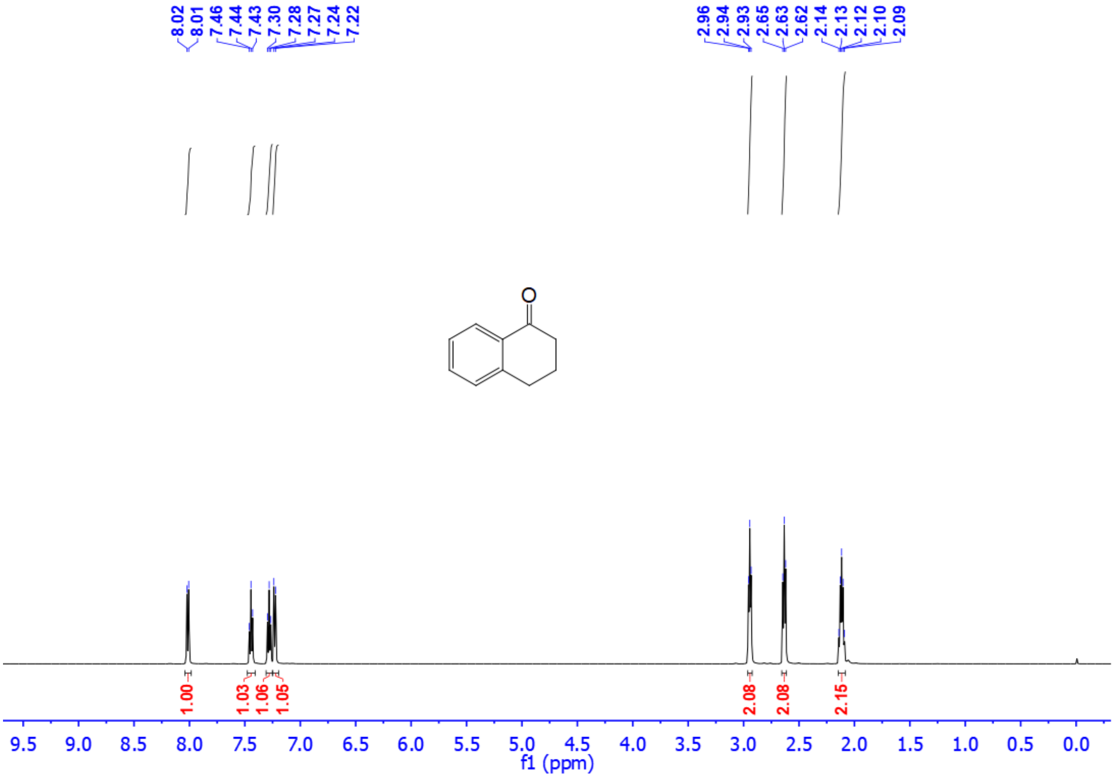

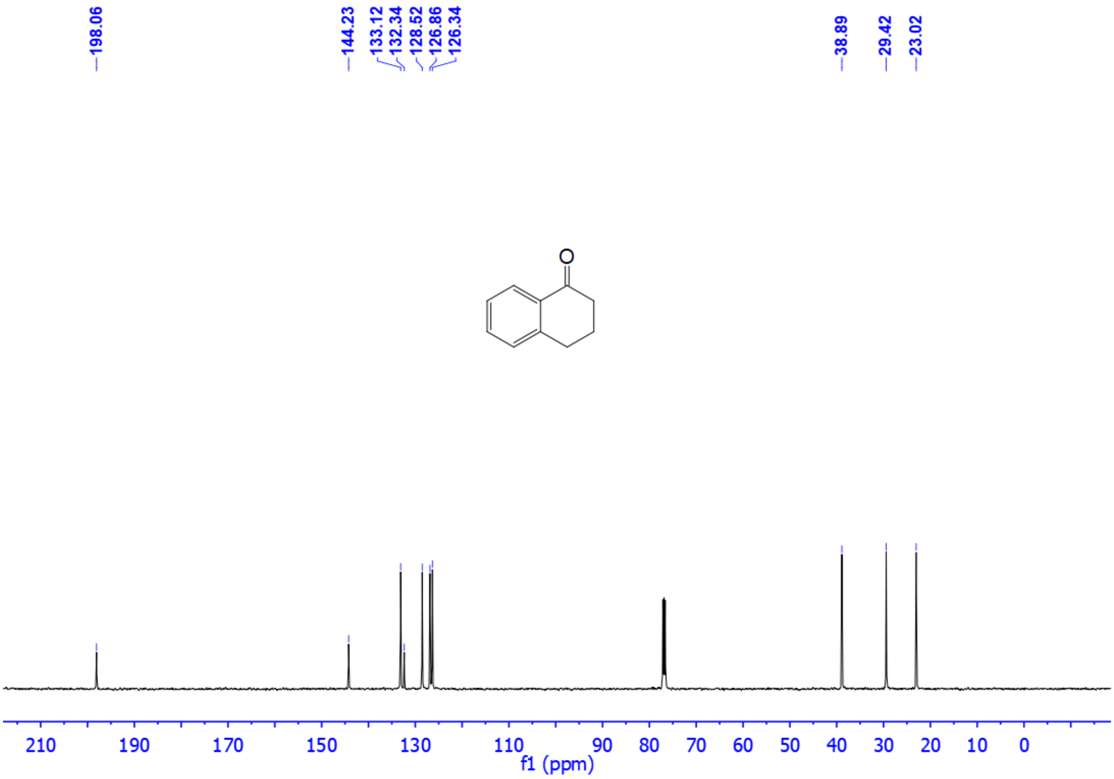

The ^{1}H and ^{13}C NMR spectra of compounds (4l)

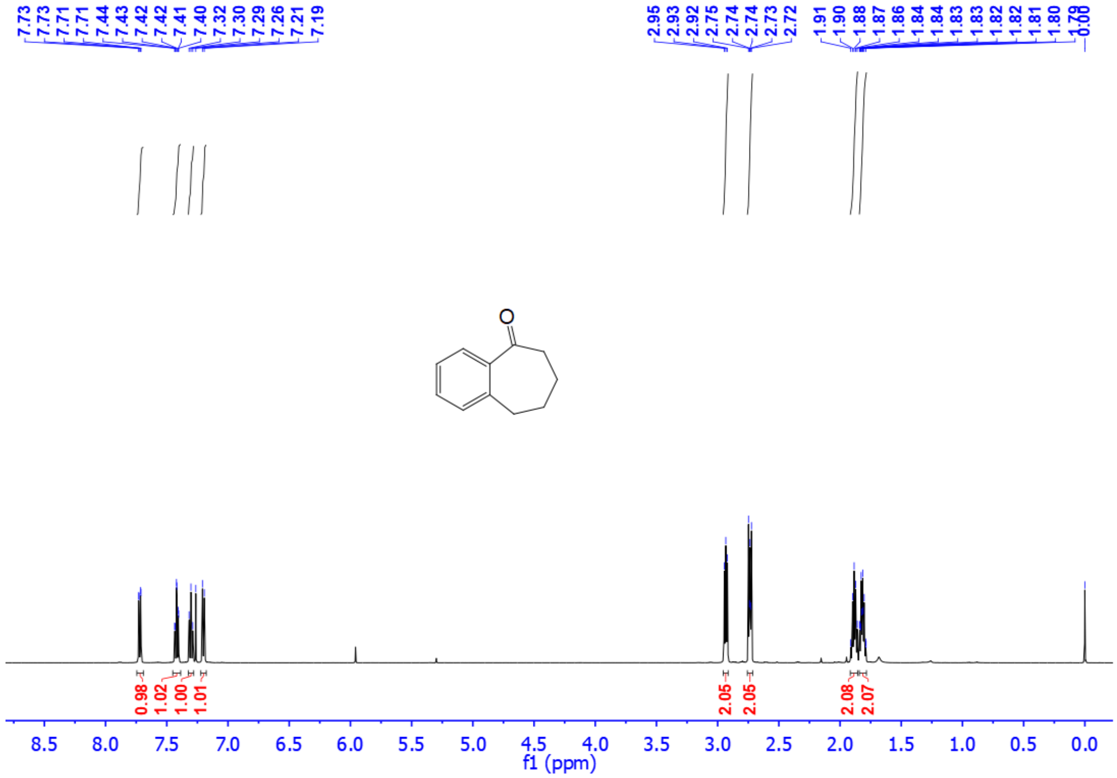

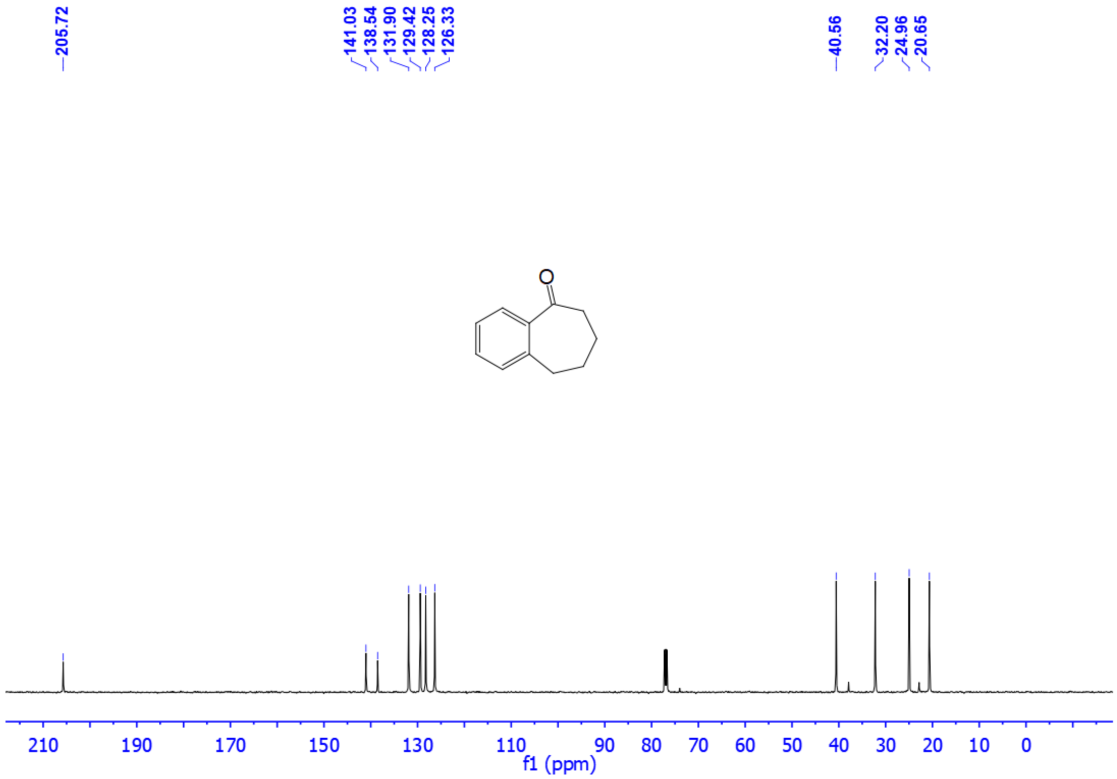

The ^{1}H and ^{13}C NMR spectra of compounds (4m)

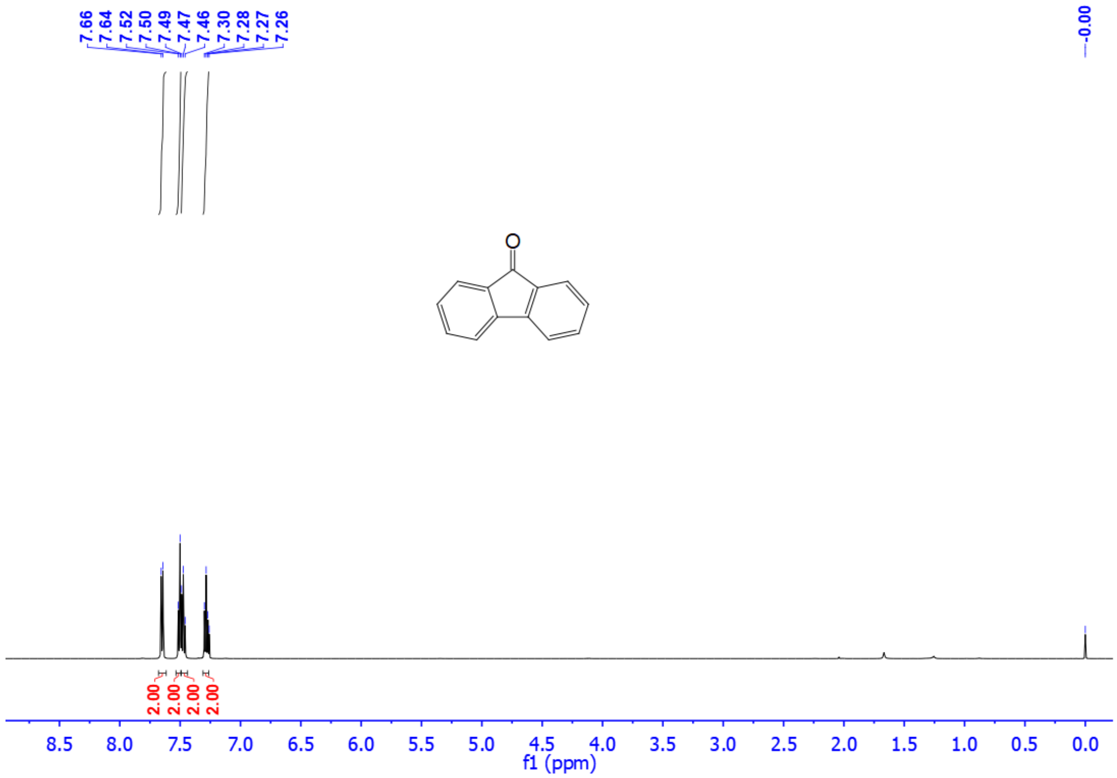

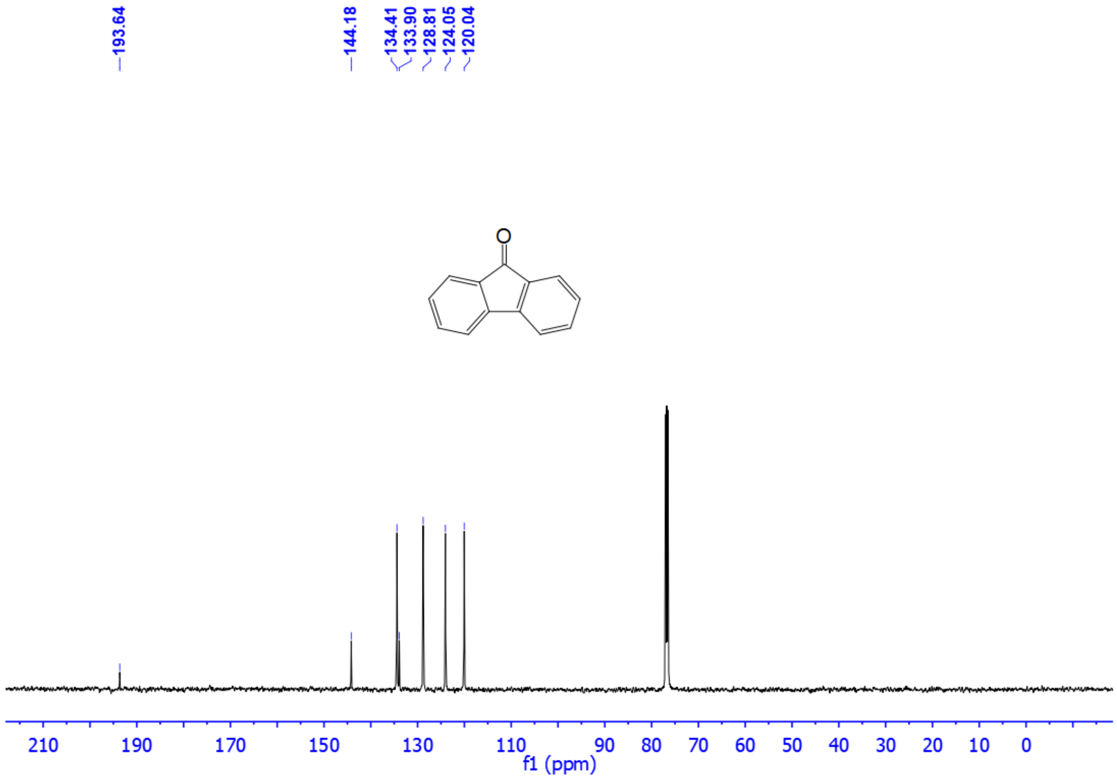

The ^{1}H and ^{13}C NMR spectra of compounds (4n)

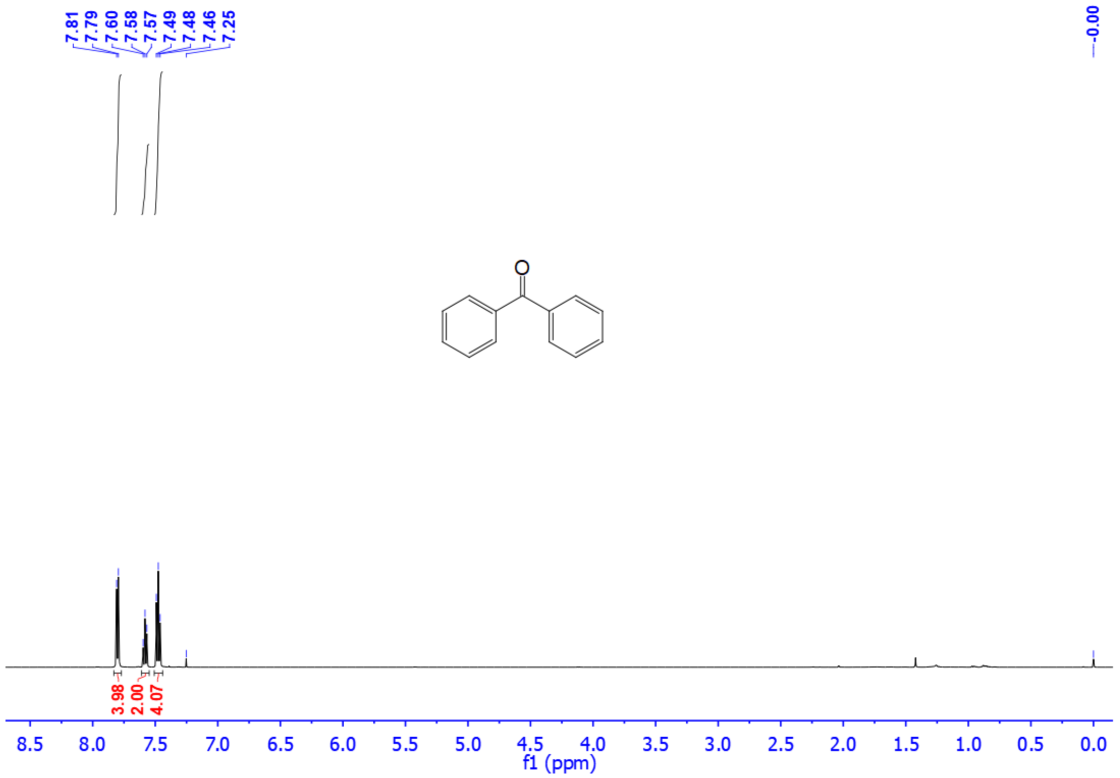

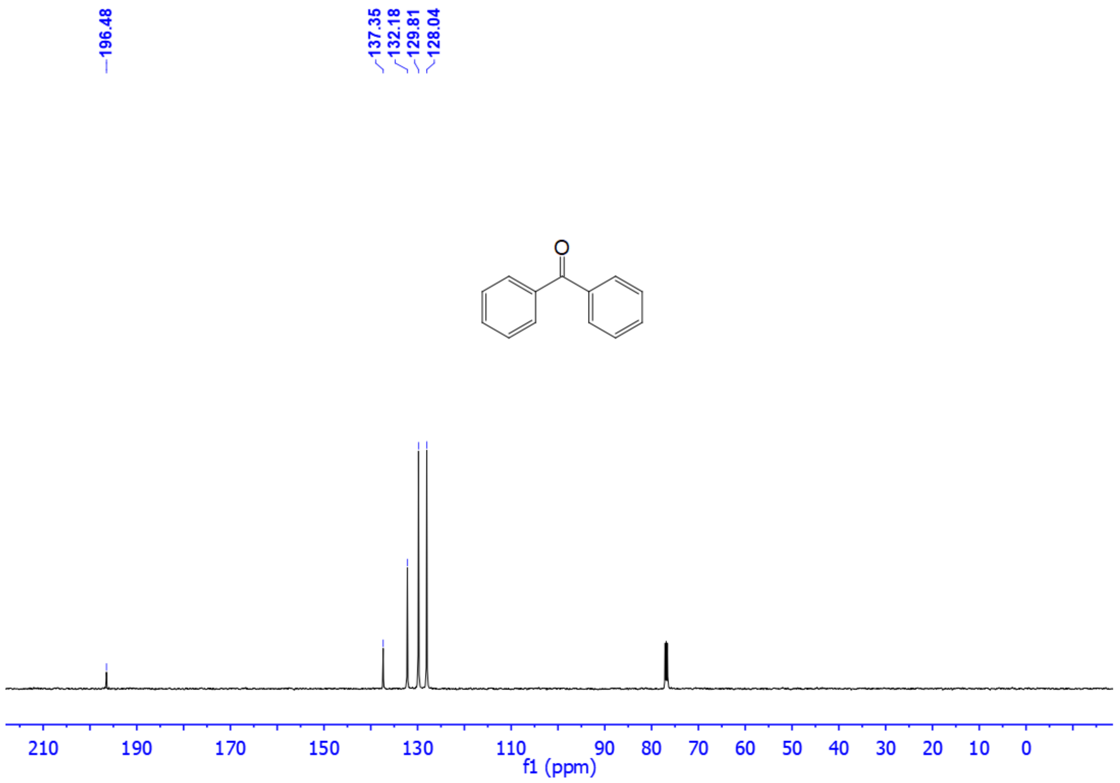

The ^{1}H and ^{13}C NMR spectra of compounds (4o)

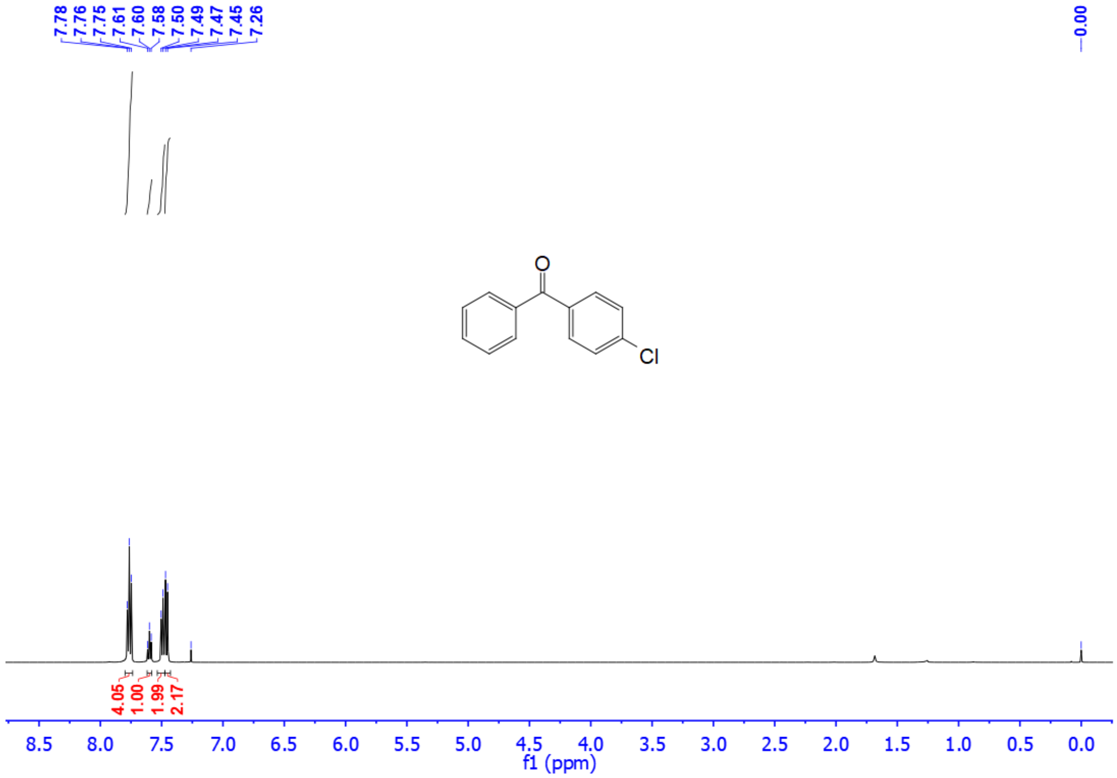

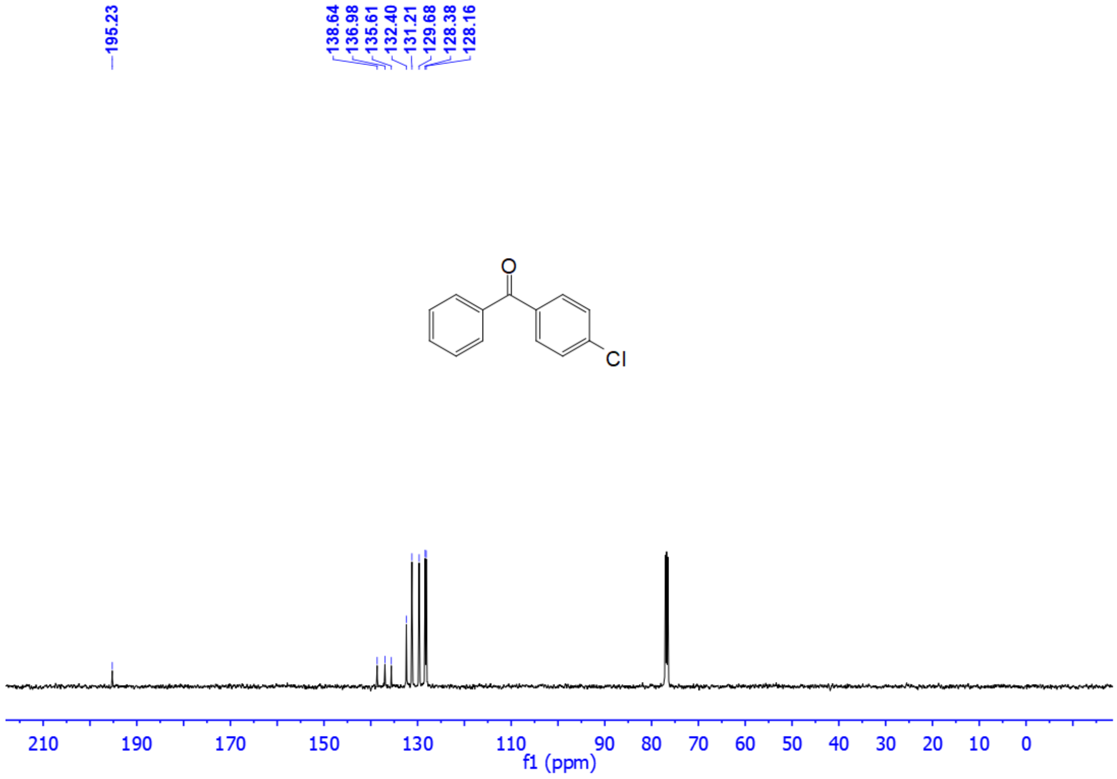

The ^{1}H and ^{13}C NMR spectra of compounds (4p)

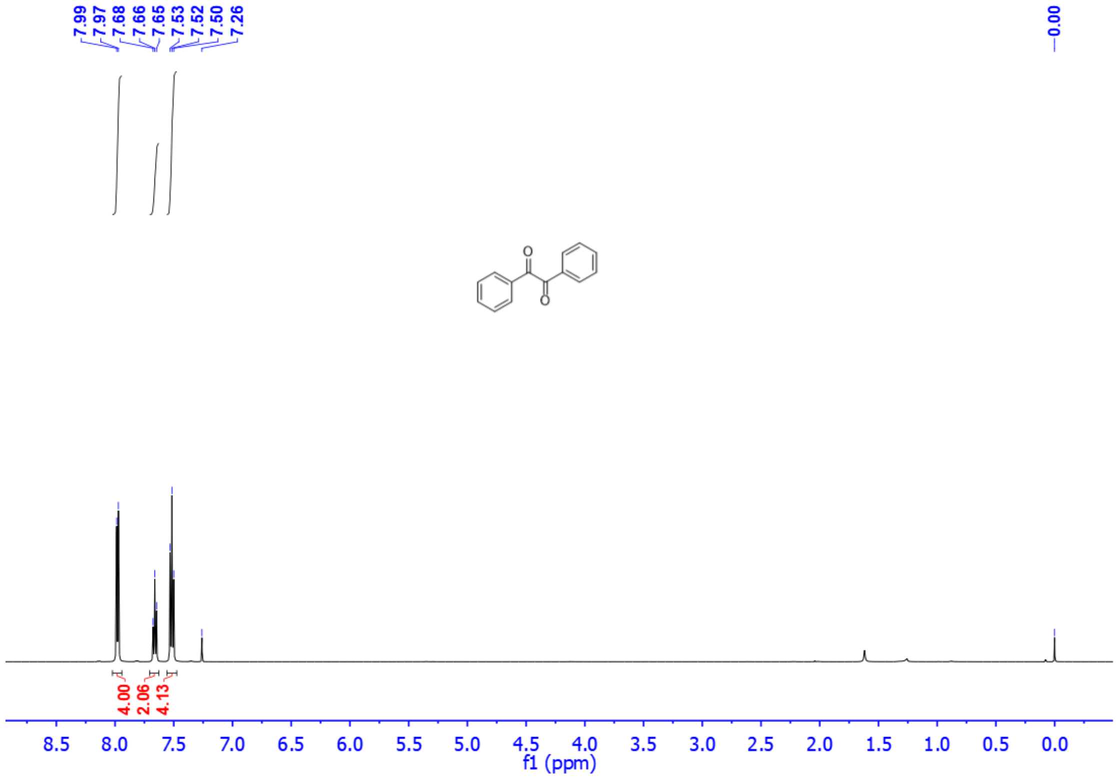

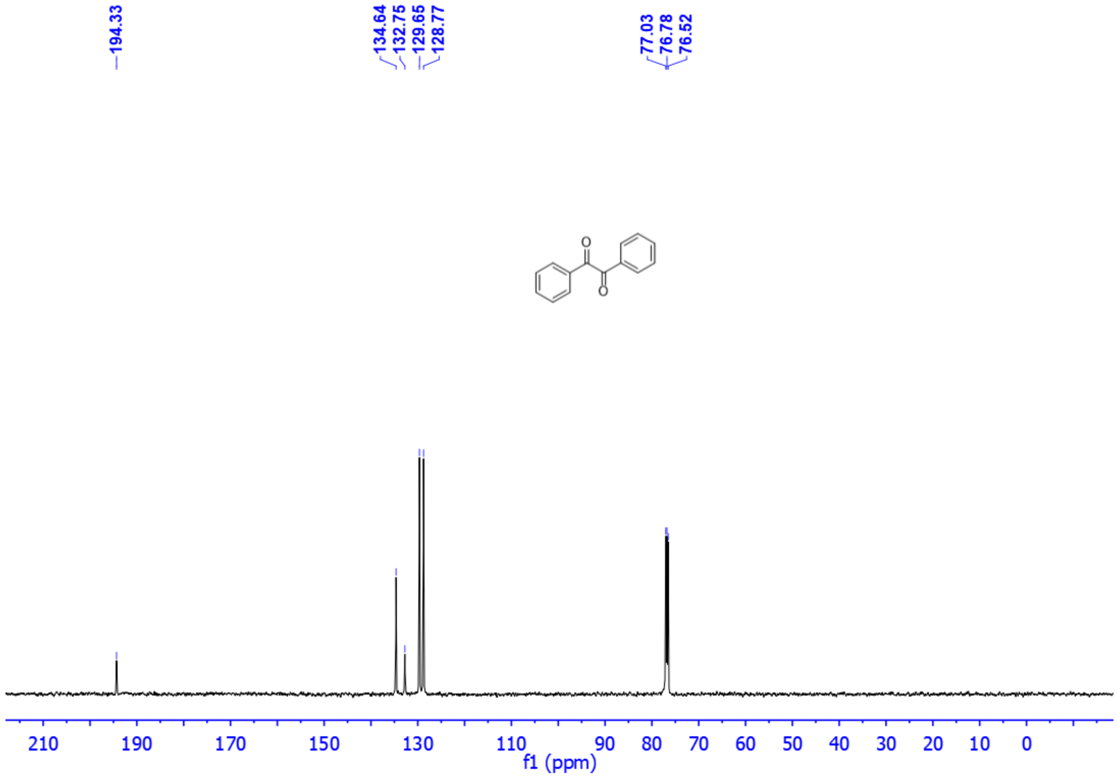

The ^{1}H and ^{13}C NMR spectra of compounds (4q)

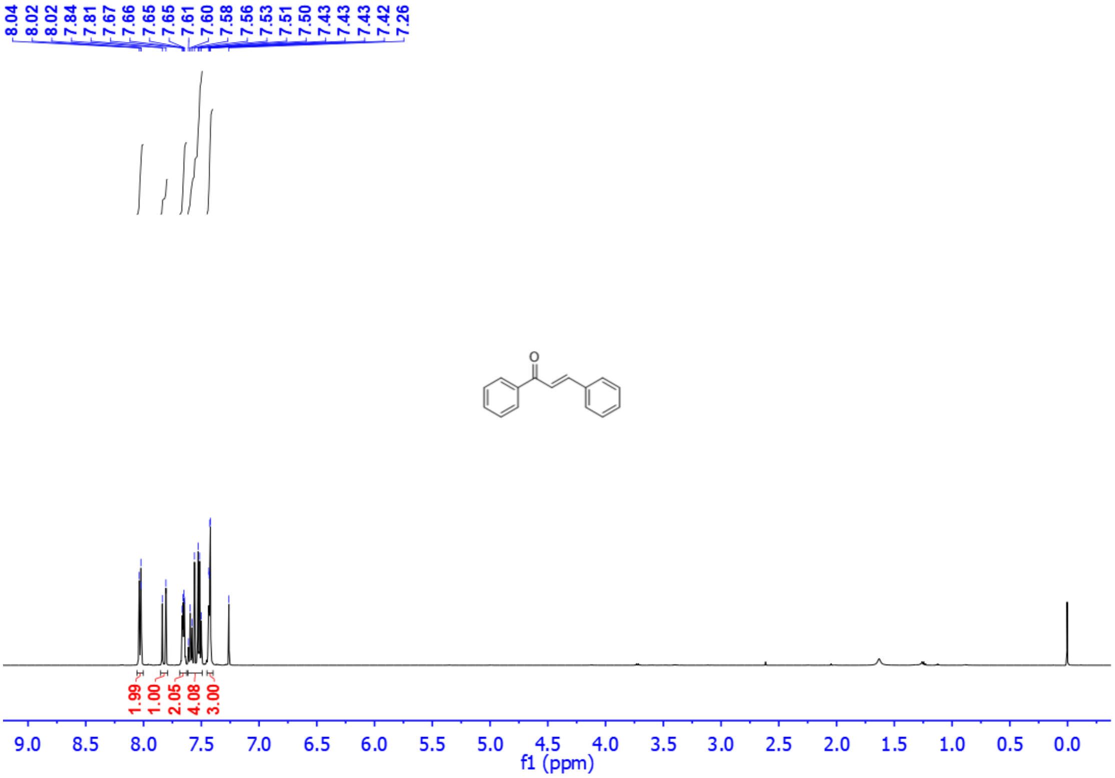

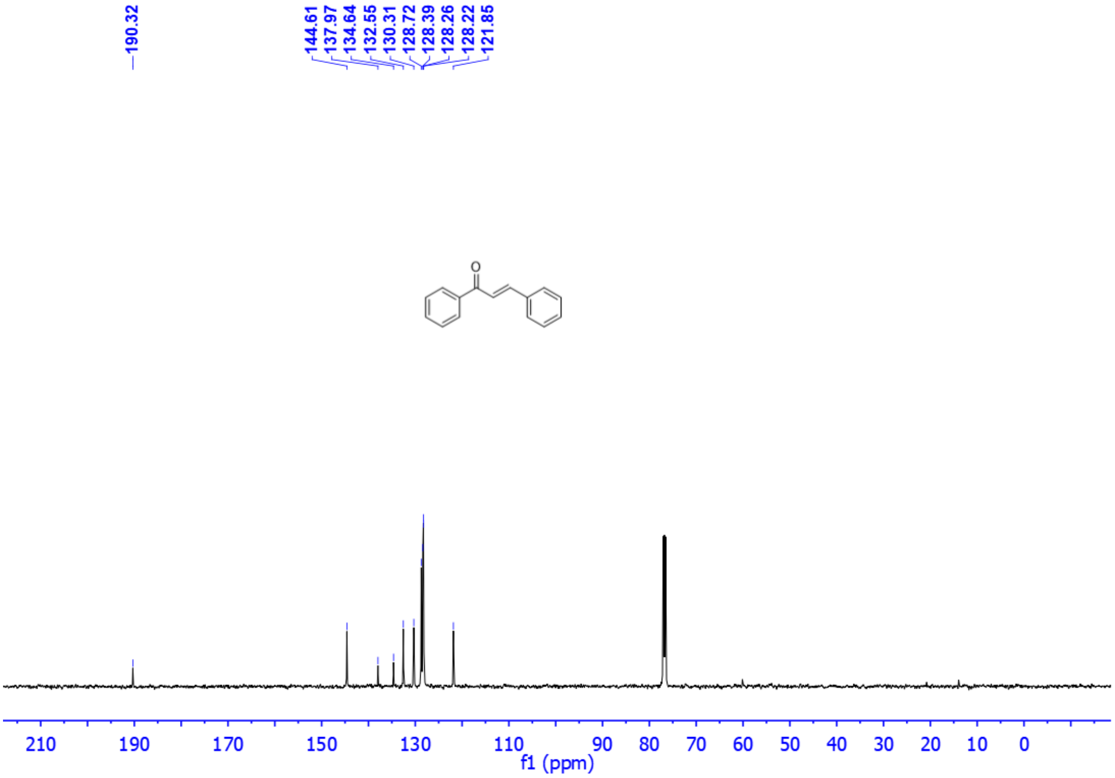

The ^{1}H and ^{13}C NMR spectra of compounds (4r)

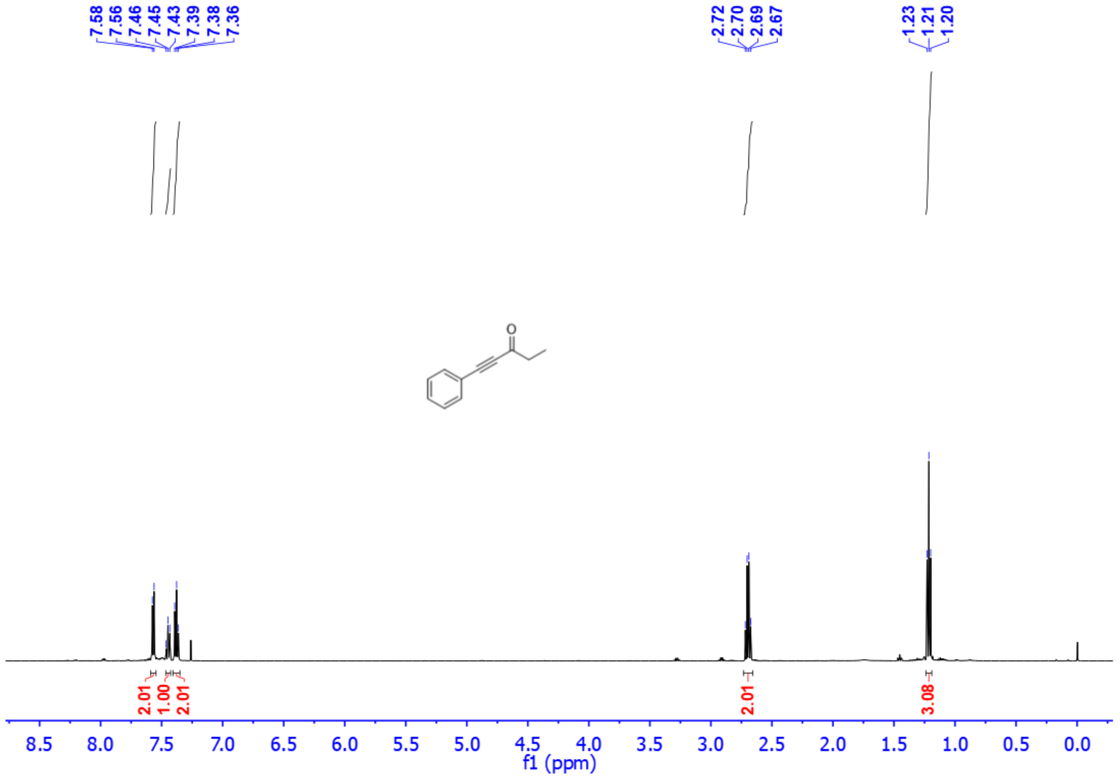

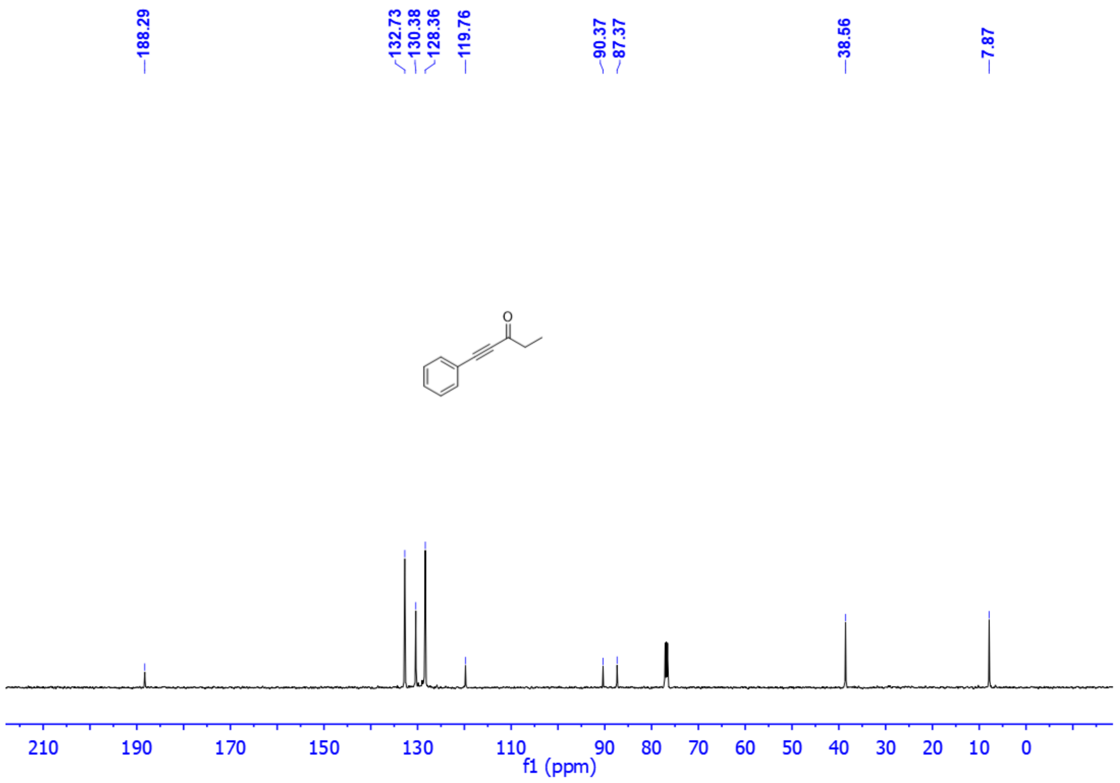

The ^{1}H and ^{13}C NMR spectra of compounds (4s)

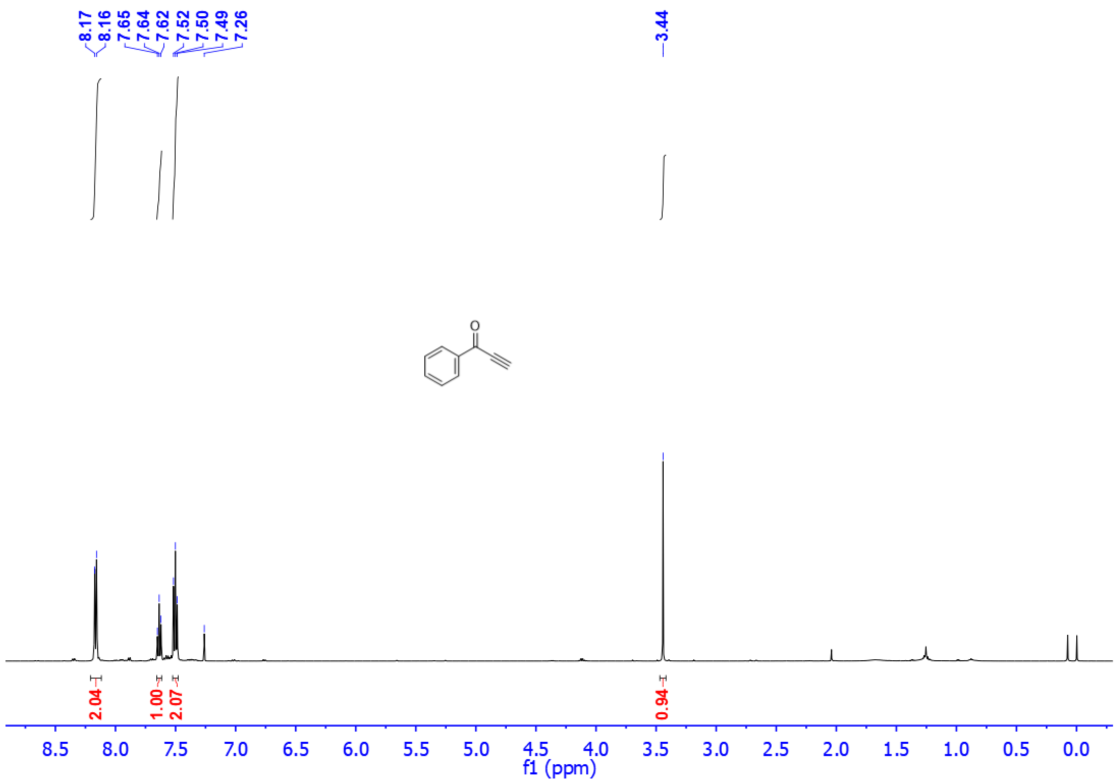

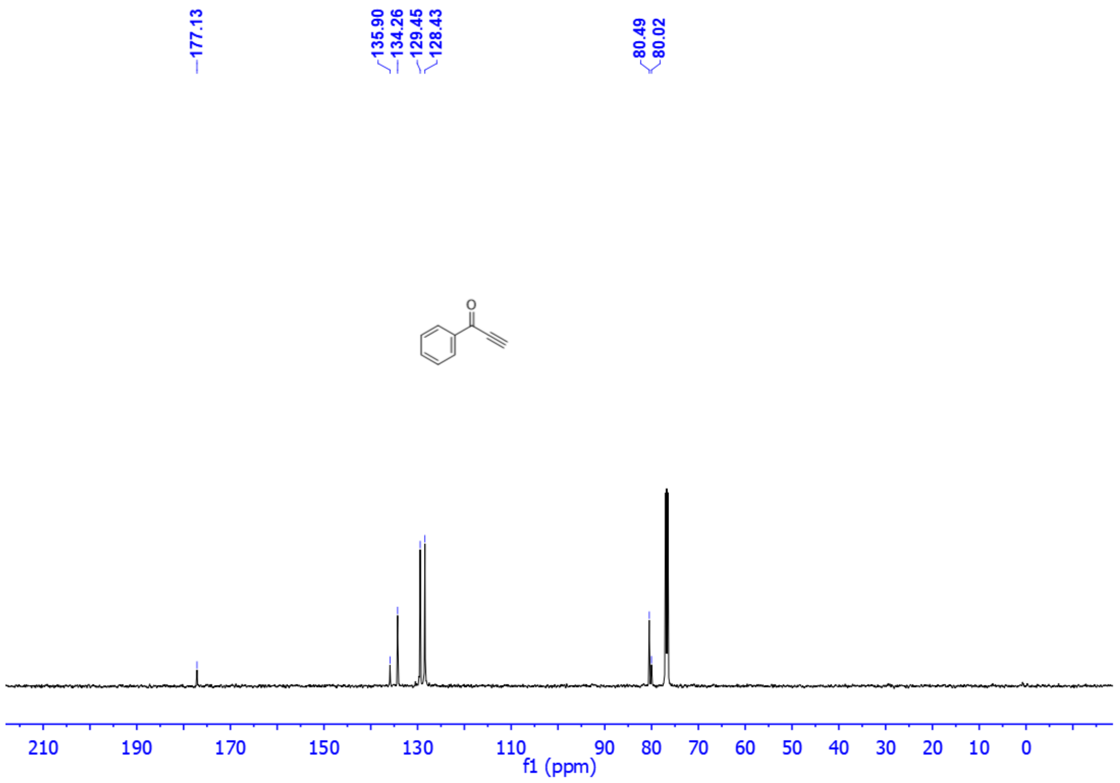

The ^{1}H and ^{13}C NMR spectra of compounds (4t)

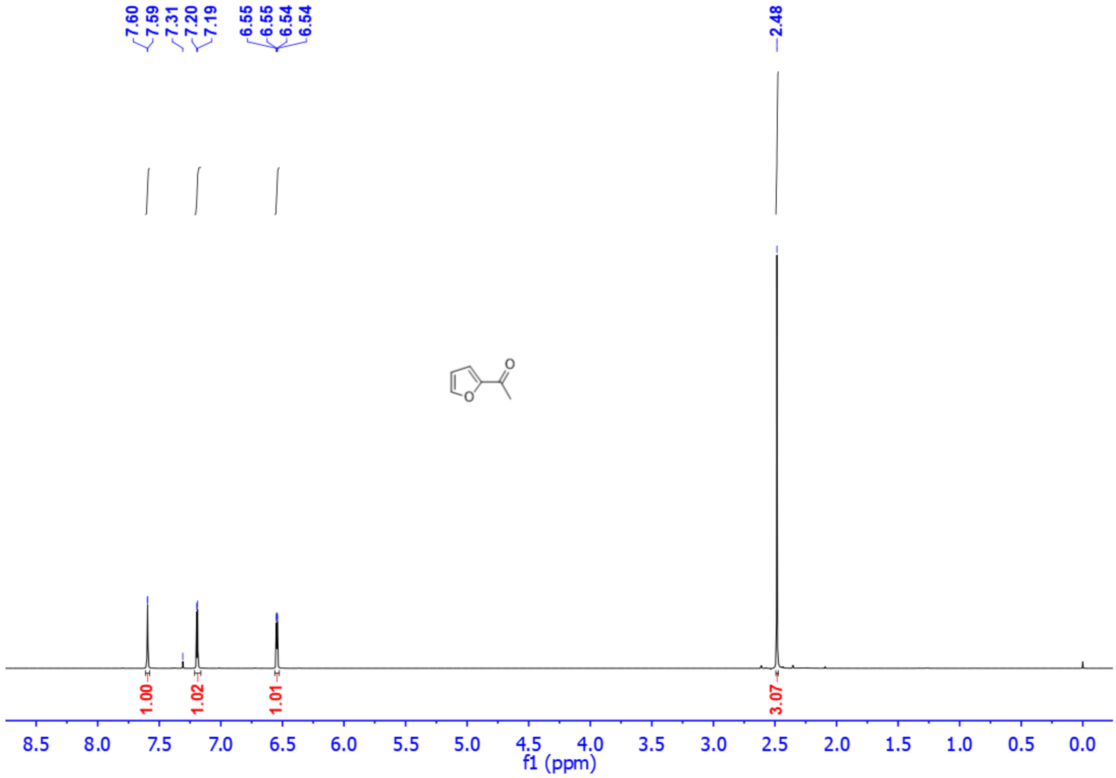

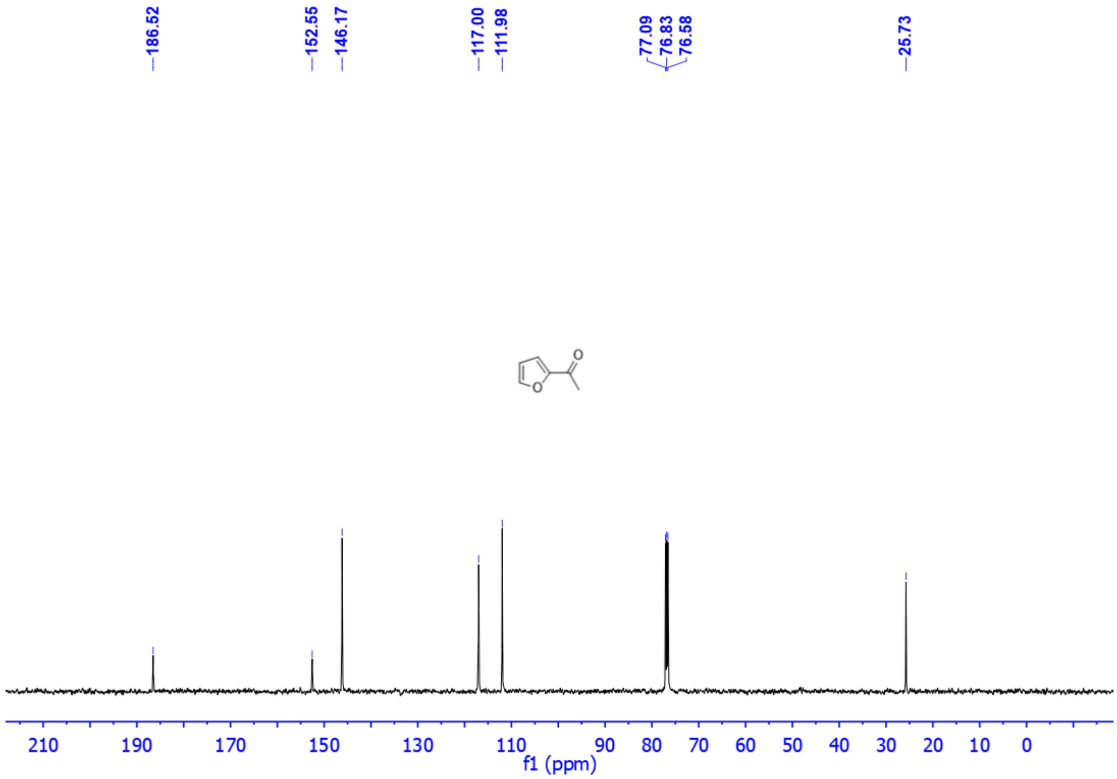

The ^{1}H and ^{13}C NMR spectra of compounds (4u)

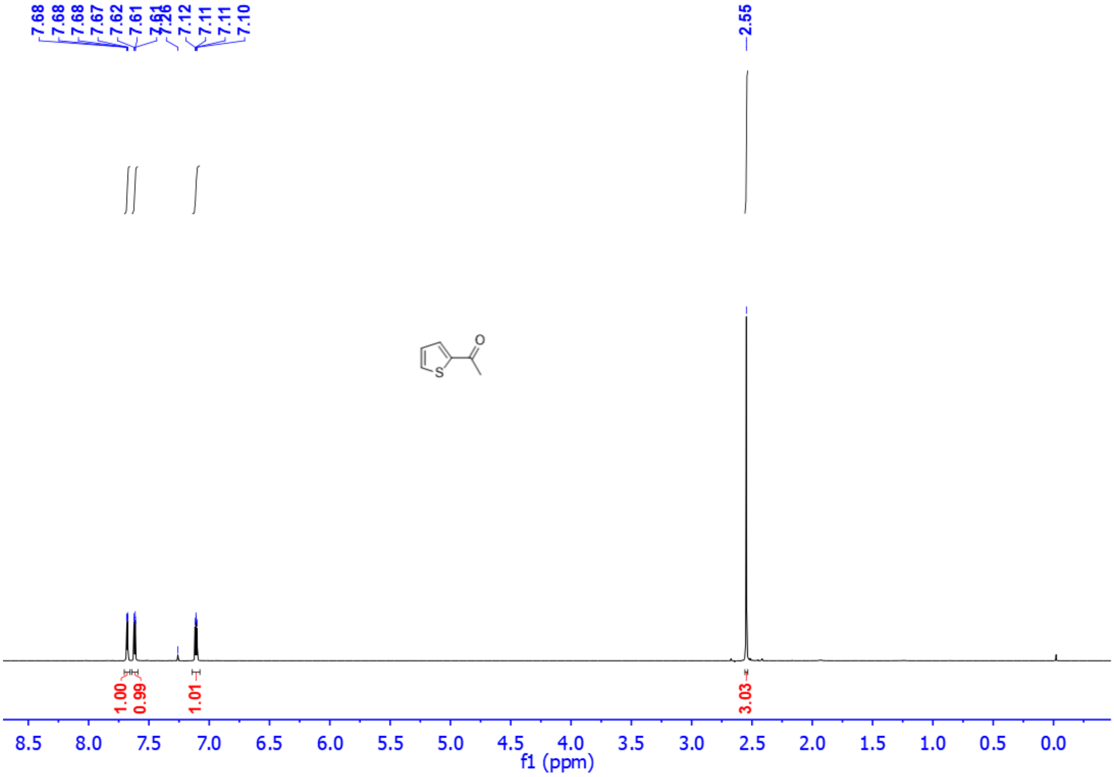

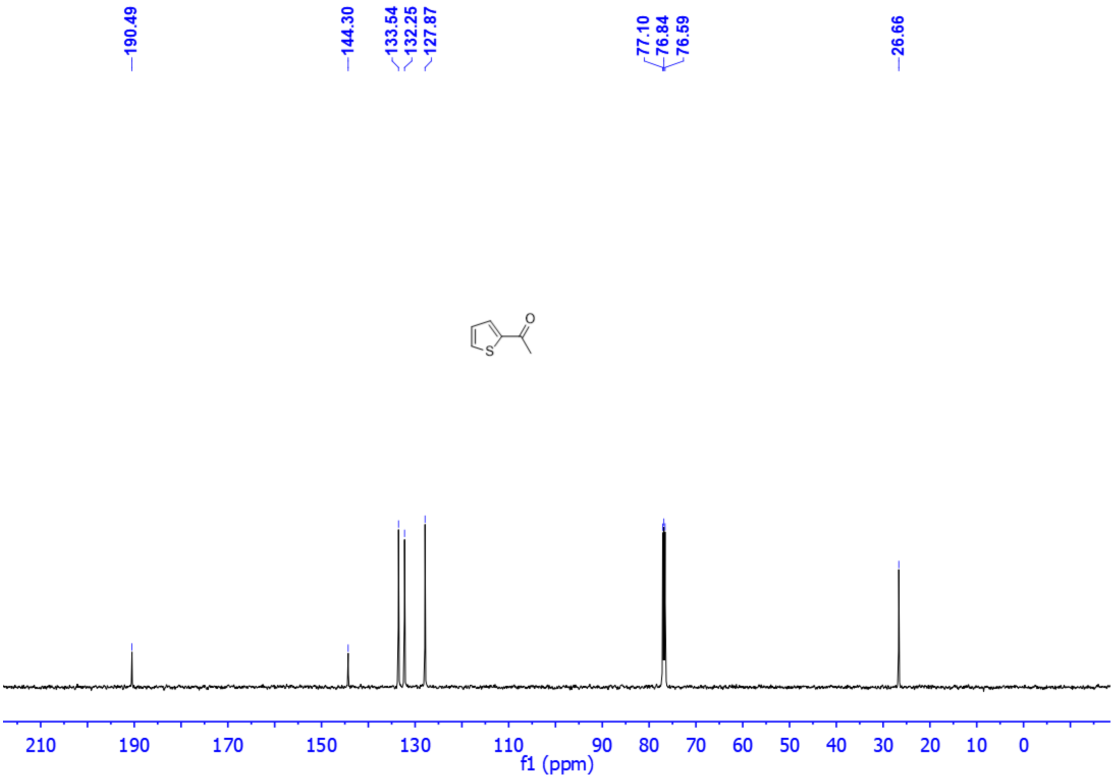

The ^{1}H and ^{13}C NMR spectra of compounds (4v)

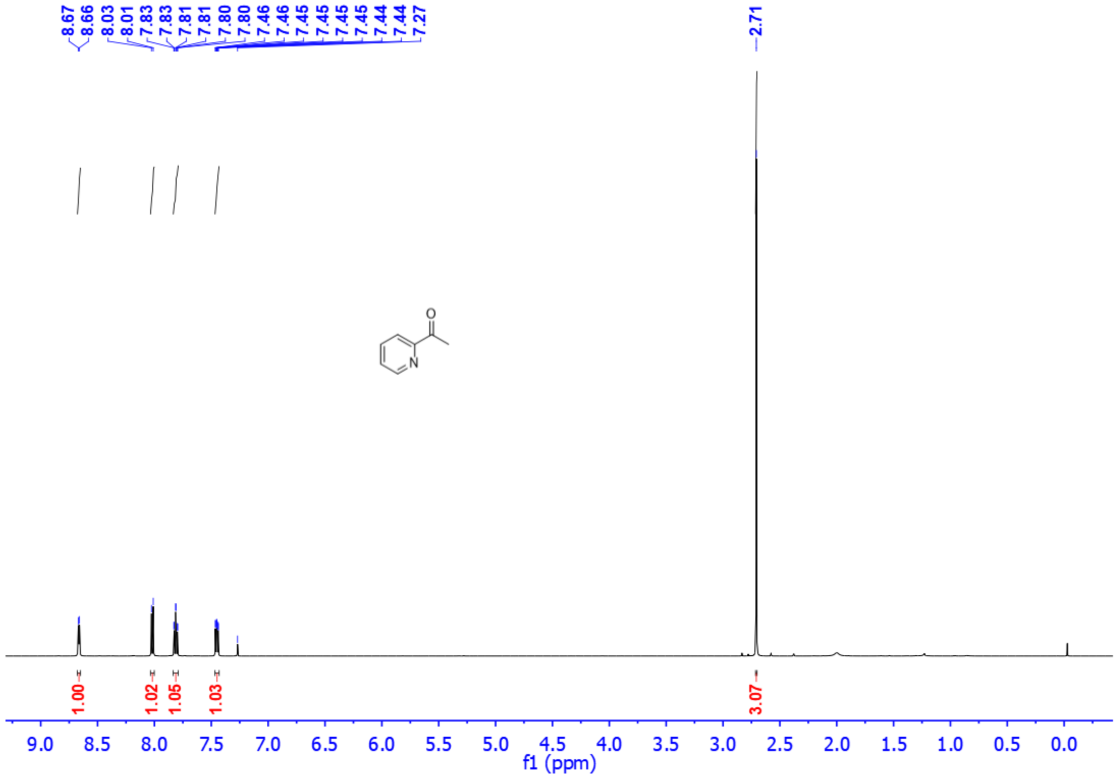

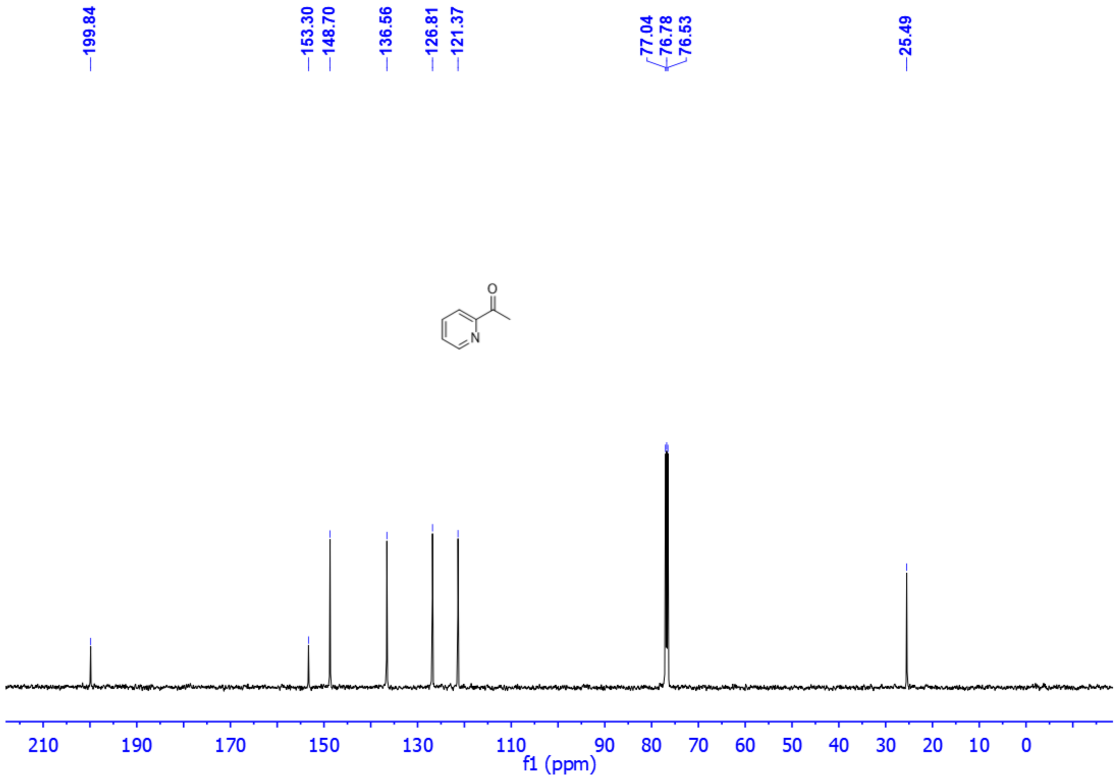

Supplement: Supplementary file 1 [file molecules-27-03727-s001.zip › molecules-1755847-supplementary.pdf]
